# Clinical features and comorbidity in very early-onset schizophrenia: a systematic review

**DOI:** 10.3389/fpsyt.2023.1270799

**Published:** 2023-12-13

**Authors:** Michelangelo Di Luzio, Maria Pontillo, Marianna Villa, Anna Gaia Attardi, Domenica Bellantoni, Cristina Di Vincenzo, Stefano Vicari

**Affiliations:** ^1^Child and Adolescent Neuropsychiatry Unit, Bambino Gesù Children's Hospital, IRCCS, Rome, Italy; ^2^Life Sciences and Public Health Department, Catholic University, Rome, Italy; ^3^Department of Human Pathology of the Adult and Developmental Age "Gaetano Barresi", Unit of Child Neurology and Psychiatry, University of Messina, Messina, Italy; ^4^School of Child Neurology and Psychiatry, PROMISE Department, University of Palermo, Palermo, Italy

**Keywords:** very early-onset schizophrenia (VEOS), adult-onset schizophrenia, clinical features, comorbidities, psychotic symptoms

## Abstract

**Background:**

Very early-onset schizophrenia (VEOS) is a form of schizophrenia that manifests before the age of 13 years and is characterized by the presence of positive, negative, and disorganized symptoms. The condition is exceptionally rare and, to date, limited studies have been conducted, resulting in incomplete information about its clinical features.

**Methods:**

The present study involves a systematic review of the existing literature regarding the clinical features and comorbidities of VEOS.

**Results:**

The first search retrieved 384 studies. Of these, 366 were removed following the application of exclusion criteria, resulting in 18 studies for the final set.

**Conclusion:**

The results highlight that VEOS shares similarities with early-onset and adult-onset schizophrenia but also exhibits distinct and recognizable characteristics, including a more severe clinical profile (particularly in females), increased visual hallucinations, and high comorbidities with neurodevelopmental disorders. These findings may support clinicians in formulating early diagnoses and developing effective treatment strategies for pediatric and adolescent patients with psychosis.

## 1 Introduction

Schizophrenia is a major psychiatric disorder that is characterized in the *Diagnostic and Statistical Manual of Mental Disorders, Fifth Edition* by the presence of positive (i.e., delusions, hallucinations), negative (i.e., blunted affect, social withdrawal, anhedonia), and disorganized (i.e., disorganized speech and behavior) symptoms (1). Very early-onset schizophrenia (VEOS) is a specific form of schizophrenia that manifests very early in life, typically before the age of 13 years (2, 3). The disorder shares diagnostic criteria with both adult-onset schizophrenia (AOS), which manifests after the age of 18 years, and early-onset schizophrenia (EOS), which is characterized by symptom onset between the ages of 13 and 18 years (2–6). Of note, some authors refer to VEOS as childhood-onset schizophrenia (COS). The National Institute of Mental Health (NIMH) in the United States defines that COS is present when the diagnostic criteria for schizophrenia are met prior to the age of 13 years, alongside a premorbid IQ higher than 70 and a lack of any other neurological disorder (7, 8). In this text, we will use the term VEOS interchangeably with COS and consider adolescent-onset schizophrenia (AdOS) equivalent to EOS.

Several clinical studies have emphasized the connection between VEOS, EOS, and AOS (3, 9, 10). In particular, some studies have underlined that the genetic risk factors are similar across the three disorders (11). In support of this claim, neuroimaging studies have revealed comparable gray matter alterations in VEOS, EOS, and AOS patients, although the alterations in VEOS patients manifest as more severe. Evidence from these studies reveals a complex and extensive pattern of both gray and white matter changes, particularly in patients whose alterations began during childhood. This suggests a form of altered neurodevelopment rather than regression resulting from the onset of psychiatric pathology (12). Furthermore, genetic studies have also highlighted the possibility that psychiatric disorders, including schizophrenia and bipolar disorder, may belong to the same psychopathological continuum as neurodevelopmental disorders (13). Within this framework, VEOS can be considered one of the possible “missing links” in the psychopathological continuum between neurodevelopmental disorders and adult schizophrenia.

VEOS is an exceptionally rare disorder, and there is a lack of comprehensive epidemiological data due to the limited research conducted to date. However, a study by the NIMH indicated a VEOS prevalence rate of 1 in 40,000 (7). Another study examining the entire English population revealed an incidence rate of hospitalization for VEOS of 0.03 per 100,000 among males and 0.01 per 100,000 among females, with no significant difference between sexes (14). Moreover, the diagnosis of VEOS can be challenging for several reasons. First, as reviewed by Giannitelli et al. (15), organic causes that can lead to psychotic symptoms in childhood must be excluded, as these may have specific treatments and solutions. Second, positive psychotic symptoms (e.g., hallucinations, unusual/bizarre thought contents) can be relatively common in pre-adolescents, before showing spontaneous remission. Thus, such symptoms may not progress into a full-blown psychotic disorder or another psychiatric condition, particularly if they occur as isolated symptoms in individuals younger than 12 years (16, 17). Indeed, the prevalence of such symptoms in the general child population is significantly higher than the relatively low prevalence of VEOS. Specifically, a review and meta-analysis by Kelleher et al. (17) revealed an average prevalence of psychotic symptoms in children aged 9–12 years and adolescents aged 13–18 years of 17 and 7.5%, respectively. Third, VEOS is often accompanied by high rates of co-occurring neurodevelopmental disorders, either as full syndromes or sub-threshold conditions. Driver et al. (7) observed that, among the NIMH study cohort, 72% of the VEOS patients exhibited socio-relational difficulties, 55% demonstrated academic difficulties, 50% reflected language difficulties, 44% displayed motor difficulties, and up to 20% had a comorbid diagnosis of autism spectrum disorder (ASD) (7). Finally, the characteristics of VEOS, compared to AOS, are not well-defined and have not yet been extensively studied. There is a scarcity of research on this topic, and the limited number of studies that have been conducted have generally involved small sample sizes, due to the rarity of VEOS and challenges associated with differential diagnosis (i.e., distinguishing VEOS from mood disorders, multidimensional impairment, and childhood anxiety disorders) (18). Furthermore, it can be particularly challenging to discriminate between schizophrenia and schizophrenia spectrum disorders. In this context, it is possible that studies may exhibit heterogeneity in the inclusion of patients within the VEOS diagnosis. In fact, they may also include patients with the broad dimension of schizophrenia spectrum disorder (brief psychotic disorder, schizotypal disorder, delusional disorder, schizophrenia, schizophreniform disorder, and schizoaffective disorder) (1), treating them collectively as VEOS. However, it is important to acknowledge the potential heterogeneity within this group and the need for further research to distinguish specific subtypes and their unique characteristics.

For the present review, we hypothesized that VEOS would share clinical features with EOS and AOS, while also displaying distinct and identifiable characteristics. Accordingly, a comprehensive review was conducted to consolidate the scientific literature regarding the clinical characteristics and comorbidities of VEOS. The aim was to summarize the major findings to date, providing clinicians with more tools to support their diagnostic process and development of tailored treatment plans, and enhance the overall understanding of VEOS.

## 2 Methods

### 2.1 Search strategy

This study is based on a PubMed/MEDLINE exploration for studies published from the beginning of the database until February 28, 2023, employing the following search terms: “Very early-onset schizophrenia” OR “Childhood-onset schizophrenia.” The entire research team reached a consensus on the search approach and collectively contributed to the examination of the literature. The chosen articles fulfilled the subsequent eligibility criteria: (1) they constituted original research studies; (2) they included subjects with a diagnosis of VEOS (< 13 years), as assessed by the Structured Clinical Interview for Diagnostic and Statistical Manual of Mental Disorders third/fourth/fifth edition (DSM-III/IV/5) or the International Classification of Diseases ninth/tenth edition (ICD-9/10); (3) they separated data for VEOS; and (4) they utilized questionnaires or interviews to assess anamnestic data, clinical features such as signs and symptoms, and comorbidities.

### 2.2 Eligibility and study selection

The following studies were not considered: (1) reviews and meta-analyses (nevertheless, the reference lists of these studies were scrutinized to identify potentially eligible studies that might have been missed during the initial database search) (i.e., “Review”); (2) case reports or case series (i.e., “Case”); (3) studies that did not assess individuals with schizophrenia onset before 13 years (i.e., “No VEOS”); (4) studies that did not focus the assessment on clinical features (i.e., “No Clinical”); (5) qualitative studies not supported by statistical analysis (i.e., “No Data”); (6) research that did not offer distinct data for VEOS subjects in comparison to EOS, AOS, healthy controls, or individuals with different psychiatric diagnoses (i.e., “Lumping); (7) studies unrelated to the pertinent topic (i.e., “Unrelated”); (8) editorials, letters to the editor, opinion articles not supported by data (i.e., “Letters”); (9) protocols and ongoing studies; (10) corrections to existing article; and (11) studies for which no English translation was available (i.e., “No English”). The criteria for including and excluding studies, established through two rounds of the Delphi method, gained unanimous acceptance from all authors. The research adhered to the guidelines outlined in the Preferred Reporting Items for Systematic Reviews and Meta-Analyses (PRISMA) (19). The online Supplementary material comprises the PRISMA checklist and flowchart, along with comprehensive results and data regarding included/excluded studies (refer to Supplementary Figures S1, S2 and Supplementary Table S1).

### 2.3 Data extraction and synthesis

Information extracted from the chosen articles was systematically recorded in a standardized spreadsheet. Precisely, the subsequent variables were recorded: primary author, publication year, sample size, participant age, sex ratio (male/female), study design (incorporating interviews, tests, or questionnaires employed), and outcomes pertaining to key clinical characteristics among individuals diagnosed with VEOS. A summary of the included studies is included in Table 1.

**Table 1:** Summary of included studies.

Study	Population	Design	Results	Observations
Cheng et al. (20)	216 ids with COS (F 126; M 90; x̄ at onset =10.66 ± 1.79). 366 ids with AdOS (F 221; M 145; x̄ at onset =14.17 ± 1.07).	RS. CA, PANSS.	No difference in sex, days of hospitalization, psychiatric family history, comorbidity, DUP, and PANSS total score at admission for AdOS and COS. COS had a ↑ illness course, ↓ PANSS positive score upon admission and PANSS reduction rate, and a ↑ PANSS negative score upon admission and PANSS total score on discharge than AdOS (*p* < 0.05). COS had ↑ insidious onset (*p* < 0.01), bizarre behaviors, impulsive behaviors, visual hallucinations, and formal thought disorder and ↓ delusions than AdOS (*p* < 0.05). No significant differences in the incidence of hallucinations, negative symptoms or early non-specific symptoms between the two groups. COS showed ↓ treatment efficacy than AdOS (*p* < 0.05).	Results about clinical manifestations and severity of illness in COS with respect to SCZ in older ids are in line with previous studies. Instead, the absence of differences in DUP and in premorbid neurodevelopment alterations appear to be in contrast with the literature. This study presents data on a large group of COS.
Galitzer et al. (21)	20 ids with COSS (F 13; M 7; x̄ at admission = 11 ± 1.83). 191 ids non-COSS (F 82; M 109; x̄ at admission =10.78 ± 1.65)	RS. CA, CGAS.	COSS had ↓ CGAS scores at admission compared to non-COSS (*p* = 0.006), while scores at discharge were not statistically different between the groups. COSS were more likely compared to non-COSS to be on medication at discharge (*p* = 0.009) and on medication with antipsychotics at any point (*p* = 0.001) and at discharge (*p* = 0.001). These results were more pronounced in F and in older (> 11.16 y) COSS. Older COSS were in 90.1% F. In addition, F were not in education at admission (*p* = 0.025) and had ↑ duration of admissions (*p* = 0.016) and ↓ CGAS at discharge. No differences in comorbidity between groups.	This study was poorly informative about clinical features. Highlighted worse functioning in COSS, in particular at admission. Interestingly, F showed worse functioning and an older age compared to M. The sample of COSS was small.
Coulon et al. (22)	22 ids with VEOS (F 8; M 14; x̄ at onset = 9.55 ± 2.5); 154 ids with EOS (F 38; M 116; x̄ at onset = 15.9 ± 1.2); 551 ids with AOS (F142; M 409; x̄ at onset = 23.7 ± 5.9)	RS. CA, PANSS, CDRS, EHI, GAF, WAIS III/IV, NART.	VEOS had a fourfold ↑ DUP than the EOS (*p* < 0.0001) and an eightfold ↑ DUP than the AOS (*p* < 0.0001). VEOS had ↑ PANSS scores for the psychopathology general score (*p* = 0.021) and total score (*p* = 0.041) than EOS and AOS. VEOS had ↓ educational levels than EOS and AOS (*p* < 0.0001). No significant differences in neuropsychological scores among the three groups, and no difference in the premorbid IQ scores between the three groups. VEOS exhibited ↑ history of learning disabilities than AOS (*p* = 0.020) and ↓ right-handedness quotient than AOS (*p* = 0.048).	In the present study, a ↑ DUP and a ↑ illness severity are confirmed for VEOS with respect to EOS and AOS. These results were in line with previous literature. However, no differences were noted for positive or negative symptoms between the groups as in other studies.
Craddock et al. (23)	125 ids with COS (F 60; M 65; x̄ at onset = 9.90 ± 2.03)	LS. CA, SAPS, SANS, BPRS, CGAS, KSADS, ASQ, WIS.	A two-factor solution containing positive and negative dimensions was found from CFA and 3-cluster solution using K-means cluster analysis. The three groups had low scores on both dimensions (LM), high negative scores with low positive scores (HN), and high scores on both dimensions (HM). LM had ↑ full-scale IQ than HN and HM (*p* = 1.50E-03). LM had ↑ CGAS scores than HM (*p* = 2.13E-09), while HN showed intermediate scores. A trend was observed for age of onset, with HN being older at onset than LM and HM. LM showed a trend in comorbidity with behavioral disorders (ADHD, ODD, CD) than HN and HM.	This study shows the importance of negative symptoms in COS. Moreover, it highlights the relationship between psychotic symptom severity, worse functioning, and lower IQ. Results are similar to those found on EOS and AOS. A possible secondary role for disorganized symptoms was noted.
Ordonez et al. (24)	133 ids with COS; F 61, x̄ at onset = 10.29 ± 1.63; M 72, x̄ at onset = 9.51 ± 2.28.	LS. CA, SAPS, SANS, BPRS, CGAS, KSADS, ASQ, WIS, SIM, AIMS	F had ↓ verbal IQ than M (*p* = 0.03). M had a lower age of onset than F (*p* = 0.03). M showed ↑ rates of comorbidity with PDD and ADHD the F. No differences between groups in most clinical measures or in premorbid abnormalities across academic, language, or motor domains.	These results point out some sex differences in COS. In particular, F showed ↓ IQ and this, in light of the literature, might suggest worse functioning and most severe psychotic symptoms in F with COS.
Greenstein et al. (8)	85 ids with COS (F 38; M 47; x̄ at onset =9.92 ± 2.06), 53 ids with AD (F 18; M 35; x̄ at onset =8.33 ± 2.35).	LS. CA, SAPS, SANS, BPRS, CGAS, KSADS, WIS, NIMHGS	COS had ↑ scores for positive and negative symptoms in SANS (*p* < 0.0001), SAPS (*p* < 0.0001), BPRS (*p* = 0.0002), and NIMHGS PsyS (*p* < 0.0001) and ↓ scores in IQ (*p* = 0.0004), CGAS (*p* < 0.0001) and NIMH DepS (*p*<. 0001)/AnxS (*p* = 0.015) than AD. COS were older at age of onset than AD (*p* < 0.0001). Results of multiple logistic regression, two predictor models including only NIMHGS PsyS and DepS, showed: PPV = 91.34%, NPV = 55.20%, sensitivity = 78.71%, specificity = 77.56%, overall accuracy = 78.42%, AUC = 87.12%. These results indicated that ↑ psychosis ratings and ↓ depression ratings combine to increase the probability that a patient has COS.	The authors purpose a worksheet to be used in clinical settings to determine the likelihood that a child or adolescent has COS. These results highlight the difference between depressive symptoms and negative psychotic symptoms. The severity, not just the presence, of psychotic symptoms differentiates COS children from AD children.
David et al. (25)	117 ids with COS (F 50; M 67; 24 NVH with x̄ at onset =10.7 ± 1.59; 94 VH with x̄ at onset =9.7 ± 2.1)	LS. CA, SAPS, SANS, CGAS, WIS.	COS had: 95% auditory Ha, 80.3% visual Ha, 60.7% somatic/tactile Ha, and 30% olfactory Ha. There was a considerable overlap between all the Ha modalities: all ids with visual Ha had auditory Ha (not vice versa) and all ids with somatic/tactile and olfactory Ha had visual and auditory Ha. VH compared to NVH had an earlier age of psychosis onset (*p* < 0.05), younger age at assessment (*p* < 0.01), ↓ full-scale IQ (*p* < 0.01), ↓ CGAS, and ↓ duration of illness from age of 1st symptom onset (*p* < 0.01).	Auditory Ha also appears to be a fundamental psychotic symptom in COS. However, visual Ha is highly represented and could be considered an index of COS severity.
White et al. (26)	26 ids with COSS (F 9 M 17; age x̄ = 14.8 ± 2.9) 37 HC (M 22 F 15, age x̄ = 14.5 ± 3.2)	LS. SIRP	COS patients performed worse than HC within all three age groups in both verbal (*p* < 0.0001) and visuospatial modalities (*p* < 0.001). The trajectory of the verbal SIRP showed a disproportionately lower performance in the VEOS group compared with the older two age groups (*p* < 0.002).	According to adults’ data, verbal and visuospatial modalities are deficient in patients with COS. This study highlighted that the early onset SCZ, at a time when verbal short-term memory is rapidly maturing, could impair cognitive performance and, in particular, verbal performance.
Mattai et al. (27)	61 ids with COS divided into two groups: “good sleepers” (> 6 h, *n* = 30, M 10 F 20, x̄ = 10.93 ± 2.18) and “poor sleepers” (< 6 h, *n* = 31, M 16 F 15 x̄=10.50 ± 3.32).	LS. CGAS, CGI, BPRS, SAPS SANS, Psyc-BH, Mania-BH, Ansx-BH, Dep-BH.	“Good sleepers” showed better functioning in BPRS (*p* = 0.008), SAPS (*p* = 0.018), and SANS (*p* = 0.020) upon admission. “Poor sleepers” showed ↑ BH-Anxiety scores (*p* = 0.042) upon admission. “Poor sleepers” without medications had a significantly ↑ score in SAPS (*p* = 0.017) SANS (*p* = 0.0125), BH-Anxiety (*p* = 0.014), CGAS (*p* = 0.08), and BPRS (*p* = 0.07). “Poor sleepers” had ↑ scores in SAPS and SANS at admission (Pearson’s *p* < 0.001 and Spearman’s (*p* = 0.003) respectively) and during the medication wash-out period (Pearson’s *p* = 0.01 and Pearson’s *p* = 0.002).	COS patients suffer from significant sleep disturbances and sleep disturbance is closely related to symptom severity. This supports the idea that subjects suffering from more severe symptoms upon hospital admission could be supposed to have significant sleep disturbance, which would continue with discontinuation of the medication
Biswas et al. (28)	15 ids with COS (F 8; M 7; x̄ at onset = 12.25 ± 1.16), 20 AdOS (10 M,10F; x̄ at onset = 21.81 ± 2.31) and 20 with AOS (M10, F10; x̄ at onset = 31.45 ± 8.35)	LS MISIC, PGI, BVMG, NBT, PANSS	The COS group had significantly ↑ PANSS Positive scores (*p* < 0.01) and ↑ PANSS Negative scores (*p* < 0.001) and PANSS General Psychopathology scores (*p* < 0.001) than AdOS and AOS. The COS group performed ↓ on all the subtests of memory PGI (*p* < 0.001) except for recent memory and had ↑ error scores (*p* = 0.001) and ↑ dysfunction rating scores (*p* < 0.05) on BVMG and NBT.	This study supports the hypothesis that the earlier the onset and greater the severity of illness and neuropsychological deficits, in particular, verbal learning, visual learning, overall memory, and visuospatial and visuomotor organization deficit.
Abu-Akel et al. (29)	32 ids with COS (M 27, F 5; age x̄ 10.34 ± 1.56) medicated (*n* = 15),unmedicated (*n* = 17) and 34 HC (M27 F7; age x̄ 9.28 ± 2.07)	RS K-FTDS, WISC-R	COS ids, treated and untreated, had a significant inappropriate response to the Yes/No (*p* < 0.001) and Wh (*p* < 0.02) questions, compared to HC. In terms of increased use of speech functions, the medicated group showed ↑ no responses to both Yes/No (*p* < 0.003) and Wh- questions (*p* < 0.02), and inappropriate responses to both Yes/No (*p* < 0.002) and Wh- questions (*p* < 0.01), compared to HC. Conversely, the unmedicated group gave significantly ↑ inadequate responses to Yes/No questions (*p* < 0.03) than HC. However, regarding the decrease in the use of speech functions, the drug-treated group gave ↓ direct responses (Yes/No, *p* < 0.06; Wh- questions, *p* < 0.001) and ↓ additional responses (Yes/No, *p* < 0.002; Wh- questions, *p* < 0.005) compared to HC. The unmedicated group differed only in the use of fewer direct responses to Wh- questions (*p* < 0.01) than HC. The medicated group differed from the unmedicated group only by a higher use of supplementary answers to Yes/No questions (*p* < 0.05) No correlations of Full-Scale, Verbal, or Performance IQ with any of the speech function variables. However, SCZ ids presented a significant correlation between the WISC-R Distractibility factor score with no responses (*p* < 0.04), direct responses (*p* < 0.05), implied responses (*p* < 0.005), and inappropriate responses to Yes/No questions (*p* < 0.07).	This study showed that the SCZ group differed significantly from the HC group in the use of speech functions. The medicated patients seem to have a wider range of abnormal uses of speech functions than the unmedicated patients: less responsive and less likely to generate speech after their initial response to questions (i.e., supplementary responses) compared with the unmedicated patients. In addition, speech functions appear to be associated with specific (WISC-R Distractibility) rather than with global cognitive deficits.
Frazier et al. (30)	28 ids with VEOS (F 14; M 14; x̄ at onset of psychosis = 10.2 ± 1.7); 20 HC (F 11; M 9; in pubertal age)	RS FSIQ, Mean Tanner Stage	A significant correlation was found between the age of onset of secondary sexual characteristics and the age of onset of psychosis in F (*p* = 0.002) but not in M. Additionally, the onset of menarche did not show any relationship with the onset of psychosis (*p* = 0.41.). The study also found that the ages of onset of pubertal changes were similar in M and F siblings of the study participants, and there were no significant differences between the ages of onset of menarche in F VEOS and their sisters (*p* = 0.22).	This study found an absence of a clear relationship between onset of psychosis and indices of sexual development for VEOS.
Caplan et al. (31)	32 ids (F 5; 27 M; x̄ 10.3 ± 1.56) with VEOS (18 medicated; F4; M 14; age between 7.4 and 12.3 y; 14 unmedicated; F 1; M 13; age between 8.5 and 12.2 y); 47 HC (F 12; M 35; x̄ 9.3 ± 2.03).	LS Interview for Childhood Disorders and Schizophrenia, Story Game, K-FTDS, WISC-R	HC used significantly ↑ referential revision (*p* < 0.0005), word choice revision (*p* < 0.05), false starts (*p* < 0.003), and fillers (*p* < 0.003) than the medicated VEOS. The medicated VEOS used ↓ referential cohesion, conjunctions, and words per clause than the HC and ↓ referential revision (*p* < 0.05), postponement (*p* < 0.01), and fillers (*p* = 0.006) than the unmedicated patients. VEOS had ↑ illogical thinking scores than HC (*p* < 0.002). VEOS with loose associations used ↑ false starts (*p* < 0.01) and fillers (*p* < 0.01) than those without loose associations. Within the VEOS group, there was a significant correlation of the WISC-R Distractibility factor score with false start (*p* < 0.006), repetition (*p* < 0.004), and loose associations (*p* < 0.006). There was a diagnostic effect for the following cohesive variables: referential cohesion (*p* < 0.001), conjunction (*p* < 0.0001), unclear/ambiguous reference (*p* < 0.02), and verbal productivity (words per clause) (*p* < 0.001)	This study represents the first attempt to investigate self-initiated repair measures in VEOS, along with the correlation with clinical indicators of formal thought disorder, linguistic cohesion metrics, and cognitive distractibility score. The study’s findings suggest that, alongside formal thought disorder, diminished employment of repair strategies, lower employment of cohesive elements, and decreased verbal output for expressing thoughts could potentially signify adverse manifestations in VEOS.
Alaghband-Rad et al. (32)	23 ids with VEOS (15 M and 8 F; x̄ at onset of psychosis < or = 12).	RS K-SADS-E, DICA-P, DICA-C, PAS, ADI-R, FSIQ	There were more delays for crawling in M (*p* = 0.04). Delays were most striking for language development; the mean age of first sentence was 26.5 months (±7.4), which is significantly delayed compared which is significantly delayed compared with the adult cases (*p* < 0.0001). Ids showed low-normal IQ and inconsistent cognitive decline (*p* = 0.48.). In general, the sample showed quantitative and qualitative abnormalities, similarly to previous reports on VEOS: 36% with at least some features of PDD (autism and transient motor features) and 30% with ADHD.	The findings indicate greater premorbid abnormalities in VEOS. This, together with the chronicity and severity of childhood cases, indicates that VEOS may be a more severe form of the disorder.
Hollis et al. (33)	18 ids with VEOS (F 5; M 13; age between 7 and 13 y) and 43 ids with EOS (F 22; M 21; age between 14 and 17 y); 61 HC coupled for age and sex with.	RS CA	The VEOS and EOS showed no significant differences in the occurrence of psychotic symptoms. The disorder of language production is significantly ↑ in VEOS (*p* = 0.012), and the difference in disordered language comprehension with EOS is not statistically significant. Among disturbances in motor development, only “restlessness and fidgetiness” were significantly ↑ in the EOS (*p* < 0.02). VEOS had ↑ insidious start, but this was not statistically significant (*p* = 0.07).	This study found that language impairments were more common in VEOS than in EOS and were independent of sex. A limitation of the study is that the comparison with HC is made with all schizophrenic patients without distinguishing between VEOS and EOS.
Caplan et al. (34)	29 ids with VEOS (F 6; M 23; x̄=10.2 ± 1.6); 10 schizotypal (F 3; M 7; x̄=9.3 ± 1.4); 54 HC (F 12; M 42; age between 5 and 12.5 y)	LS CA, K-FTDS, WISC-R	VEOS (*p* < 0.0002) and schizotypal ids (*p* < 0.0001) both had significantly ↑ illogical thinking and total FTD scores than HC. In total, 70% of VEOS (*p* < 0.0001) and 64% of the schizotypal children (*p* < 0.003) had loose associations scores above zero. The data among VEOS, schizotypal children, and HC demonstrated that IQ did not affect the diagnostic differences in the K-FTDS scores of patients and HC.	The K-FTDS could be used to detect FTD in children at risk of schizophrenia. Also of note is the correlation found between IQ and FTD in VEOS.
Caplan et al. (35)	31 ids with VEOS (F 6; M 25; x̄ 10.2 ± 1.5)	LS CA, K-SADS, K-FTDS, WISC-R, Span of apprehension task	Illogical thinking and loose associations were not significantly correlated (*p* < 0.5). Loose associations were negatively and significantly correlated with FSIQ (*p* < 0.01) and the WISC-R distractibility factor (*p* < 0.02) but not with the verbal IQ (*p* < 0.2) and performance IQ sub-scores (*p* < 0.05). After partializing out the variance from the distractibility factor scores, loose associations were not significantly correlated with FSIQ (*p* < 0.2) and performance IQ (*p* < 0.6). Illogical thinking was not significantly associated with FSIQ (*p* < 0.4) verbal IQ (*p* < 0.7), performance IQ (*p* < 0.3), or distractibility factor scores (*p* < 0.3). VEOS with partial report span of apprehension scores had ↑ illogical thinking scores (*p* < 0.05).	This study found that illogical thinking and loose associations reflect different aspects of attention/information processing in VEOS. There were significant associations between illogical thinking and the span of apprehension and between loose associations and distractibility. Neuropsychological impairments could have a role in clinical manifestations and in the severity of the disorder.
Watkins et al. (36)	18 ids with VEOS (F 5; M 13; age at onset of psychosis <10 y): 7 with a history of autism (SA group) and 11 with a history of COPDD (S group).	RS CA, K-SADS, CBCL, CPRS-D, WISC-R	The SA group had a significantly earlier onset of SCZ than the S group (*p* < 0.05). In total, 7 of the 15 DSM-III symptoms of autism and COPDD were present in the SA group at significantly ↑ levels (*p* < 0.05) than in the S group between 31 months and 6 years.	This article investigates the comorbidities between early schizophrenia and other psychopathological disorders of childhood and their evolution over time. In particular, it highlights the association between VEOS and neurodevelopmental disorders.

AD, alternate diagnosis; ADI-R, Autism Diagnostic Interview; ADHD, attention deficit hyperactivity disorder; AdOS, Adolescent-Onset Schizophrenia; AFH, age of first hospitalization; AFS, age of first non-specific psychiatric symptoms; AIMS, Abnormal Involuntary Movement Scale; AnxS, Anxiety Score; AOS, Adult-Onset Schizophrenia; ASQ, Autism Screening Questionnaire; AUC, area under the curve; BH-Bunny Hamburg; BPRS, Brief Psychiatric Rating Scale; BVMG, Bender Visuo-motor Gestalt test, CA, clinical and anamnestic assessment; CBCL, Achenbach Child Behavior Checklist; CD, conduct disorder; CDRS, Calgary Depression Rating Scale for Schizophrenia; CFA, confirmatory factor analysis; CGAS, Children’s Global Assessment Scale; CGI, Clinical Global Impression; COPDD, childhood onset pervasive developmental disorder; COS, Childhood-Onset Schizophrenia; COSS, Childhood-Onset Schizophrenia Spectrum disorders; CS, comparative study; DAS, Psychiatric Disability Assessment Schedule; DepS, Depression Score; DICA-C, Diagnostic Interview for Children and Adolescents- Child Version; DICA-P, Diagnostic Interview for Children and Adolescents-Parent Version; DIGS, Diagnostic Interview for Genetic Studies; DUP, duration of untreated psychosis; EHI, Edinburgh Handedness Inventory; EOS, Early-Onset Schizophrenia; F, female; ids, individuals; FSIQ, full scale intelligence quotient; GAF, Global Functional Assessment Scale; Ha, hallucinations; HC, healthy controls; HM, high mixed; HN, high negative; IQ, Intelligence quotient; LM, low mixed; LS, longitudinal study; K-FTDS, Kiddie Formal Thought Disorder Rating Scale; KSADS, Kiddie Schedule for Affective Disorders and Schizophrenia; K-SADS-E, Schedule for Affective Disorders and Schizophrenia for School-Age Children-Epidemiologic Version; M, male; MDD, major depressive disorder; MDI, Multidimensionally Impaired Children; MISIC, Malin’s Intelligence scale for Indian children; M-PAS, Modified Premorbid Adjustment Scale; NART, National Adult Reading Test; NBT, Nahor Benson test; NIMHGS, National Institute of Mental Health Global Scale; non-COSS, children with other severe non-psychotic psychiatric conditions; NPV, negative predictive value; NVH, No Visual hallucinations group; ODD, oppositional defiant disorder; PANSS, Positive and Negative Syndrome Scale; PAS, Premorbid Adjustment Scale; PDD, pervasive developmental disorder; PPV, positive predictive value; PsyS, Psychosis Score; pts, patients; RS, retrospective study; SANS, Scale for the Assessment Negative Symptoms; SAPS, Scale for the Assessment Positive Symptoms; SCZ, schizophrenia; SIM, Simpson-Angus Extrapyramidal Side Effect Scale; SIRP, Sternberg Item Recognition Paradigm; VEOS, very early onset schizophrenia; VH, visual hallucinations group; WAIS, Wechsler Adult Intelligence Scale; Wh- = what, who, when, why, where; WIS, Wechsler Intelligence Scale; WISC-R, Wechsler Intelligence Scale for Children-Revised; x̄, mean age in years; y, years; ↑, longer/higher/more; ↓, shorter/lower/worse/fewer/less.

### 2.4 Risk of bias assessment

In order to evaluate the reliability of the review and its quality, and to rigorously analyze the outcomes of the chosen studies, a risk of bias analysis was performed. This analysis adhered to the indications and criteria put forth by the Agency for Health Care Research and Quality (37). The online Supplementary material delineate the criteria utilized for assessing the risk of bias. Each study underwent bias assessment in accordance with the stipulated criteria, encompassing selection bias, performance bias, detection bias, attrition bias, and reporting bias. Subsequently, a bias level, categorized as low, medium, or high, was assigned to each study based on the assessment. The included studies were independently evaluated by the authors, and any disparities in the assessments were resolved through discussions. The evaluation of the risk of bias is detailed in the online Supplementary material, specifically in Supplementary Table S2.

## 3 Results

### 3.1 Search results

The aforementioned search yielded an initial collection of 384 articles, spanning publication dates from February 1984 to January 2023. Through the application of inclusion and exclusion criteria, a total of 366 articles were excluded, culminating in a final selection of 18 articles (refer to Table 1). Detailed explanations for the rejection of each study can be found in the Supplementary material, specifically Supplementary Table S1. The complete search results, along with reasons for exclusion when applicable, are depicted in the PRISMA flowchart, accessible in the Supplementary material (Supplementary Figure S1).

### 3.2 Overview of the included studies

The studies exhibit overlapping results across different issues, making it challenging to categorize them distinctly. Nevertheless, we have attempted to identify the primary focus of each study based on the argument that received the most attention. Six of the included studies focused on the risk factors, in particular, sex differences and premorbid neurodevelopment comorbidities before developing psychotic symptoms. Another six studies investigated the main clinical features of VEOS. The remaining six studies underlined the evidence of specific neuropsychological deficits in these patients. Each of these groups of studies is discussed in the following paragraphs.

#### 3.2.1 Risk factors and premorbid neurodevelopmental comorbidities

In Ordóñez et al. (24), a longitudinal study conducted in 2016 on the NIMH cohort, the authors assessed 133 inpatients with COS. The aim of the study was to expand on sex differences in the COS population. The mean age at psychosis onset was 10.29 years for females and 9.51 years for males. This study was conducted on the NIMH cohort, so it shares some characteristics with other NIMH studies. The NIMH study was conducted from 1990 to 2017, and individuals were longitudinally assessed in a period of inpatient hospitalization and observation of up to 3 months. Patient diagnosis of VEOS was re-evaluated after a period of medication wash-out of up to 3 weeks. Similarly to other studies on NIMH cohorts, individuals underwent several tests and interviews, including clinical and anamnestic assessment (CA); Scale for the Assessment Positive (SAPS), and Negative Symptoms (SANS), which are scales used to assess positive and negative symptoms in schizophrenia; Brief Psychiatric Rating Scale (BPRS), a rating scale that is useful for rapidly assessing psychiatric symptoms in patients; Children’s Global Assessment Scale (CGAS), which evaluates the influence of psychiatric symptoms on the subject’s functioning; Kiddie Schedule for Affective Disorders and Schizophrenia (KSADS), a semi-structured interview based on DSM-5 criteria that investigates the occurrence of psychiatric symptoms in adolescent or child subjects; Autism Screening Questionnaire (ASQ), used to assess the presence of Pervasive Developmental Disorder (PDD); different editions of the Wechsler Intelligence Scale for Adults (WAIS), an IQ test designed to measure intelligence and cognitive ability in adults, and the revised version for children (WISC-R), an individually administered intelligence test for children between the ages of 6 and 16, (due to the longitudinal design of the NIMH studies, intelligence tests included adult and children’s versions and included different editions); Simpson-Angus Extrapyramidal Side Effect (SIM), used to evaluate adverse effects from antipsychotic medication; and Abnormal Involuntary Movement Scale (AIMS), used to measure involuntary movements known as tardive dyskinesia. Females had lower verbal IQs than males. Moreover, males displayed younger ages of onset and higher rates of comorbidity with PDD and Attention Deficit Hyperactivity Disorder (ADHD) than females with COS. However, no differences were found between groups in most clinical measures and in premorbid abnormalities across academic, language, and motor domains.

Frazier et al. (30) conducted a retrospective study on a sample of 28 outpatients with VEOS compared to their siblings during puberty. The mean age at psychosis onset was 10.2 years. The sample was assessed by a WISC and Mean Tanner Stage, a scale for measuring physical development as children transition into adolescence and then adulthood. The results showed a significant correlation between the age of onset of secondary sexual characteristics and the age of onset of psychosis, but this correlation was observed only in females. However, the onset of menarche was not related to the onset of psychosis. The study also found that the age of onset of pubertal changes was similar in siblings of the study participants, and there were no significant differences between the age of onset of menarche in females with VEOS and their sisters.

Galitzer et al. (21), aimed to compare clinical characteristics and treatment outcomes of inpatients with Childhood-Onset Schizophrenia Spectrum Disorder (COSS) and those of others with a severe non-psychotic condition (non-COSS). The sample consisted of 20 individuals with a mean age at admission of 11 years, while there were 191 non-COSS patients, with a mean age at admission of 10.7. CGAS and CA were used to assess individuals. COSS showed statistically lower CGAS scores at admission but not on discharge in comparison to non-COSS. Furthermore, COSS were more likely to be on medication with antipsychotics than non-COSS. Females with COSS and older COSS (>11.16 years old) showed a worse profile in terms of CGAS scores, longer duration of admission, and a major presence of medication treatments.

Watkins et al. (36) proposed a retrospective study to describe symptom development from birth to 12 years of age in 18 prepubertal patients (13 inpatients and 5 outpatients), who met DSM-III criteria for VEOS. Data were collected over a 3-year period through the submission of interview protocols: CA, K-SADS, CBCL, WISC-R, and the Children’s Psychiatric Rating Scale (CPRS-D), a clinical tool for obtaining parental reports of childhood behavior problems. The results showed the presence of a history of autism (SA group) in 7 patients and a history of childhood-onset pervasive developmental disorder (COPDD) in 11 patients (S group). The SA group had a significantly earlier onset of schizophrenia than the S group. Girls appeared to have better premorbid histories than boys and a later onset of schizophrenia.

Alaghband-Rad et al. (32) conducted a retrospective study in a sample of 23 outpatients with VEOS. The mean age of onset of psychosis was 12 years or younger. Several tests, questionnaires, and interviews were conducted: Schedule for Affective Disorders and Schizophrenia for School-Age Children in the Epidemiologic Version (K-SADS-E); selected portions of the Diagnostic Interview for Children and Adolescents, Parent Version (DICA-P) and Child Version (DICA-C), for disruptive behavior disorders, substance abuse, and child psychosis; Premorbid Adjustment Scale (PAS), a rating scale that assesses the extent to which developmental goals were met at different times in a person’s life before the onset of schizophrenia; Autism Diagnostic Interview-Revised (ADI-R), a standardized and structured interview for the assessment of children and adolescents suspected of being on the ASD; an unspecified cognitive test or scale developed by Kydd and Werry based on school performance. The results examined quantitative and qualitative abnormalities in the patients that were similar to those in previous reports of very early-onset schizophrenia: 36% of the patients with at least one feature of pervasive developmental disorder, such as autism or transient motor features; 30% with ADHD. The subjects showed low normal IQs, with no consistent cognitive decline, and the most pronounced delays were in language development; furthermore, in males, there were more delays in crawling.

Hollis et al. (33) proposed a retrospective study in a sample of 18 children with VEOS aged between 7 and 13 years and 43 children with EOS aged between 14 and 17 years were compared with 61 healthy controls (HC) matched for age and sex. The sample was recruited from both inpatients and outpatients. The results showed that there were no significant differences in the occurrence of psychotic symptoms. In the VEOS group, the disorder of language production is more common, and onset of symptoms was found to be more insidious. In the EOS group, among the disturbances in motor development, “restlessness and fidgetiness” were more significant.

#### 3.2.2 Clinical features of VEOS

In 2014, Greenstein et al. (8) studied the differences in clinical manifestations between COS and child patients who received a psychiatric alternative diagnosis during the COS differential diagnosis process (AD). The aim was to develop an algorithm through the clinical evaluation of symptoms to better distinguish patients with COS. This study was conducted within the NIMH cohort and shares recruitment and patient assessment characteristics with other NIMH studies. A total of 85 individuals with COS and a mean age at psychosis onset of 9.92 years were compared with 53 individuals with AD and a mean age at psychiatric onset of 8.33 years. Several tests and questionnaires or interviews were conducted: CA, SAPS, SANS, BPRS, CGAS, KSADS, WAIS, WISC, and the National Institute of Mental Health Global Scale (NIMHGS). The latter is a modified Bunney–Hamburg scale (BH) (38) and includes four different scores: psychosis score (PsyS), mania score (ManS), anxiety score (AnxS), and depression score (DepS). Results showed that, in comparison to AD, COS had augmented scores on positive and negative symptoms in SANS, SAPS, BPRS, and NIMHGS PsyS and diminished scores in IQ, CGAS, and NIMHGS DepS and AnxS. Moreover, it emerged that COS had a later age of onset compared to AD. In the multiple logistic regression, the two predictor models including only NIMHGS PsyS and DepS showed a positive predictive value (PPV) of 91.34%, a negative predictive value (NPV) of 55.20%, sensitivity of 78.71%, specificity of 77.56%, and an overall accuracy of 78.42%, with Area Under the Curve (AUC) showing 87.12%. These results indicate that higher psychosis ratings and lower depression ratings combine to increase the probability that a patient has COS respect to another diagnosis.

VEOS individuals show a duration of untreated psychosis (DUP) fourfold longer than EOS and eightfold longer than AOS. These results emerged from a study conducted by Coulon et al. (22) on outpatients with Schizophrenia: 22 with VEOS, 154 with EOS, and 551 with AOS. The mean ages at psychosis onset for the groups were 9.55, 15.9, and 23.7 years, respectively. VEOS were assessed using various tools: CA; the positive and negative syndrome scale (PANSS), a scale used by clinicians for measuring negative and positive symptom severity of patients with schizophrenia, in particular in response to treatments; Calgary Depression Rating Scale for Schizophrenia (CDRS), a measure used to assess the level of depression in people with schizophrenia; Edinburgh Handedness Inventory (EHI), to objectively ascertain the handedness of a subject in activities of daily living; Global Functional Assessment Scale (GAF), which is used to rate the severity of a mental illness; WAIS and National Adult Reading Test (NART), tests that estimate premorbid intelligence. In addition to results related to DUP, authors highlighted that VEOS had higher psychopathological general and total scores on PANSS. Furthermore, VEOS had a lower educational level than EOS and AOS and was associated with a higher presence of learning disabilities compared to AOS. Premorbid IQ did not appear to be altered in VEOS compared to the other groups.

In one longitudinal NIMH study conducted in 2011, David et al. (25) explored the role of hallucinations in COS patients. In particular, 117 inpatients diagnosed with COS were divided into two groups, one with visual hallucinations (VH) (mean age at psychosis onset of 9.7 years) and one with no visual hallucinations (NVH) (mean age at psychosis onset of 10.7 years). The assessment included SAPS, SANS, CGAS, WAIS, and WISC. The results showed a higher prevalence of auditory hallucinations (95%) compared to visual (80.3%), somatic/tactile (60.7%), and olfactory (30%) hallucinations. Furthermore, this study highlights the overlap of hallucination modalities: all individuals with visual hallucinations also experienced auditory hallucinations (but not vice versa) and all individuals with somatic/tactile and olfactory hallucinations also had visual and auditory hallucinations. Finally, VH, in comparison to NVH, had an earlier age of psychosis onset, a younger age at assessment, a lower full-scale IQ, lower CGAS scores, and a shorter duration of illness from the age of first symptom onset. These results report the importance of auditory hallucinations in COS as in AOS but indicate the role of visual hallucinations in the level of illness severity and in association with lower patient IQ.

Cheng et al. (20) conducted a retrospective comparation of 216 individuals affected by COS with 366 individuals with AdOS. Inpatients with COS and AdOS were assessed upon admission to the hospital and at discharge throughout CA and PANSS. COS had a mean age at psychosis onset of 10.6 years, whereas the mean onset age of AdOS was 14.1 years. No differences were found between COS and AdOS in terms of sex, days of hospitalization, psychiatric family history, comorbidity, DUP, and PANSS total score at admission. COS had a lower PANSS positive score at admission and PANSS reduction rate and a higher PANSS negative score at admission and PANSS total score at discharge compared to AdOS. COS more frequently had an insidious onset and a longer illness course. As to clinical features, COS individuals showed more bizarre and impulsive behaviors, visual hallucinations, and formal thought disorder with diminished delusions than AdOS. However, no significant differences were found in the incidence of overall hallucinations, negative symptoms, and early non-specific symptoms between the two groups. Moreover, COS showed less treatment efficacy than AdOS.

In a longitudinal NIMH study, Mattai et al. (27) aimed to examine the relationship between sleep disturbance, clinical severity, and comorbid diagnoses (e.g., anxiety) in a population diagnosed with COS. As a NIMH study, it shares some characteristics in recruitment with other NIMH studies, as previously described. The sample consisted of 61 inpatients with COS, divided into two groups: “good sleepers” (> 6 h, *n* = 30) and “poor sleepers” (< 6 h, *n* = 31) based on the average total hours of sleep per night. The sleep pattern data were collected by measuring safety records and daily nursing notes and examining them in relation to clinical, biological, and genetic markers of COS. Clinical symptoms were assessed using the SAPS; SANS; BPRS; CGAS; Clinical Global Impression (CGI), a measure of symptom severity, treatment response, and treatment efficacy; and BH for depression, mania, psychosis, and anxiety. These assessments were conducted upon admission and weekly during the medication-free period while hospitalized. The results highlighted that “good sleepers” showed better functioning in BPRS, SAPS, and SANS upon admission, and “poor sleepers” showed higher BH-Anxiety scores upon admission. Moreover, “poor sleepers” without any medications had significantly higher scores for SAPS, SANS, and BH-Anxiety and lower scores for CGAS and BPRS. The results supported the correlations between average sleep scores and clinical ratings measured by SAPS and SANS: “poor sleepers” had higher scores in SAPS and SANS upon admission.

In a longitudinal NIMH study, Craddock et al. (23) analyzed the clinical features of 125 inpatients with COS, with a median age of psychosis onset of 9.90 years. The assessment was conducted similarly to other NIMH studies using a CA, along with the following tests: SAPS, SANS, BPRS, CGAS, KSADS, ASQ, WAIS, and WISC. The authors employed confirmatory factor analysis (CFA) and cluster analysis on SAPS and SANS scores. A two-factor solution, including positive and negative symptoms, was found to best fit the COS population. Furthermore, the authors favored a 3-cluster solution after performing K-means cluster analysis using the positive and negative dimensions from the CFA. The three emerging groups were described as follows: low scores on both dimensions (LM), high negative with low positive scores (HN), and high scores on both dimensions (HM). The LM group showed a higher full-scale IQ than HN and HM. The LM group had higher CGAS scores than HM, while HN showed intermediate scores. A trend was observed in the age of onset, with HN being older at onset than LM and HM groups. The LM group showed a stronger trend in comorbidity with behavioral disorders (ADHD, oppositional-defiant disorder, conduct disorder) compared to the HN and HM groups.

#### 3.2.3 Neuropsychological deficits

In a longitudinal study, White et al. (26) attempted to analyze the trajectory of verbal and visuospatial Working Memory (WkM) deficits in COS patients. The sample consisted of 26 inpatients with a diagnosis of COS, and 37 HC, divided into three age groups: 8–11 years, 12–15 years, and 16 years and older. The verbal and visuospatial WkM tasks were evaluated using a modified version of the verbal and visuospatial Sternberg Item Recognition Paradigm (SIRP). The results showed that COS patients performed worse than HC within all three age groups in both verbal and visuospatial modalities. In addition, the trajectory of the verbal SIRP showed a disproportionately lower performance in the COS group (8–12) compared to the older two age groups.

Abu-Akel et al. (29) aimed to characterize the communicative deficits associated with COS. Speech function variables, formal thought disorder, and cohesion were coded in 32 COS inpatients and outpatients, under treatment (15) and not (17), in comparison with 34 HC, aged from 5.6 to 12.4 years. The assessment of speech function was conducted by a videotaped Story Game in which the children answered open-ended standardized questions on each story, and the raters coded the speech function categories from the transcripts of the children’s responses (Yes/No or Wh-questions (what, who, when, why, where)). Formal thought disorder was evaluated from videotapes of the Story Game using the Kiddie Formal Thought Disorder Rating Scale (K-FTDS) and cognitive testing (WISC-R). The analysis of FSIQ revealed inappropriate responses in SCZ subjects, and a correlation of the WISC-R Distractibility factor score with no responses, direct responses, implied responses, and inadequate responses to Yes/No questions in these patients. Regarding the increased use of speech functions, the medicated group had significantly higher scores of no responses and inappropriate responses than the HC. On the contrary, the unmedicated group had significantly more inadequate responses to Yes/No questions compared to the control group. Analyzing the use of speech functions, the medicated group used fewer direct and supplementary responses than the HC, while the unmedicated group used fewer direct responses to Wh-questions. Lastly, when comparing the two groups, the drug-treated group differed from the untreated group only in the higher number of supplementary responses.

Caplan et al. (34) aimed to analyze the thought disorder in a sample of inpatients and outpatients diagnosed with schizophrenia (*n* = 29) and schizotypal (*n* = 10) disorder and 54 healthy children aged 5–12.5 years. Videotapes of 20–25 min story games were independently rated with the K-FTDS by two trained raters who had no previous knowledge of the individual child’s diagnosis. The subject’s total formal thought disorder (FTD) score was the sum of the scores for illogical thinking, loose associations, and poverty of content of speech. The results showed that VEOS and schizotypal subjects had significantly higher scores for illogical thinking and total FTD compared to the control group. A total of 70% of VEOS and 64% of the schizotypal children had loose associations scores above zero. The data among VEOS, schizotypal children, and HC demonstrated that IQ did not affect the diagnostic differences in K-FTDS scores between patients and HC.

In a subsequent study, Caplan et al. (31) conducted research that delved into self-initiated repair in a sample of 32 inpatients and outpatients with VEOS (18 medicated and 14 unmedicated) and in 47 HC, along with the correlation with cohesion of language, distractibility, and clinical measures of FTD. The mean age of patients with VEOS was 10.3 years. Several tests and questionnaires or interviews were conducted: Interview for Childhood Disorders and Schizophrenia, a diagnostic interview for schizophrenia; Story Game, a test in which the child listens to two recorded stories, then retells them and answers standardized open-ended questions about the stories, the child also has to invent a tale selected from various suggested topics; K-FTDS; and WISC-R. The results showed a diagnostic effect for the following cohesive variables: referential cohesion, conjunctions, unclear/ambiguous reference, and verbal productivity (words per clause). Within the VEOS group, the WISC-R Distractibility factor score was found to be significantly related to false starts, repetitions, and loose associations, and higher scores for illogical thinking were reported. The medicated VEOS used less referential cohesion, referential revision, and word choice revision and fewer conjunctions, words per clause, false starts, and fillers than the controls and less referential revision and postponement and fewer fillers than the patients without medication. VEOS with loose associations used more false starts and fillers than those without loose associations.

The goal of Caplan et al. study in 1990 (35) was to examine whether loose associations represent a clinical manifestation of impaired attention/information processing and global cognitive deficits in children with schizophrenia. The authors conducted a longitudinal study with a sample of 31 inpatients (70%) and outpatients with VEOS, with an average age of 10.2 years. They assessed patients using K-FTDS, WISC-R, and the Span of Apprehension, which provides an index of the rate of visual information processing. The results showed no significant correlation between illogical thinking and loose associations. Loose associations were negatively and significantly correlated with FSIQ and the WISC-R distractibility factor but not with the verbal IQ and performance IQ subscores. After partializing out the variance from the distractibility factor scores, loose associations were not significantly correlated with FSIQ and performance IQ illogical thinking was not significantly associated with FSIQ verbal IQ, performance IQ, or the distractibility factor scores. VEOS individuals with a partial span of apprehension scores had higher illogical thinking scores.

Biswas et al. (28) aimed to test the hypothesis that the earlier the onset, the greater the severity of illness and neuropsychological deficits. The authors conducted a comparison of the neuropsychological profiles of 15 outpatients affected by COS, with 20 individuals with AdOS and 20 AOS patients. An assessment of neuropsychological profile was carried out using the Malin’s Intelligence Scale for Indian Children (MISIC), which is an Indian adaptation of the Wechsler Intelligence Scale for Children; Memory scale (PGI) was used to assess memory functioning; perceptuomotor skills were assessed using the Nahor Benson Test (NBT) and the Bender Visual Motor Gestalt Test (BVMG); and clinical symptoms were assessed with PANSS. The results showed that the COS group had significantly higher PANSS Positive and Negative scores and PANSS General Psychopathology scores than AdOS and AOS. The COS group performed poorly on all the subtests of memory PGI, except for recent memory. The authors had higher error scores and dysfunction rating scores in BVMG and NBT.

## 4 Discussion

VEOS has characteristics that are distinct from other psychiatric disorders and other forms of schizophrenia (i.e., EOS, AOS). To draw out these distinctions, in this section, we will compare the results of our review with the literature on EOS and AOS.

The first notable difference between these conditions is that, while EOS and AOS tend to exhibit varying frequency rates among sexes, no sex differences have been found with respect to the frequency of VEOS. A study conducted over a 15-year period on the entire English population by Seminog et al. (14) revealed that sex differences in schizophrenia only emerge at around 14 years of age, revealing a progressively higher incidence in males compared to females. In contrast, in adulthood, schizophrenia is more frequent in males, although the prevalence of the disorder has shown minor differences, leading to some controversies in the field (14, 39–43). An intriguing finding that emerged from our review is that, in females, VEOS onset appears to be associated with the emergence of secondary sexual characteristics, and no significant timing differences have been observed concerning menarche between VEOS and healthy subjects (30).

Sex differences in schizophrenia may also refer to clinical features. In this respect, Abel et al. (40) suggested that females tend to exhibit a milder form of AOS. Additionally, other studies have found that females are more likely to demonstrate a later onset of psychosis, a better response to drug therapy, and fewer negative symptoms (44, 45). However, these observations only partially align with evidence collected from VEOS samples. Males with VEOS consistently displayed an earlier onset of symptoms, while females tended to have poorer functional outcomes (as indicated by CGAS scores), longer periods of hospitalization, and greater use of medication (21). However, Ordonez et al. reported no differences in clinical characteristics between sexes (24). Therefore, susceptibility to VEOS appears to be influenced by the onset of puberty and sexual differentiation during adolescence, with female hormones—particularly estrogens—possibly serving as protective factors (46). As a result, females with AOS might exhibit a more favorable clinical profile because, after puberty, estrogen may contribute to symptom improvement. Indeed, in the case of VEOS, females tend to exhibit more severe symptoms, which contrasts with the findings for AOS. Conversely, males with any of the three schizophrenia subtypes (i.e., AOS, EOS, and VEOS) tend to experience earlier symptom onset and increased comorbidities with neurodevelopmental disorders. Among VEOS patients, males tend to show greater comorbidities with PDD and ADHD (47). Similar comorbidities have also been observed among males in the EOS population (43). This aligns with findings that VEOS patients with a history of autism may experience earlier symptom onset (36). However, no sex differences have been found in premorbid levels of academic, motor, and language performance (24).

Regardless of sex differences, VEOS is often associated with high comorbidity rates of approximately 30%, for both PDD and ADHD. Individuals with VEOS also tend to experience neurodevelopmental difficulties, primarily in language and school skills (22, 32, 33). This aligns with results pointing out that VEOS patients exhibited more impulsive and bizarre behaviors compared to patients with AOS (20). A study focusing on childhood and early adolescent patients with VEOS and schizoaffective disorder confirmed the high comorbidity with ADHD (48). Additionally, the NIMH cohort study revealed a high percentage of ASD (approximately 20%) and non-specific neurodevelopmental impairments (e.g., in motor, language, and social skills) in individuals with VEOS (7, 47). In the adult population, there appears to be a correlation between ASD and schizophrenia, in terms of both shared clinical characteristics (e.g., social communication deficits and reduced emotional expression) (49) and high comorbidity (50, 51). Genetic studies have provided further support for the correlation between schizophrenia and neurodevelopmental disorders, demonstrating a link between autism and schizophrenia (11, 52), as well as between autism, schizophrenia, and ADHD (13, 53). However, it is important to note that imaging studies have indicated distinct brain changes in autism and schizophrenia, suggesting that the correlation between these two disorders may not be straightforward (47). Regarding intelligence, a study found no evidence of impaired premorbid IQ in VEOS patients, compared to those with EOS and AOS (22). However, this finding may have been influenced by the NIMH criteria, which set a minimum cut-off of 70 for premorbid IQ in VEOS patients. This criterion is controversial, given observations that AOS patients show a lower lifelong IQ, and any IQ reduction tends to occur mostly in the months preceding psychotic onset, before stabilizing for the remainder of the illness (54).

Compared to AOS and EOS, VEOS exhibits some distinct characteristics in illness onset. Notably, VEOS typically presents with a more insidious onset and follows a longer course compared to EOS (20). Additionally, studies have highlighted a low percentage of acute onset of psychosis in VEOS (55, 56). Moreover, the DUP in VEOS is generally longer than that of both EOS and AOS (22). However, one study found similarity in the DUP of VEOS and EOS (20). Generally, patients with schizophrenia who experience an earlier onset tend to have longer DUP (57, 58). According to Coulon et al. (22), the DUP of VEOS is four times longer (approximately 8 years) than that of EOS (1.8 years) and eight times longer than that of AOS (1 year). These findings align with the study of Stentebjerg-Olesen et al. (51), which identified that the DUP of EOS is 3.5 times longer than that of AOS. This insidious onset and prolonged DUP in VEOS may contribute to delayed recognition of the disorder and, consequently, a worse prognosis, given that prolonged DUP is associated with poorer outcomes (59). VEOS appears to be more clinically severe than EOS and AOS. In more detail, VEOS patients score higher overall on the PANSS compared to EOS and AOS patients (22). Furthermore, among VEOS patients (compared to EOS and AOS patients), higher PANSS scores have been shown to be associated with greater impairment in neuropsychological aspects such as memory and visuomotor abilities (28). However, a study by Cheng et al. (20) found no significant differences in PANSS scores at ward admission between diagnostic groups, with the exception of higher scores for negative symptoms among VEOS patients and higher PANSS scores among VEOS patients at discharge, suggesting a lower treatment response of VEOS patients (20). Additionally, the greater severity of VEOS is evident in the early brain alterations observed in neuroimaging studies, which align with the alterations observed in AOS but exhibit increased severity. Specifically, in VEOS, gray matter alterations show increased volume loss, progressing from posterior to anterior regions (i.e., parieto-fronto-temporal) and persisting throughout adolescence, with normalization occurring at around the age of 20 years (7, 12). These early alterations are associated with slower white matter growth and decreased cerebellum and insula volume (7, 12), indicating greater disrupted neurodevelopment in VEOS and suggesting a neurodevelopmental etiology for AOS, as well (12). Furthermore, studies have observed a tendency for VEOS patients to demonstrate greater resistance to pharmacological treatment (6) and a higher familial presence of schizophrenia, compared to AOS patients (11). Moreover, it is widely accepted that treatment for VEOS tends to be initiated later and is less effective (6).

From a clinical perspective, VEOS appears to exhibit distinct features in negative and positive psychotic symptoms. An interesting correlation has been found between the presence of these symptoms and IQ, with more psychotic symptoms associated with lower IQs and worse global functioning (23). Concerning negative symptoms, Craddock et al. (23) concluded that, among VEOS patients, negative symptoms may play a crucial role and exhibit a strong association with poor clinical manifestations, as, in their study, the group with only higher negative symptoms (HN group) exhibited intermediate scores relative to the other groups. This suggested a more relevant role of negative symptoms compared to positive symptoms. The centrality of negative symptoms in schizophrenia and the correlation between negative symptoms and disorder severity has also been recognized in AOS (60–62). However, the combination of both positive and negative symptoms appears to be a typical feature of VEOS and is therefore useful for distinguishing VEOS from other psychiatric diagnoses in childhood (8). Of note, depressive symptoms have been found to be more associated with non-VEOS diagnoses, further underscoring the distinction between negative and depressive symptoms in this disorder. Pontillo et al. (59) emphasized that the clinical presentations of VEOS and EOS may vary depending on the presence or absence of comorbid neurodevelopmental disorders. In this vein, one study found associations between neurodevelopmental disorders and more positive and disorganized symptoms, as well as between a lack of neurodevelopmental disorders and more negative symptoms (63). While different clinical profiles may emerge depending on comorbidities with neurodevelopmental disorders, negative symptoms appear pivotal for defining schizophrenic symptomatology. Nonetheless, a better characterization of the negative dimension of VEOS is needed, as knowledge of the precise manifestation of different negative symptoms (e.g., blunted affect, social withdrawal, and anhedonia) is currently lacking.

Regarding positive symptoms, VEOS is strongly correlated with the presence of auditory hallucinations—similar to what has been observed in AOS samples (64). Such hallucinations seem to play a central role in the disorder (25), and they have been found to be associated with increased risk of psychotic onset (65, 66). Compared to patients with AOS and EOS, VEOS patients demonstrate a higher frequency of visual hallucinations (20, 25). However, auditory hallucinations remain fundamental to the disorder. Indeed, in VEOS, different types of hallucination can overlap, but auditory hallucinations are consistently reported, and visual and olfactory/somatic hallucinations are experienced only in association with auditory hallucinations. Nevertheless, visual hallucinations, in particular, appear to be an indicator of severity associated with lower IQ (23), poorer functioning, and earlier symptom onset (25). In schizophrenia, hallucinations take on greater significance when they are accompanied by other symptoms, such as thought disorders (67, 68). This is particularly relevant to VEOS, as the disorder is characterized by a poverty of language content, illogicality, and loss of association (31, 35). This partly corresponds to the observation that VEOS exhibits both more formal thought alterations and more hallucinations (20, 25). Thought disorders, which alter the perception of one’s internal dialog, appear to be associated with the development of auditory hallucinations (69, 70) and may relate to the neuropsychological changes observed in schizophrenia patients. Similarly, greater hallucination in AOS patients has been shown to correlate with lower cognitive performance (71). Impaired executive functioning relating to working memory and attention has also been shown to be a consistent feature of schizophrenia (54), as evidenced by functional neuroimaging studies (72, 73). More specifically, both AOS and VEOS patients tend to show impairments in linguistic and visuospatial working memory (26). Mixed evidence has emerged regarding the association of greater impairment of working memory and auditory hallucinations (74, 75).

Further research is needed to investigate the frequency and characteristics of delusions in VEOS. To date, only a few studies have explored this topic, observing the frequency of symptom presentation in a clinical group without conducting a statistical analysis or establishing a direct correlation with AOS. Nevertheless, the findings of these studies suggest that delusions may be prevalent in the majority of patients with VEOS (55, 56, 76). Notably, Russell (55) observed a lower complexity in delusions, with a greater presence of infantile themes and a possible lower frequency overall, in VEOS patients compared to AOS patients. Only one study included found a lower frequency of delusions in VEOS patients relative to EOS patients (20). No comparisons of delusions between VEOS and AOS patients were drawn in the included studies.

Furthermore, brain imaging studies have demonstrated differences between VEOS patients and children with similar symptoms but a non-schizophrenia diagnosis (7, 12). From a clinical perspective, VEOS appears to be distinct from other disorders with similar presentations by virtue of the higher prevalence of both positive and negative psychotic symptoms, compared to depressive symptoms (8). VEOS also exhibits a later onset compared to other psychiatric conditions in childhood (20). These findings help to distinguish VEOS from psychiatric conditions with similar symptoms, such as mood disorders and multidimensional impairment (77). Therefore, VEOS stands apart from not only other forms of schizophrenia but also from other childhood psychiatric disorders.

The findings of this review, when compared to the existing scientific literature on EOS and AOS, shed light on VEOS while leaving numerous questions unanswered. Schizophrenia typically arises following significant alterations of central nervous system (CNS) maturation, such as during early neurodevelopment (e.g., synaptogenesis) and adolescence (e.g., synaptic pruning) (78–80). In particular, synaptic pruning is associated with dysregulation of the CNS excitation and inhibition systems and dysregulation of the dopaminergic and glutamatergic networks, which may contribute to schizophrenic symptoms (78). VEOS onset precedes adolescent neurodevelopment, suggesting the presence of pathogenetic differences compared to AOS. However, many clinical features and fundamental brain changes exhibit a high degree of continuity between VEOS and AOS. It may be that schizophrenia, as a multifactorial disorder, involves various etiological and pathogenetic elements (e.g., genetic susceptibility, traumatic life events, altered synaptogenesis, altered synaptic pruning, and neurotransmitter network dysregulation), of which only a subset applies to VEOS. Notably, VEOS suggests that earlier factors (e.g., genetic components and early brain development) may play a greater role, as supported by the comorbidities with neurodevelopmental disorders. While the impact of traumatic life events in VEOS may appear less evident, such events may still occur very early on, even during gestation and birth (81).

Furthermore, since synaptic pruning occurs at a later age, the brain alterations observed in VEOS must be linked to either premature synaptic pruning or other early changes in brain processes, leading to a structural and functional similarity with AOS. However, this theory must be subjected to further research investigating the structural and functional brain diversity in VEOS with respect to AOS. The variations in pathogenesis observed in VEOS may account for the epidemiological and clinical specificity. As discussed, onset at prepuberal age also appears to affect the course of the disorder, especially among female subjects, who cannot yet benefit from the possible protective role played by estrogens. The lower response to drug therapies and higher frequency of visual hallucinations in VEOS may account for the distinct neurotransmission alterations, compared to AOS, possibly involving dysregulation in the dopamine network. On the other hand, VEOS and AOS have some common clinical elements (e.g., negative symptomatology and the importance of auditory hallucinations), suggesting shared pathogenetic processes. The presence of a prodromal phase in VEOS is yet to be clarified. While the long DUP and insidious onset associated with VEOS suggest its presence, this topic remains underexplored.

## 5 Limitations and strengths

The present review has both limitations and strengths, which should be underlined. First, the number of available studies was relatively limited, and the investigated studies applied a variety of population selection criteria. Additionally, some topics were more extensively researched than others. For example, a lack of research on delusions in VEOS and the qualitative characteristics of positive and negative symptoms in this population was evident. Moreover, some studies (e.g., the NIMH studies) employed more rigorous selection methods. VEOS is an extremely rare and challenging condition to recognize, and this may have led to recruitment errors in the absence of a strict and well-structured selection process. Another potential weakness is the heterogeneity in sample sizes and evaluation methods across the studies, including variations in inpatient and outpatient assessments. Similarly, variation in the drug therapies among the study populations may represent a confounding factor. For example, the NIMH studies evaluated patients after a pharmacological wash-out period, while other studies assessed patients with a regular medication regimen. Finally, the present review only consulted a single database (PubMed/MEDLINE) to retrieve relevant studies. This can potentially affect the quality of a systematic review, as emphasized by other authors (82).

On the other hand, the present study represents one of the few attempts to systematically review and summarize the literature on the clinical and neuropsychological features of VEOS. Thus, this review contributes to the existing knowledge by providing a comprehensive overview of current research.

## 6 Conclusion

In conclusion, VEOS appears to be continuous with EOS and AOS while also exhibiting distinct and recognizable characteristics. An absence of sex differences in frequency, the worse clinical profile demonstrated by females, increased severity and treatment resistance, higher presence of visual hallucinations, and comorbidities with neurodevelopmental disorders appear to be specific to VEOS. At the same time, VEOS seems to share with AOS and EOS the importance of auditory hallucinations and negative symptoms as clinical features, specific brain alterations, genetic risk factors, and some comorbidities with neurodevelopmental disorders (e.g., autism spectrum disorder).

While interest in VEOS has been growing in recent years, the literature examining the clinical characteristics of this disorder remains limited. Further research is needed to establish a definitive VEOS profile. The present study aimed to raise awareness of this rare condition, providing support for clinicians’ efforts to establish an early diagnosis and develop effective treatment plans for children with psychosis.

## Data availability statement

The original contributions presented in the study are included in the article/Supplementary material, further inquiries can be directed to the corresponding author/s.

## Author contributions

ML: Writing – original draft, Writing – review & editing. MP: Writing – review & editing. MV: Writing – original draft. AA: Writing – original draft. DB: Writing – original draft. CV: Writing – original draft. SV: Writing – review & editing.
