# Supplementary material for: Clinical features and comorbidity in very early-onset schizophrenia: a systematic review
Source: Front Psychiatry. 2023 Dec 13;14:1270799. doi: 10.3389/fpsyt.2023.1270799 (PMC10752227; doi:10.3389/fpsyt.2023.1270799)
Supplement: Supplementary file 1 [file Data_Sheet_1.docx]

***Supplementary Materials***

**Supplementary Figure 1.** PRISMA flowchart.

**Search strategy. *PubMed:*** “very early onset schizophrenia” OR “childhood onset schizophrenia”

***ClinicalTrials.gov***: “very early onset schizophrenia” OR “childhood onset schizophrenia”

*on 28-02-2023*

**Identification of studies via databases and registers**

Records identified from*:

Databases (n = 384, PubMed)

Other sources (n = 0)

Registers (n = 0, ClinicalTrials.gov)

Records removed *before screening*:

Duplicate records removed (n = 0)

Records marked as ineligible by automation tools (n = 0)

Records removed for other reasons (n = 0)

**Identification**

Records excluded** (n = 325)

No Clinical (n=187)

Case Report (n=33)

Review (n=58)

No VEOS (n=28)

Unrelated (n=10)

No English (n=5)

Letter (n=2)

Lumping (n=2)

Records screened

(n = 384)

Reports sought for retrieval

(n = 384)

**Screening**

Reports not retrieved

(n = 0)

Records excluded (n = 41 )

No Clinical (n= 2)

Review (n=5)

No VEOS (n=9)

No English (n=3)

Letter (n=1)

Lumping (n=13)

No Data (n=8)

Reports assessed for eligibility

(n = 59)

Studies included in review

(n =18)

Reports of included studies

(n = 18)

**Included**

*Consider, if feasible to do so, reporting the number of records identified from each database or register searched (rather than the total number across all databases/registers).

**If automation tools were used, indicate how many records were excluded by a human and how many were excluded by automation tools. *No automation tools were used*

*From:* Page MJ, McKenzie JE, Bossuyt PM, Boutron I, Hoffmann TC, Mulrow CD, et al. The PRISMA 2020 statement: an updated guideline for reporting systematic reviews. BMJ 2021;372:n71. doi: 10.1136/bmj.n71

**Supplementary Figure 2.** PRISMA Checklist.


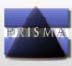


**PRISMA 2020 Checklist**

| **Section and Topic** | **Item #** | **Checklist item** | **Location where item is reported** |
| --- | --- | --- | --- |
| **TITLE** | | |  |
| Title | 1 | Identify the report as a systematic review. | Title |
| **ABSTRACT** | | |  |
| Abstract | 2 | See the PRISMA 2020 for Abstracts checklist. | Abstract |
| **INTRODUCTION** | | |  |
| Rationale | 3 | Describe the rationale for the review in the context of existing knowledge. | Introduction |
| Objectives | 4 | Provide an explicit statement of the objective(s) or question(s) the review addresses. | Introduction |
| **METHODS** | | |  |
| Eligibility criteria | 5 | Specify the inclusion and exclusion criteria for the review and how studies were grouped for the syntheses. | Methods |
| Information sources | 6 | Specify all databases, registers, websites, organisations, reference lists and other sources searched or consulted to identify studies. Specify the date when each source was last searched or consulted. | Methods |
| Search strategy | 7 | Present the full search strategies for all databases, registers and websites, including any filters and limits used. | Methods |
| Selection process | 8 | Specify the methods used to decide whether a study met the inclusion criteria of the review, including how many reviewers screened each record and each report retrieved, whether they worked independently, and if applicable, details of automation tools used in the process. | Methods |
| Data collection process | 9 | Specify the methods used to collect data from reports, including how many reviewers collected data from each report, whether they worked independently, any processes for obtaining or confirming data from study investigators, and if applicable, details of automation tools used in the process. | Methods  Acknowledgements |
| Data items | 10a | List and define all outcomes for which data were sought. Specify whether all results that were compatible with each outcome domain in each study were sought (e.g. for all measures, time points, analyses), and if not, the methods used to decide which results to collect. | Methods  Supplements |
|  | 10b | List and define all other variables for which data were sought (e.g. participant and intervention characteristics, funding sources). Describe any assumptions made about any missing or unclear information. | Supplements |
| Study risk of bias assessment | 11 | Specify the methods used to assess risk of bias in the included studies, including details of the tool(s) used, how many reviewers assessed each study and whether they worked independently, and if applicable, details of automation tools used in the process. | Methods  Supplements |
| Effect measures | 12 | Specify for each outcome the effect measure(s) (e.g. risk ratio, mean difference) used in the synthesis or presentation of results. | N/A |
| Synthesis methods | 13a | Describe the processes used to decide which studies were eligible for each synthesis (e.g. tabulating the study intervention characteristics and comparing against the planned groups for each synthesis (item #5)). | Methods |
|  | 13b | Describe any methods required to prepare the data for presentation or synthesis, such as handling of missing summary statistics, or data conversions. | Methods |
|  | 13c | Describe any methods used to tabulate or visually display results of individual studies and syntheses. | Methods |
|  | 13d | Describe any methods used to synthesize results and provide a rationale for the choice(s). If meta-analysis was performed, describe the model(s), method(s) to identify the presence and extent of statistical heterogeneity, and software package(s) used. | Methods |
|  | 13e | Describe any methods used to explore possible causes of heterogeneity among study results (e.g. subgroup analysis, meta-regression). | N/A |
|  | 13f | Describe any sensitivity analyses conducted to assess robustness of the synthesized results. | N/A |
| Reporting bias assessment | 14 | Describe any methods used to assess risk of bias due to missing results in a synthesis (arising from reporting biases). | Methods  Supplements |
| Certainty assessment | 15 | Describe any methods used to assess certainty (or confidence) in the body of evidence for an outcome. | Methods |
| **RESULTS** | | |  |
| Study selection | 16a | Describe the results of the search and selection process, from the number of records identified in the search to the number of studies included in the review, ideally using a flow diagram. | Results  Supplements |
|  | 16b | Cite studies that might appear to meet the inclusion criteria, but which were excluded, and explain why they were excluded. | Supplements |
| Study characteristics | 17 | Cite each included study and present its characteristics. | Results |
| Risk of bias in studies | 18 | Present assessments of risk of bias for each included study. | Supplements  Limitations and strengths |
| Results of individual studies | 19 | For all outcomes, present, for each study: (a) summary statistics for each group (where appropriate) and (b) an effect estimate and its precision (e.g. confidence/credible interval), ideally using structured tables or plots. | Table 1  Results |
| Results of syntheses | 20a | For each synthesis, briefly summarise the characteristics and risk of bias among contributing studies. | Results  Supplements |
|  | 20b | Present results of all statistical syntheses conducted. If meta-analysis was done, present for each the summary estimate and its precision (e.g. confidence/credible interval) and measures of statistical heterogeneity. If comparing groups, describe the direction of the effect. | N/A |
|  | 20c | Present results of all investigations of possible causes of heterogeneity among study results. | Discussion  Limitations and strengths |
|  | 20d | Present results of all sensitivity analyses conducted to assess the robustness of the synthesized results. | N/A |
| Reporting biases | 21 | Present assessments of risk of bias due to missing results (arising from reporting biases) for each synthesis assessed. | Supplements  Limitations and strengths |
| Certainty of evidence | 22 | Present assessments of certainty (or confidence) in the body of evidence for each outcome assessed. | Supplements  Results |
| **DISCUSSION** | | |  |
| Discussion | 23a | Provide a general interpretation of the results in the context of other evidence. | Discussion |
|  | 23b | Discuss any limitations of the evidence included in the review. | Limitations and strengths |
|  | 23c | Discuss any limitations of the review processes used. | Limitations and strengths |
|  | 23d | Discuss implications of the results for practice, policy, and future research. | Conclusions |
| **OTHER INFORMATION** | | |  |
| Registration and protocol | 24a | Provide registration information for the review, including register name and registration number, or state that the review was not registered. | N/A |
|  | 24b | Indicate where the review protocol can be accessed, or state that a protocol was not prepared. | N/A |
|  | 24c | Describe and explain any amendments to information provided at registration or in the protocol. | N/A |
| Support | 25 | Describe sources of financial or non-financial support for the review, and the role of the funders or sponsors in the review. | Funding |
| Competing interests | 26 | Declare any competing interests of review authors. | Disclosure statement |
| Availability of data, code and other materials | 27 | Report which of the following are publicly available and where they can be found: template data collection forms; data extracted from included studies; data used for all analyses; analytic code; any other materials used in the review. | Supplements |

*From:* Page MJ, McKenzie JE, Bossuyt PM, Boutron I, Hoffmann TC, Mulrow CD, et al. The PRISMA 2020 statement: an updated guideline for reporting systematic reviews. BMJ 2021;372:n71. doi: 10.1136/bmj.n71

For more information, visit: <http://www.prisma-statement.org/>

**Supplementary Table 1.** Table of Included/excluded studies

| 1 | Schizophr Bull. 2023 Jan 31:sbac200. doi: 10.1093/schbul/sbac200. Online ahead of print. The Triad of Childhood-Onset Schizophrenia, Autism Spectrum Disorder, and Catatonia: A Case Report. Leslie AC , O'Sullivan M. | Case Report |
| --- | --- | --- |
| 2. | Schizophr Res. 2023 Jan 14;252:138-145. doi: 10.1016/j.schres.2022.12.033. Online ahead of print. Genetic insights into childhood-onset schizophrenia: The yield of clinical exome sequencing. Alkelai A , Greenbaum L , Shohat S , Povysil G , Malakar A , Ren Z , Motelow JE , Schechter T , Draiman B , Chitrit-Raveh E , Hughes D , Jobanputra V , Shifman S , Goldstein DB , Kohn Y . | No Clinical |
| 3. | J Pers Med. 2022 Oct 31;12 :1796. doi: 10.3390/jpm12111796. Ribosomal DNA Abundance in the Patient's Genome as a Feasible Marker in Differential Diagnostics of Autism and Childhood-Onset Schizophrenia. Ershova ES , Veiko NN , Nikitina SG , Balakireva EE , Martynov AV , Chudakova JM , Shmarina GV , Kostyuk SE , Salimova NA , Veiko RV , Porokhovnik LN , Asanov AY , Izhevskaia VL , Kutsev SI , Simashkova NV , Kostyuk SV . | No Clinical |
| 4. | J Am Acad Child Adolesc Psychiatry. 2022 Nov 2:S0890-8567(22)01906-2. doi: 10.1016/j.jaac.2022.07.850. Online ahead of print. Morbidity Due to Disparity in Pediatric Electroconvulsive Therapy. Ong M , Patterson E , Stewart L , Pierce D , Smith JR . | Letter |
| 5. | Medicine (Baltimore). 2022 Aug 26;101(34):e30288. doi: 10.1097/MD.0000000000030288. Diagnosis stability and outcome of psychotic episodes in a Romanian group of children and adolescents. Rad F , Stancu M , Andrei LE , Linca FI , Mariana Buică A , Leti MM , Dobrescu I , Mihailescu I , Efrim-Budisteanu M . | Lumping |
| 6. | Medicine (Baltimore). 2022 Aug 5;101(31):e29413. doi: 10.1097/MD.0000000000029413. Alternating hemiplegia of childhood: a distinct clinical entity and ATP1A3-related disorders: A narrative review. Pavone P , Pappalardo XG , Ruggieri M , Falsaperla R , Parano E . | Review |
| 7. | Front Aging Neurosci. 2022 Jun 16;14:829217. doi: 10.3389/fnagi.2022.829217. eCollection 2022. Exploration of Mutated Genes and Prediction of Potential Biomarkers for Childhood-Onset Schizophrenia Using an Integrated Bioinformatic Analysis. He F , Zhou YM , Qi YJ , Huang HH , Guan L , Luo J , Cheng YH , Zheng Y . | No Clinical |
| 8. | Cureus. 2022 Feb 25;14 :e22594. doi: 10.7759/cureus.22594. eCollection 2022 Feb.Differentiating Childhood-Onset Schizophrenia From Other Childhood Disorders.Adhikari S , Ghane N , Ascencio M , Abrego T , Aedma K . | Case Report |
| 9. | Neurol India. 2022 Jan-Feb;70 :375-376. doi: 10.4103/0028-3886.338699. Presentation of Mucopolysaccharidosis As Very Early Onset Schizophrenia Like Illness in Psychiatry Settings. Hatila S , Solanki G . | No Clinical |
| 10. | J Child Adolesc Psychopharmacol. 2022 Feb;32 :2-11. doi: 10.1089/cap.2021.0092. Epub 2022 Jan 28. Clozapine for Management of Childhood and Adolescent-Onset Schizophrenia: A Systematic Review and Meta-Analysis. Adnan M , Motiwala F , Trivedi C , Sultana T , Mansuri Z , Jain S . | Review |
| 11. | Zh Nevrol Psikhiatr Im S S Korsakova. 2021;121(11. Vyp. 2):26-30. doi: 10.17116/jnevro202112111226. [A clinical case of continuous schizophrenia with onset in early childhood]. Nikitina SG . | Case Report |
| 12. | Front Psychiatry. 2021 Sep 16;12:662093. doi: 10.3389/fpsyt.2021.662093. eCollection 2021. Neurodevelopmental Trajectories and Clinical Profiles in a Sample of Children and Adolescents With Early- and Very-Early-Onset Schizophrenia. Pontillo M , Averna R , Tata MC , Chieppa F , Pucciarini ML , Vicari S . | Lumping |
| 13. | Child Psychiatry Hum Dev. 2023 Feb;54 :241-247. doi: 10.1007/s10578-021-01245-z. Epub 2021 Sep 15. Impact on the Risk and Severity of Childhood Onset Schizophrenia of Schizophrenia Risk Genetic Variants at the DRD2 and ZNF804A Loci. Alfimova MV , Nikitina SG , Lezheiko TV , Simashkova NV , Golimbet VE . | No Clinical |
| 14. | Front Psychiatry. 2021 Jul 30;12:679807. doi: 10.3389/fpsyt.2021.679807. eCollection 2021. Long-Term Outcomes and Predictors of Childhood-Onset Schizophrenia: A Naturalistic Study of 6-year Follow-Up in China. Liangrong Z , Guican Z , Qi Z , Weirui Y , Yaqi Z , Tong L , Wenjing L , Ming Z , Nianhong G . | No VEOS |
| 15. | J Child Adolesc Psychopharmacol. 2021 Sep;31 :514-515. doi: 10.1089/cap.2021.0048. Epub 2021 Jun 24.Letter to the Editor: Rapid Clozapine Concentration Increase Following Augmentation with Low-Dose Fluvoxamine in 12-Year-Old with Childhood Onset Schizophrenia. Boyle K , Cornett S , Fijtman A , Hunt-Harrison T . | Letter |
| 16. | Eur Child Adolesc Psychiatry. 2022 Dec;31 :1943-1951. doi: 10.1007/s00787-021-01817-3. Epub 2021 Jun 18. National record-linkage study of hospital admissions for schizophrenia in childhood and adolescence in England. Seminog O , Hoang U , Goldacre M , James A . | No Clinical |
| 17. | Am J Med Genet A. 2021 Jun;185 :1841-1847. doi: 10.1002/ajmg.a.62155. Epub 2021 Mar 15. A novel microduplication in INPP5A segregates with schizophrenia spectrum disorder in the family of a patient with both childhood onset schizophrenia and autism spectrum disorder. Fernandez A , Drozd M , Thümmler S , Bardoni B , Askenazy F , Capovilla M . | No Clinical |
| 18. | Can J Psychiatry. 2021 Dec;66 :1042-1050. doi: 10.1177/0706743721990822. Epub 2021 Feb 10. [Neurodevelopmental Disorders, Psychiatric Comorbidities and Associated Pathologies in Patients with Childhood-Onset Schizophrenia and Premorbid Autistic Symptoms.]. Fernandez A , Pasquet-Levy M , Laure G , Thümmler S , Askenazy F . | No English |
| 19. | Cureus. 2020 Dec 14;12 :e12076. doi: 10.7759/cureus.12076. Lack of Resources for Aftercare for Children With Psychosis: A System's Failure. Shirk D , Horn S , Williams SD , Lagman JG . | Case Report |
| **20.** | **Early Interv Psychiatry. 2021 Dec;15 :1721-1729. doi: 10.1111/eip.13121. Epub 2021 Jan 19. Comparison of clinical characteristics and treatment efficacy in childhood-onset schizophrenia and adolescent-onset schizophrenia in mainland China: A retrospective study. Cheng X , Zhang H , Zhang J , Xu P , Jin P , Fang H , Chu K , Ke X .** | **Included** |
| 21. | Exp Ther Med. 2020 Dec;20 :210. doi: 10.3892/etm.2020.9340. Epub 2020 Oct 14. Predictive factors in early onset schizophrenia. Budisteanu M , Andrei E , Linca F , Hulea DS , Velicu AC , Mihailescu I , Riga S , Arghir A , Papuc SM , Sirbu CA , Mitrica M , Docu-Axelerad A , Ghinescu MC , Dobrescu I , Rad F . | No VEOS |
| 22. | Turk Pediatri Ars. 2020 Sep 23;55 :222-228. doi: 10.14744/TurkPediatriArs.2020.65693. eCollection 2020.Whatever happened to multiple complex developmental disorder? Posar A , Visconti P . | No Clinical |
| 23. | Schizophr Res. 2020 Sep;223:327-336. doi: 10.1016/j.schres.2020.08.022. Epub 2020 Sep 24. Sleep spindle activity in childhood onset schizophrenia: Diminished and associated with clinical symptoms. Markovic A , Buckley A , Driver DI , Dillard-Broadnax D , Gochman PA , Hoedlmoser K , Rapoport JL , Tarokh L . | No Clinical |
| 24. | J Hum Genet. 2021 Mar;66 :339-343. doi: 10.1038/s10038-020-00846-1. Epub 2020 Sep 18.Expansion of the GRIA2 phenotypic representation: a novel de novo loss of function mutation in a case with childhood onset schizophrenia. Alkelai A , Shohat S , Greenbaum L , Schechter T , Draiman B , Chitrit-Raveh E , Rienstein S , Dagaonkar N , Hughes D , Aggarwal VS , Heinzen EL , Shifman S , Goldstein DB , Kohn Y . | No Clinical |
| 25. | Prim Care Companion CNS Disord. 2020 May 28;22 :19l02515. doi: 10.4088/PCC.19l02515. Co-Occurrence of Autistic Spectrum Disorder and Childhood-Onset Schizophrenia. Hatila S , Solanki G | No Clinical |
| **26.** | **Early Interv Psychiatry. 2021 Apr;15 :412-419. doi: 10.1111/eip.12973. Epub 2020 May 20. Functional outcomes and patient satisfaction following inpatient treatment for childhood-onset schizophrenia spectrum disorders vs non-psychotic disorders in children in the United Kingdom. Galitzer H , Anagnostopoulou N , Alba A , Gaete J , Dima D , Kyriakopoulos M .** | **Included** |
| 27. | Compr Psychiatry. 2020 Jul;100:152176. doi: 10.1016/j.comppsych.2020.152176. Epub 2020 Apr 15.Sex differences in circulating neuregulin1-β1 and β-secretase 1 expression in childhood-onset schizophrenia. Zhang Z , Li Y , He F , Cui Y , Zheng Y , Li R . | No Clinical |
| 28. | J Sleep Res. 2021 Apr;30 :e13039. doi: 10.1111/jsr.13039. Epub 2020 Apr 30. Sleep neurophysiology in childhood onset schizophrenia. Markovic A , Buckley A , Driver DI , Dillard-Broadnax D , Gochman PA , Hoedlmoser K , Rapoport JL , Tarokh L . | No Clinical |
| 29. | Front Genet. 2019 Dec 18;10:1137. doi: 10.3389/fgene.2019.01137. eCollection 2019. Childhood-Onset Schizophrenia: A Systematic Overview of Its Genetic Heterogeneity From Classical Studies to the Genomic Era. Fernandez A , Drozd MM , Thümmler S , Dor E , Capovilla M , Askenazy F Bardoni B . | Review |
| 30. | BMC Med Genet. 2020 Jan 8;21 :10. doi: 10.1186/s12881-019-0946-0. Chromatin remodeling dysfunction extends the etiological spectrum of schizophrenia: a case report. Poisson A , Chatron N , Labalme A , Fourneret P , Ville D , Mathieu ML , Sanlaville D , Demily C , Lesca G . | Case Report |
| **31.** | **Brain Behav. 2020 Feb;10 :e01495. doi: 10.1002/brb3.1495. Epub 2020 Jan 7. Early and very early-onset schizophrenia compared with adult-onset schizophrenia: French FACE-SZ database. Coulon N , Godin O , Bulzacka E , Dubertret C , Mallet J , Fond G , Brunel L , Andrianarisoa M , Anderson G , Chereau I , Denizot H , Rey R , Dorey JM , Lançon C , Faget C , Roux P , Passerieux C , Dubreucq J , Leignier S , Capdevielle D , André M , Aouizerate B , Misdrahi D , Berna F , Vidailhet P , Leboyer M , Schürhoff F .** | **Included** |
| 32. | J Can Acad Child Adolesc Psychiatry. 2019 Nov;28 :147-150. Epub 2019 Nov 1. Case Report of Childhood-Onset Psychosis in a Patient with a Known WNT10A Mutation.Kobza AO , Alenezi S . | Case Report |
| 33. | Child Adolesc Psychiatr Clin N Am. 2020 Jan;29 :71-90. doi: 10.1016/j.chc.2019.08.017. Childhood-Onset Schizophrenia and Early-onset Schizophrenia Spectrum Disorders: An Update. Driver DI , Thomas S , Gogtay N , Rapoport JL . | Review |
| 34. | Child Adolesc Psychiatr Clin N Am. 2020 Jan;29 :157-170. doi: 10.1016/j.chc.2019.08.007. Epub 2019 Oct 17. Genetics of Childhood-onset Schizophrenia 2019 Update. Forsyth JK , Asarnow RF . | Review |
| 35. | Int J Dev Neurosci. 2019 Dec;79:49-53. doi: 10.1016/j.ijdevneu.2019.10.006. Epub 2019 Oct 31. Effects of ketamine on prepubertal Wistar rats: Implications on behavioral parameters for Childhood-Onset Schizophrenia. Damazio Pacheco F , Canever L , Antunes Mastella G , Gomes Wessler P , Kunz Godoi A , Hubbe I , da Costa Afonso A , Celso D , Quevedo J , Ioppi Zugno A . | No VEOS |
| 36. | Psychol Med. 2020 Jul;50 :1672-1679. doi: 10.1017/S0033291719001715. Epub 2019 Jul 31. Evidence of shared familial factors influencing neurocognitive endophenotypes in adult- and childhood-onset schizophrenia. Bigdeli TB , Nuechterlein KH , Sugar CA , Subotnik KL , Kubarych T , Neale MC , Kendler KS , Asarnow RF . | No VEOS |
| 37. | J Mol Neurosci. 2019 Nov;69 :485-493. doi: 10.1007/s12031-019-01382-0. Epub 2019 Jul 13. Age Matters: an Atypical Association Between Polymorphism of MTHFR and Clinical Phenotypes in Children with Schizophrenia. Wan L , Li Y , Zhou Y , Li R , Zheng Y . | No Clinical |
| 38. | Cell Rep. 2019 Jun 25;27 :3832-3843.e6. doi: 10.1016/j.celrep.2019.05.088. Dysregulated Glial Differentiation in Schizophrenia May Be Relieved by Suppression of SMAD4- and REST-Dependent Signaling. Liu Z , Osipovitch M , Benraiss A , Huynh NPT , Foti R , Bates J , Chandler-Militello D , Findling RL , Tesar PJ , Nedergaard M , Windrem MS , Goldman SA . | No Clinical |
| 39. | Am J Ther. 2019 May/Jun;26 :e406-e416. doi: 10.1097/MJT.0000000000000894. Off-Label Use of Clozapine in Children and Adolescents-A Literature Review. Rachamallu V , Elberson BW , Vutam E , Aligeti M . | Review |
| 40. | J Child Adolesc Psychopharmacol. 2019 Apr;29 :241-244. doi: 10.1089/cap.2019.29164.bjc. Complexity in Evaluation and Pharmacological Treatment of Early Onset Psychosis with Mood Symptoms: Childhood Onset Schizophrenia or Affective Disorder? Jerath AU , Mavrides NA , Coffey BJ . | No VEOS |
| 41. | Encephale. 2018 Dec;44(6S):S8-S11. doi: 10.1016/S0013-7006(19)30071-5. [Very early onset schizophrenia]. Da Fonseca D , Fourneret P . | Review |
| 42. | Arch Med Sci. 2019 Jan;15 :126-133. doi: 10.5114/aoms.2018.73422. Epub 2018 Feb 12. Amplitude of low-frequency fluctuations in childhood-onset schizophrenia with or without obsessive-compulsive symptoms: a resting-state functional magnetic resonance imaging study. Liang Y , Shao R , Zhang Z , Li X , Zhou L , Guo S . | No Clinical |
| 43. | Int J Mol Sci. 2018 Nov 30;19 :3829. doi: 10.3390/ijms19123829. Childhood-Onset Schizophrenia: Insights from Induced Pluripotent Stem Cells. Hoffmann A , Ziller M , Spengler D . | Review |
| 44. | Am J Med Genet B Neuropsychiatr Genet. 2019 Sep;180 :335-340. doi: 10.1002/ajmg.b.32683. Epub 2018 Oct 30.Exome sequencing of sporadic childhood-onset schizophrenia suggests the contribution of X-linked genes in males.Ambalavanan A , Chaumette B , Zhou S , Xie P , He Q , Spiegelman D , Dionne-Laporte A , Bourassa CV , Therrien M , Rochefort D , Xiong L , Dion PA , Joober R , Rapoport JL , Girard SL , Rouleau GA . | No Clinical |
| 45. | Schizophr Res. 2018 Dec;202:431-432. doi: 10.1016/j.schres.2018.07.023. Epub 2018 Jul 18. 7 T MRI reveals hippocampal structural abnormalities associated with memory intrusions in childhood-onset schizophrenia. Zhou D , Liu S , Zhou X , Berman R , Broadnax D , Gochman P , Rapoport J , Thomas A. | No Clinical |
| 46. | Mol Psychiatry. 2020 Apr;25 :821-830. doi: 10.1038/s41380-018-0103-8. Epub 2018 Jun 12. Missense variants in ATP1A3 and FXYD gene family are associated with childhood-onset schizophrenia. Chaumette B , Ferrafiat V , Ambalavanan A , Goldenberg A , Dionne-Laporte A , Spiegelman D , Dion PA , Gerardin P , Laurent C , Cohen D , Rapoport J , Rouleau GA . | No Clinical |
| 47. | Psychiatry Res. 2018 Aug;266:317-322. doi: 10.1016/j.psychres.2018.03.030. Epub 2018 Mar 16. The effects of antipsychotics on the density of cannabinoid receptors in selected brain regions of male and female adolescent juvenile rats. Lian J , Deng C . | Unrelated |
| 48. | Schizophr Res. 2018 Sep;199:135-141. doi: 10.1016/j.schres.2018.03.027. Epub 2018 Mar 19. Suspiciousness in young minds: Convergent evidence from non-clinical, clinical and community twin samples. Zhou HY , Wong KK , Shi LJ , Cui XL , Qian Y , Jiang WQ , Du YS , Lui SSY , Luo XR , Yi ZH , Cheung EFC , Docherty AR , Chan RCK . | No VEOS |
| 49. | Asian J Psychiatr. 2018 Mar;33:61-62. doi: 10.1016/j.ajp.2017.10.020. Epub 2017 Oct 23. Very early onset schizophrenia diagnostic challenge and cognitive remediation-A case report. Jakhar J , Linganna S , Seshadri SP . | Case Report |
| 50. | J Am Acad Child Adolesc Psychiatry. 2018 Mar;57 :166-174. doi: 10.1016/j.jaac.2017.12.009. Epub 2017 Dec 28.Reduced Functional Brain Activation and Connectivity During a Working Memory Task in Childhood-Onset Schizophrenia. Loeb FF , Zhou X , Craddock KES , Shora L , Broadnax DD , Gochman P , Clasen LS , Lalonde FM , Berman RA , Berman KF , Rapoport JL , Liu S . | No Clinical |
| 51. | J Am Acad Child Adolesc Psychiatry. 2018 Mar;57 :143-145. doi: 10.1016/j.jaac.2018.01.005. Adults With Childhood-Onset Schizophrenia and Their Siblings: Do Age of Onset and Familiality Affect Performance on and the Neural Signature of Working Memory Tasks? Frazier JA . | No VEOS |
| 52. | Schizophr Res. 2018 Jul;197:219-225. doi: 10.1016/j.schres.2018.01.003. Epub 2018 Jan 6. Attenuated resting-state functional connectivity in patients with childhood- and adult-onset schizophrenia. Watsky RE , Gotts SJ , Berman RA , McAdams HM , Zhou X , Greenstein D , Lalonde FM , Gochman P , Clasen LS , Shora L , Ordóñez AE , Gogtay N , Martin A , Barch DM , Rapoport JL , Liu S . | No Clinical |
| **53.** | **Schizophr Res. 2018 Jul;197:71-77. doi: 10.1016/j.schres.2017.10.045. Epub 2017 Nov 13. Symptom dimensions and subgroups in childhood-onset schizophrenia. Craddock KES , Zhou X , Liu S , Gochman P , Dickinson D , Rapoport JL .** | **Included** |
| 54. | Indian J Psychol Med. 2017 Jul-Aug;39 :519-522. doi: 10.4103/0253-7176.211739. Very Early-onset Schizophrenia with Secondary Onset Tic Disorder. Telgote SA , Pendharkar SS , Kelkar AD , Bhojane S. | Case Report |
| 55. | Cell Stem Cell. 2017 Aug 3;21 :195-208.e6. doi: 10.1016/j.stem.2017.06.012. Epub 2017 Jul 20. Human iPSC Glial Mouse Chimeras Reveal Glial Contributions to Schizophrenia. Windrem MS , Osipovitch M , Liu Z , Bates J , Chandler-Militello D , Zou L , Munir J , Schanz S , McCoy K , Miller RH , Wang S , Nedergaard M , Findling RL , Tesar PJ , Goldman SA . | Unrelated |
| 56. | Synapse. 2017 Oct;71 :e21988. doi: 10.1002/syn.21988. Epub 2017 Jun 27. Developmental effects of antipsychotic drugs on serotonin receptor subtypes. Choi YK , Gardner MP , Tarazi FI . | Unrelated |
| 57. | Schizophr Res. 2018 Feb;192:39-49. doi: 10.1016/j.schres.2017.05.011. Epub 2017 May 16. An overview of medical risk factors for childhood psychosis: Implications for research and treatment. Giannitelli M , Consoli A , Raffin M , Jardri R , Levinson DF , Cohen D , Laurent-Levinson C . | Review |
| 58. | Hum Psychopharmacol. 2017 Mar;32 . doi: 10.1002/hup.2589. Diagnostic clusters associated with an early onset schizophrenia diagnosis among children and adolescents. Jerrell JM , McIntyre RS , Deroche CB . | Lumping |
| 59. | J Child Adolesc Psychopharmacol. 2016 Dec;26 :944-947. doi: 10.1089/cap.2016.29120.bjc. Challenges in the Psychopharmacological Management of Very Early-Onset Schizophrenia and Anxiety. Mehdi A , Schweinsburg BC , Zehgeer A , Connor DF , Luber MJ , Coffey BJ . | No Clinical |
| 60. | Psychiatry Res Neuroimaging. 2016 Dec 30;258:23-29. doi: 10.1016/j.pscychresns.2016.10.010. Epub 2016 Nov 1. DTI microstructural abnormalities in adolescent siblings of patients with childhood-onset schizophrenia. Waltzman D , Knowlton BJ , Cohen JR , Bookheimer SY , Bilder RM ,Asarnow RF . | No Clinical |
| 61. | Cold Spring Harb Mol Case Stud. 2016 Sep;2 :a001008. doi: 10.1101/mcs.a001008. A novel de novo mutation in ATP1A3 and childhood-onset schizophrenia. Smedemark-Margulies N , Brownstein CA , Vargas S , Tembulkar SK , Towne MC , Shi J , Gonzalez-Cuevas E , Liu KX , Bilguvar K , Kleiman RJ , Han MJ , Torres A , Berry GT , Yu TW , Beggs AH , Agrawal PB , Gonzalez-Heydrich J . | Case Report |
| **62.** | **J Am Acad Child Adolesc Psychiatry. 2016 Sep;55 :792-9. doi: 10.1016/j.jaac.2016.05.022. Epub 2016 Jul 1. Lack of Gender-Related Differences in Childhood-Onset Schizophrenia. Ordóñez AE , Loeb FF , Zhou X , Shora L , Berman RA , Broadnax DD , Gochman P , Liu S , Rapoport JL .** | **Included** |
| 63. | Focus (Am Psychiatr Publ). 2016 Jul;14 :328-332. doi: 10.1176/appi.focus.20160007. Epub 2016 Jul 8. A Review of Childhood-Onset Schizophrenia. Kendhari J , Shankar R , Young-Walker L . | Review |
| 64. | J Neural Transm (Vienna). 2016 Oct;123 :1219-34. doi: 10.1007/s00702-016-1572-z. Epub 2016 May 12. Transcranial direct current stimulation in children and adolescents: a comprehensive review. Palm U , Segmiller FM , Epple AN , Freisleder FJ , Koutsouleris N , Schulte-Körne G , Padberg F . | Review |
| 65. | Acad Pediatr. 2016 Aug;16 :508-18. doi: 10.1016/j.acap.2016.03.011. Epub 2016 Apr 5. Challenges and Promises of Pediatric Psychopharmacology. Giles LL , Martini DR . | Unrelated |
| 66. | Zh Nevrol Psikhiatr Im S S Korsakova. 2016;116 :85-89. doi: 10.17116/jnevro20161162185-89. [The possibility of using immunological parameters for the evaluation of the clinical state of patients with childhood-onset schizophrenia]. Androsova LV , Simashkova NV , Shushpanova OV , Otman IN , Kliushnik TP . | No Clinical |
| 67. | J Child Adolesc Psychopharmacol. 2016 Sep;26 :590-7. doi: 10.1089/cap.2015.0172. Epub 2016 Mar 30. Transcranial Direct Current Stimulation in Child and Adolescent Psychiatry. Muszkat D , Polanczyk GV , Dias TG , Brunoni AR . | Review |
| 68. | Am J Med Genet B Neuropsychiatr Genet. 2016 Sep;171 :777-83. doi: 10.1002/ajmg.b.32439. Epub 2016 Mar 10. 15q13.3 duplication in two patients with childhood-onset schizophrenia. Zhou D , Gochman P , Broadnax DD , Rapoport JL , Ahn K . | Case Report |
| 69. | J Am Acad Child Adolesc Psychiatry. 2016 Feb;55 :130-136. doi: 10.1016/j.jaac.2015.11.008. Epub 2015 Nov 26. Severity of Cortical Thinning Correlates With Schizophrenia Spectrum Symptoms. Watsky RE(#) , Pollard KL(#) , Greenstein D , Shora L , Dillard-Broadnax D , Gochman P , Clasen LS , Berman RA , Rapoport JL , Gogtay N , Ordóñez AE . | No VEOS |
| 70. | Neuroimage Clin. 2015 Nov 18;10:96-106. doi: 10.1016/j.nicl.2015.11.011. eCollection 2016. Network community structure alterations in adult schizophrenia: identification and localization of alterations. Lerman-Sinkoff DB , Barch DM . | No VEOS |
| 71. | J Child Adolesc Psychopharmacol. 2016 Jun;26 :428-35. doi: 10.1089/cap.2015.0103. Epub 2016 Jan 19. Strong Treatment Response and High Maintenance Rates of Clozapine in Childhood-Onset Schizophrenia. Kasoff LI , Ahn K , Gochman P , Broadnax DD , Rapoport JL . | No Clinical |
| 72. | J Child Adolesc Psychopharmacol. 2016 Nov;26 :815-821. doi: 10.1089/cap.2015.0020. Epub 2016 Jan 15. A Retrospective Investigation of Clozapine Treatment in Autistic and Nonautistic Children and Adolescents in an Inpatient Clinic in Turkey. Yalcin O , Kaymak G , Erdogan A , Tanidir C , Karacetin G , Kilicoglu AG , Mutlu C , Adaletli H , Gunes H , Bahali K , Ayik B , Uneri OS . | No VEOS |
| 73. | Brain. 2016 Jan;139(Pt 1):10-2. doi: 10.1093/brain/awv330. Altered intra- and inter-network dynamics reflect symptom dimensions in childhood-onset schizophrenia. Lancaster TM , Hall J . | No Clinical |
| 74. | Eur J Hum Genet. 2016 Jun;24 :944-8. doi: 10.1038/ejhg.2015.218. Epub 2015 Oct 28. De novo variants in sporadic cases of childhood onset schizophrenia. Ambalavanan A , Girard SL , Ahn K , Zhou S , Dionne-Laporte A , Spiegelman D , Bourassa CV , Gauthier J , Hamdan FF , Xiong L , Dion PA , Joober R , Rapoport J , Rouleau GA . | No Clinical |
| 75. | Brain. 2016 Jan;139(Pt 1):276-91. doi: 10.1093/brain/awv306. Epub 2015 Oct 22. Disrupted sensorimotor and social-cognitive networks underlie symptoms in childhood-onset schizophrenia. Berman RA , Gotts SJ , McAdams HM , Greenstein D , Lalonde F , Clasen L , Watsky RE , Shora L , Ordonez AE , Raznahan A , Martin A , Gogtay N , Rapoport J . | No Clinical |
| 76. | Microbes Infect. 2016 Feb;18 :153-8. doi: 10.1016/j.micinf.2015.09.023. Epub 2015 Oct 9. Infection and characterization of Toxoplasma gondii in human induced neurons from patients with brain disorders and healthy controls. Passeri E , Jones-Brando L , Bordón C , Sengupta S , Wilson AM , Primerano A , Rapoport JL , Ishizuka K , Kano S , Yolken RH , Sawa A . | Unrelated |
| 77. | Schizophr Res. 2015 Oct;168(1-2):252-9. doi: 10.1016/j.schres.2015.07.039. Epub 2015 Aug 18. Facial, vocal and cross-modal emotion processing in early-onset schizophrenia spectrum disorders. Giannitelli M , Xavier J , François A , Bodeau N , Laurent C , Cohen D , Chaby L . | No VEOS |
| 78. | Neurosci Res. 2015 Dec;101:57-61. doi: 10.1016/j.neures.2015.07.011. Epub 2015 Aug 7. Enhanced conversion of induced neuronal cells (iN cells) from human fibroblasts: Utility in uncovering cellular deficits in mental illness-associated chromosomal abnormalities. Passeri E , Wilson AM , Primerano A , Kondo MA , Sengupta S , Srivastava R , Koga M , Obie C , Zandi PP , Goes FS , Valle D , Rapoport JL , Sawa A , Kano S , Ishizuka K . | Unrelated |
| 79. | CNS Spectr. 2015 Aug;20 :442-50. doi: 10.1017/S1092852915000437. Functional and clinical insights from neuroimaging studies in childhood-onset schizophrenia. Ordóñez AE , Sastry NV , Gogtay N . | Review |
| 80. | JAMA Psychiatry. 2015 Sep;72 :900-8. doi: 10.1001/jamapsychiatry.2015.0226. Delayed Development of Brain Connectivity in Adolescents With Schizophrenia and Their Unaffected Siblings. Zalesky A , Pantelis C , Cropley V , Fornito A , Cocchi L , McAdams H , Clasen L , Greenstein D , Rapoport JL , Gogtay N . | No Clinical |
| 81. | Neuroscience. 2015 Sep 10;303:82-102. doi: 10.1016/j.neuroscience.2015.06.028. Epub 2015 Jun 23. The neuropathology of schizophrenia: A selective review of past studies and emerging themes in brain structure and cytoarchitecture. Bakhshi K , Chance SA . | Review |
| 82. | Genetika. 2015 Feb;51 :227-35. [Replicative study of susceptibility to childhood-onset schizophrenia in Kazakhs]. Stepanov VA, Bocharova AV, Saduakassova KZ, Marusin AV, Koneva LA, Vagaitseva KV, Svyatova GS. | No Clinical |
| 83. | Psychol Med. 2015;45 :2667-74. doi: 10.1017/S0033291715000677. Epub 2015 May 4. Hippocampal volume change relates to clinical outcome in childhood-onset schizophrenia. Anvari AA , Friedman LA , Greenstein D , Gochman P , Gogtay N , Rapoport JL . | No Clinical |
| 84. | Clin Case Rep. 2015 Apr;3 :201-7. doi: 10.1002/ccr3.192. Epub 2015 Feb 2. Childhood-onset schizophrenia case with 2.2 Mb deletion at chromosome 3p12.2-p12.1 and two large chromosomal abnormalities at 16q22.3-q24.3 and Xq23-q28. Rudd D , Axelsen M , Epping EA , Andreasen N , Wassink T . | Case Report |
| 85. | Neuropsychobiology. 2015;71 :120-124. doi: 10.1159/000370077. Epub 2015 Apr 9. Effect of Clozapine and Other Antipsychotics on the Level of Platelet-Associated Autoantibodies in Children with Schizophrenia: A Longitudinal Follow-Up Study. Ebert T , Schechtman M, Midbari Y, Weizman A, Shinitzky M, Spivak B. | No Clinical |
| 86. | Schizophr Res. 2016 Jun;173 :124-131. doi: 10.1016/j.schres.2015.03.003. Epub 2015 Mar 26. Neuroimaging findings from childhood onset schizophrenia patients and their non-psychotic siblings. Ordóñez AE , Luscher ZI , Gogtay N . | No Clinical |
| 87. | Br J Psychiatry. 2015 Jun;206 :517-8. doi: 10.1192/bjp.bp.114.158493. Epub 2015 Mar 19. Incidence and 12-month outcome of childhood non-affective psychoses: British national surveillance study. Tiffin PA , Kitchen CE . | No Data |
| 88. | Schizophr Res. 2015 Feb;161(2-3):345-50. doi: 10.1016/j.schres.2014.12.006. Epub 2014 Dec 22. Cognitive correlates of gray matter abnormalities in adolescent siblings of patients with childhood-onset schizophrenia. Wagshal D , Knowlton BJ , Cohen JR , Bookheimer SY , Bilder RM , Fernandez VG , Asarnow RF . | No VEOS |
| 89. | Australas Psychiatry. 2015 Feb;23 :63-5. doi: 10.1177/1039856214563844. Epub 2014 Dec 17. Psychosocial short stature with psychosis: a case report. Wattchow N , Lee HE , Brock P . | Case Report |
| 90. | Mol Psychiatry. 2016 Jan;21 :94-6. doi: 10.1038/mp.2014.158. Epub 2014 Dec 16. Common polygenic variation and risk for childhood-onset schizophrenia. Ahn K , An SS , Shugart YY , Rapoport JL . | No Clinical |
| 91. | Hum Brain Mapp. 2015 Apr;36 :1458-69. doi: 10.1002/hbm.22715. Epub 2014 Dec 11. Striatal shape abnormalities as novel neurodevelopmental endophenotypes in schizophrenia: a longitudinal study. Chakravarty MM , Rapoport JL, Giedd JN, Raznahan A, Shaw P, Collins DL, Lerch JP, Gogtay N. | No Clinical |
| 92. | J Child Adolesc Ment Health. 2014;26 :109-24. doi: 10.2989/17280583.2014.924416. New developments in diagnosis and treatment update: Schizophrenia/first episode psychosis in children and adolescents. Lachman A . | Review |
| 93. | Schizophr Bull. 2015 Jan;41 :66-73. doi: 10.1093/schbul/sbu123. Epub 2014 Sep 12. Comparing fractional anisotropy in patients with childhood-onset schizophrenia, their healthy siblings, and normal volunteers through DTI. Moran ME , Luscher ZI , McAdams H , Hsu JT , Greenstein D , Clasen L , Ludovici K , Lloyd J , Rapoport J , Mori S , Gogtay N . | No Clinical |
| 94. | Neuropsychopharmacology. 2015 Jan;40 :43-9. doi: 10.1038/npp.2014.236. Epub 2014 Sep 8. Child psychiatry branch of the National Institute of Mental Health longitudinal structural magnetic resonance imaging study of human brain development. Giedd JN , Raznahan A , Alexander-Bloch A , Schmitt E , Gogtay N , Rapoport JL . | No Clinical |
| **95.** | **J Child Adolesc Psychopharmacol. 2014 Sep;24 :366-73. doi: 10.1089/cap.2013.0139. Epub 2014 Jul 14. Looking for childhood-onset schizophrenia: diagnostic algorithms for classifying children and adolescents with psychosis. Greenstein D , Kataria R, Gochman P, Dasgupta A, Malley JD, Rapoport J, Gogtay N.** | **Included** |
| 96. | Rev Prat. 2014 Apr;64 :499-504. [Schizophrenic disorders in adolescence]. Bonnot O, Dumas N. | Review |
| 97. | Biol Psychiatry. 2014 Sep 15;76 :438-46. doi: 10.1016/j.biopsych.2014.02.010. Epub 2014 Feb 22. Abnormal cortical growth in schizophrenia targets normative modules of synchronized development. Alexander-Bloch AF , Reiss PT , Rapoport J , McAdams H , Giedd JN , Bullmore ET , Gogtay N . | No Clinical |
| 98. | Front Psychiatry. 2013 Dec 20;4:175. doi: 10.3389/fpsyt.2013.00175. A comparison of neuroimaging findings in childhood onset schizophrenia and autism spectrum disorder: a review of the literature. Baribeau DA , Anagnostou E . | Review |
| 99. | Health Psychol Behav Med. 2014 Jan 1;2 :735-747. doi: 10.1080/21642850.2014.927738. Epub 2014 Jul 15. Childhood-onset schizophrenia: what do we really know? Bartlett J . | Review |
| 100. | Zh Nevrol Psikhiatr Im S S Korsakova. 2014;114 :42-47. doi: 10.17116/jnevro201411412142-47. [Variants of cognitive development in children and adolescents with different forms of schizophrenia]. Zvereva NV , Khromov AI . | Lumping |
| 101. | Schizophr Bull. 2014 Sep;40 :1030-9. doi: 10.1093/schbul/sbt147. Epub 2013 Oct 25. Evidence for corticostriatal dysfunction during cognitive skill learning in adolescent siblings of patients with childhood-onset schizophrenia. Wagshal D , Knowlton BJ , Suthana NA , Cohen JR , Poldrack RA , Bookheimer SY , Bilder RM , Asarnow RF . | No Clinical |
| 102. | J Child Adolesc Psychopharmacol. 2013 Oct;23 :585-6. doi: 10.1089/cap.2013.0033. Aspirin as an adjunctive treatment for childhood onset schizophrenia. Webb JR , Stubbe DE, Poncin YB. | Unrelated |
| 103. | J Child Adolesc Psychopharmacol. 2013 Oct;23 :516-21. doi: 10.1089/cap.2013.0050. Epub 2013 Oct 10. Hematological and cardiometabolic safety of clozapine in the treatment of very early onset schizophrenia: a retrospective chart review. Midbari Y , Ebert T, Kosov I, Kotler M, Weizman A, Ram A. | Review |
| 104. | Child Adolesc Psychiatr Clin N Am. 2013 Oct;22 :689-714. doi: 10.1016/j.chc.2013.06.003. Epub 2013 Jul 23. Gray matter alterations in schizophrenia high-risk youth and early-onset schizophrenia: a review of structural MRI findings. Brent BK , Thermenos HW, Keshavan MS, Seidman LJ. | Review |
| 105. | Child Adolesc Psychiatr Clin N Am. 2013 Oct;22 :675-87. doi: 10.1016/j.chc.2013.06.004. Epub 2013 Jul 23. Genetics of childhood-onset schizophrenia. Asarnow RF , Forsyth JK. | Review |
| 106. | Child Adolesc Psychiatr Clin N Am. 2013 Oct;22 :539-55. doi: 10.1016/j.chc.2013.04.001. Epub 2013 Jun 18. Childhood onset schizophrenia and early onset schizophrenia spectrum disorders. Driver DI , Gogtay N, Rapoport JL. | Review |
| 107. | Neuropsychobiology. 2013;68 :124-7. doi: 10.1159/000353267. Epub 2013 Jul 19. High circulatory titer of platelet-associated autoantibodies in childhood onset schizophrenia and its diagnostic implications. Ebert T , Schechtman M, Ram A, Kosov I, Weizman A, Shinitzky M, Spivak B. | No Clinical |
| 108. | Hum Mol Genet. 2013 Dec 1;22(23):4673-87. doi: 10.1093/hmg/ddt315. Epub 2013 Jul 2. The UPF3B gene, implicated in intellectual disability, autism, ADHD and childhood onset schizophrenia regulates neural progenitor cell behaviour and neuronal outgrowth. Jolly LA , Homan CC, Jacob R, Barry S, Gecz J. | Unrelated |
| 109. | J Child Adolesc Psychopharmacol. 2013 Jun;23 :363-6. doi: 10.1089/cap.2013.2353. Unremitting impulsive aggression in a child with childhood onset schizophrenia and pervasive development disorder-not otherwise specified: the role of stimulants, atypical antipsychotics and mood stabilizers. Taskiran S , Coffey BJ. | Case Report |
| 110. | Int J Dev Neurosci. 2014 Feb;32:58-63. doi: 10.1016/j.ijdevneu.2013.05.010. Epub 2013 Jun 11. At the boundary of the self: the insular cortex in patients with childhood-onset schizophrenia, their healthy siblings, and normal volunteers. Moran ME , Weisinger B , Ludovici K , McAdams H , Greenstein D , Gochman P , Miller R , Clasen L , Rapoport J , Gogtay N . | No Clinical |
| 111. | J Clin Psychopharmacol. 2013 Aug;33 :572-4. doi: 10.1097/JCP.0b013e3182946719. Clozapine augmented with amisulpride in 3 cases of treatment-resistant early- and very early-onset schizophrenia. Tufan AE, Yalug I. | No Clinical |
| 112. | Arch Pediatr. 2013 Jul;20 :789-99. doi: 10.1016/j.arcped.2013.04.021. Epub 2013 May 31. Childhood onset schizophrenia: current data and therapeutic approach. Fourneret P , Georgieff N, Franck N. | Review |
| 113. | Brain. 2013 Nov;136(Pt 11):3215-26. doi: 10.1093/brain/awt116. Epub 2013 May 22. A family affair: brain abnormalities in siblings of patients with schizophrenia. Moran ME , Hulshoff Pol H, Gogtay N. | No Clinical |
| 114. | Mol Psychiatry. 2014 May;19 :568-72. doi: 10.1038/mp.2013.59. Epub 2013 May 21. High rate of disease-related copy number variations in childhood onset schizophrenia. Ahn K , Gotay N , Andersen TM , Anvari AA , Gochman P , Lee Y , Sanders S , Guha S , Darvasi A , Glessner JT , Hakonarson H , Lencz T , State MW , Shugart YY , Rapoport JL . | No Clinical |
| 115. | Iran J Psychiatry. 2013 Mar;8 :44-50. Reality Testing in Children with Childhood-Onset Schizophrenia and Normal Children: A Comparison using the Ego Impairment Index on the Rorschach. Mohammadi MR , Hosseininasab A, Borjali A, Mazandarani AA. | No Data |
| 116. | Nihon Rinsho. 2013 Apr;71 :701-5.Childhood-onset schizophrenia Kimoto K , Matsumoto H. Childhood-onset schizophrenia has been clinically studied and is believed to be on a continuum with adult-onset schizophrenia. However, childhood-onset | Review |
| 117. | J Am Acad Child Adolesc Psychiatry. 2013 May;52 :527-536.e2. doi: 10.1016/j.jaac.2013.02.003. Epub 2013 Apr 3. Hippocampal shape abnormalities of patients with childhood-onset schizophrenia and their unaffected siblings. Johnson SL , Wang L, Alpert KI, Greenstein D, Clasen L, Lalonde F, Miller R, Rapoport J, Gogtay N. | No Clinical |
| 118. | Expert Rev Neurother. 2013 Apr;13 :447-58. doi: 10.1586/ern.13.29. Treatments in context: transcranial direct current brain stimulation as a potential treatment in pediatric psychosis. David CN , Rapoport JL, Gogtay N. | No Clinical |
| 119. | J Child Adolesc Psychopharmacol. 2013 Mar;23 :110-6. doi: 10.1089/cap.2011.0136. Risk factors for neutropenia in clozapine-treated children and adolescents with childhood-onset schizophrenia. Maher KN , Tan M, Tossell JW, Weisinger B, Gochman P, Miller R, Greenstein D, Overman GP, Rapoport JL, Gogtay N. | Review |
| 120. | Am J Med Genet A. 2013 Apr;161A :845-9. doi: 10.1002/ajmg.a.35754. Epub 2013 Feb 26. Co-occurrence of autism, childhood psychosis, and intellectual disability associated with a de novo 3q29 microdeletion. Sagar A , Bishop JR, Tessman DC, Guter S, Martin CL, Cook EH. | Case Report |
| 121. | Ment Illn. 2013 Feb 11;5 :e2. doi: 10.4081/mi.2013.e2. eCollection 2013 Feb 11. Violence in childhood-onset schizophrenia. Ross RG , Maximon J , Kusumi J , Lurie S . | Review |
| 122. | Psychiatry Res. 2013 Jan 30;211 :11-6. doi: 10.1016/j.pscychresns.2012.09.013. Epub 2012 Nov 13. Absence of anatomic corpus callosal abnormalities in childhood-onset schizophrenia patients and healthy siblings. Johnson SL , Greenstein D, Clasen L, Miller R, Lalonde F, Rapoport J, Gogtay N. | No Clinical |
| 123. | Aust N Z J Psychiatry. 2013 Jan;47 :43-50. doi: 10.1177/0004867412463615. Epub 2012 Oct 9. The presentation of early-onset psychotic disorders. Starling J , Williams LM, Hainsworth C, Harris AW. | No VEOS |
| 124. | Zh Nevrol Psikhiatr Im S S Korsakova. 2012;112 :20-7. The follow-up study of childhood-onset schizophrenia: clinical and social aspects Mazaeva NA, Shmakova OP, Andreeva OO. | No VEOS |
| 125. | Clin Ter. 2012 Jul;163 :e189-92. ADHD or childhood-onset schizophrenia? A case report Lia C , Cavaggioni G. | Case Report |
| 126. | Arch Gen Psychiatry. 2012 Sep;69 :875-84. doi: 10.1001/archgenpsychiatry.2011.2084. Delayed white matter growth trajectory in young nonpsychotic siblings of patients with childhood-onset schizophrenia. Gogtay N , Hua X, Stidd R, Boyle CP, Lee S, Weisinger B, Chavez A, Giedd JN, Clasen L, Toga AW, Rapoport JL, Thompson PM. | No Clinical |
| 127. | Schizophr Res. 2012 Sep;140(1-3):149-54. doi: 10.1016/j.schres.2012.07.006. Epub 2012 Jul 24. Psychotic symptoms and gray matter deficits in clinical pediatric populations. Gogtay N , Weisinger B, Bakalar JL, Stidd R, Fernandez de la Vega O, Miller R, Clasen L, Greenstein D, Rapoport JL. | No Clinical |
| 128. | Ment Illn. 2012 Oct 15;4 :e22. doi: 10.4081/mi.2012.e22. eCollection 2012 Jul 26. Prevalence of psychiatric illness in primary caretakers of childhood-onset schizophrenia subjects. Kusumi J , Ross RG . | No VEOS |
| 129. | Neuropsychobiology. 2012;66 :63-9. doi: 10.1159/000338548. Epub 2012 Jul 13. Early-onset schizophrenia. Remschmidt H , Theisen F. | Review |
| 130. | Psychiatry Res. 2012 Dec 30;200(2-3):167-72. doi: 10.1016/j.psychres.2012.06.009. Epub 2012 Jul 2.Deficits in probabilistic classification learning and liability for schizophrenia. Wagshal D , Knowlton BJ, Cohen JR, Poldrack RA, Bookheimer SY, Bilder RM, Fernandez VG, Asarnow RF. | No Clinical |
| 131. | Front Psychiatry. 2012 Jun 1;3:53. doi: 10.3389/fpsyt.2012.00053. eCollection 2012. Using multivariate machine learning methods and structural MRI to classify childhood onset schizophrenia and healthy controls. Greenstein D , Malley JD, Weisinger B, Clasen L, Gogtay N. | No Clinical |
| 132. | Psychiatriki. 2012 Jun;23 Suppl 1:82-93. Schizophrenia in children and adolescents: relevance and differentiation from adult schizophrenia Androutsos Ch . | Review |
| 133. | Chin Med J (Engl). 2012 Apr;125 :1349-51. A case report on the relationship between treatment-resistant childhood-onset schizophrenia and an abnormally enlarged cavum septum pellucidum combined with cavum vergae. Liao ZL , Hu SH, Xu Y. | Case Report |
| 134. | Psychiatr Genet. 2012 Aug;22 :206-9. doi: 10.1097/YPG.0b013e328353ae3d. Microduplications disrupting the MYT1L gene (2p25.3) are associated with schizophrenia. Lee Y , Mattai A, Long R, Rapoport JL, Gogtay N, Addington AM. | Review |
| 135. | J Neuropsychiatry Clin Neurosci. 2012 Winter;24 :E39-40. doi: 10.1176/appi.neuropsych.11020045. 17 years of treatment-resistant mutism in non-catatonic, childhood-onset schizophrenia: a rare case report. Khairkar P, Jain V, Bhatnagar A, Saoji N. | Case Report |
| 136. | Schizophr Res. 2012 Jul;138(2-3):150-6. doi: 10.1016/j.schres.2012.02.016. Epub 2012 Mar 10. White matter integrity, language, and childhood onset schizophrenia. Clark K , Narr KL, O'Neill J, Levitt J, Siddarth P, Phillips O, Toga A, Caplan R. | No Clinical |
| 137. | Cereb Cortex. 2013 Jan;23 :127-38. doi: 10.1093/cercor/bhr388. Epub 2012 Jan 23. The anatomical distance of functional connections predicts brain network topology in health and schizophrenia. Alexander-Bloch AF , Vértes PE, Stidd R, Lalonde F, Clasen L, Rapoport J, Giedd J, Bullmore ET, Gogtay N. | No Clinical |
| 138. | Mol Cytogenet. 2012 Jan 3;5 :2. doi: 10.1186/1755-8166-5-2. Isochromosome 13 in a patient with childhood-onset schizophrenia, ADHD, and motor tic disorder. Graw SL , Swisshelm K, Floyd K, Carstens BJ, Wamboldt MZ, Ross RG, Leonard S. | Case Report |
| 139. | Arch Gen Psychiatry. 2012 Jan;69 :16-26. doi: 10.1001/archgenpsychiatry.2011.150. Progressive brain changes in children and adolescents with first-episode psychosis. Arango C , Rapado-Castro M, Reig S, Castro-Fornieles J, González-Pinto A, Otero S, Baeza I, Moreno C, Graell M, Janssen J, Parellada M, Moreno D, Bargalló N, Desco M. | No Clinical |
| 140. | Neuroimage. 2012 Feb 15;59 :3889-900. doi: 10.1016/j.neuroimage.2011.11.035. Epub 2011 Nov 18. The discovery of population differences in network community structure: new methods and applications to brain functional networks in schizophrenia. Alexander-Bloch A , Lambiotte R, Roberts B, Giedd J, Gogtay N, Bullmore E. | No Clinical |
| 141. | Brain Stimul. 2011 Oct;4 :275-80. doi: 10.1016/j.brs.2011.01.001. Epub 2011 Feb 1. Tolerability of transcranial direct current stimulation in childhood-onset schizophrenia. Mattai A , Miller R, Weisinger B, Greenstein D, Bakalar J, Tossell J, David C, Wassermann EM, Rapoport J, Gogtay N. | No Clinical |
| 142. | Seishin Shinkeigaku Zasshi. 2011;113 :696-703. Childhood-onset schizophrenia and schizophrenia spectrum disorder Matsumoto H . | No English |
| 143. | Schizophr Res. 2011 Dec;133(1-3):82-90. doi: 10.1016/j.schres.2011.07.011. Epub 2011 Aug 27. Proton magnetic resonance spectroscopy and thought disorder in childhood schizophrenia. Seese RR , O'Neill J, Hudkins M, Siddarth P, Levitt J, Tseng B, Wu KN, Caplan R. | No Clinical |
| 144. | Psychiatry Res. 2011 Sep 30;193 :131-7. doi: 10.1016/j.pscychresns.2011.02.010. Epub 2011 Jul 30.Cerebellar development in childhood onset schizophrenia and non-psychotic siblings. Greenstein D , Lenroot R, Clausen L, Chavez A, Vaituzis AC, Tran L, Gogtay N, Rapoport J. | No Clinical |
| 145. | Curr Psychiatry Rep. 2011 Oct;13 :321-2. doi: 10.1007/s11920-011-0212-4. Childhood-onset schizophrenia: the challenge of diagnosis. Gochman P , Miller R, Rapoport JL. | Letter |
| 146. | J Am Acad Child Adolesc Psychiatry. 2011 Jul;50 :697-704. doi: 10.1016/j.jaac.2011.03.016. Epub 2011 Jun 11. Normalization of cortical gray matter deficits in nonpsychotic siblings of patients with childhood-onset schizophrenia. Mattai AA , Weisinger B, Greenstein D, Stidd R, Clasen L, Miller R, Tossell JW, Rapoport JL, Gogtay N. | No Clinical |
| **147.** | **J Am Acad Child Adolesc Psychiatry. 2011 Jul;50 :681-686.e3. doi: 10.1016/j.jaac.2011.03.020. Epub 2011 Jun 11. Childhood onset schizophrenia: high rate of visual hallucinations. David CN , Greenstein D, Clasen L, Gochman P, Miller R, Tossell JW, Mattai AA, Gogtay N, Rapoport JL.** | **Included** |
| 148. | Autism. 2011 Nov;15 :713-27. doi: 10.1177/1362361310396383. Epub 2011 Jun 20. Validation of the Autism Spectrum Screening Questionnaire, Mandarin Chinese Version (CH-ASSQ) in Beijing, China. Guo YQ , Tang Y, Rice C, Lee LC, Wang YF, Cubells JF. | Unrelated |
| 149. | Neuroimage. 2011 Aug 15;57 :1517-23. doi: 10.1016/j.neuroimage.2011.05.032. Epub 2011 May 15.Catechol-o-methyl transferase (COMT) val158met polymorphism and adolescent cortical development in patients with childhood-onset schizophrenia, their non-psychotic siblings, and healthy controls. Raznahan A , Greenstein D, Lee Y, Long R, Clasen L, Gochman P, Addington A, Giedd JN, Rapoport JL, Gogtay N. | No Clinical |
| 150. | Schizophr Bull. 2013 Jan;39 :52-8. doi: 10.1093/schbul/sbr049. Epub 2011 May 25. Lack of gender influence on cortical and subcortical gray matter development in childhood-onset schizophrenia. Weisinger B , Greenstein D, Mattai A, Clasen L, Lalonde F, Feldman S, Miller R, Tossell JW, Vyas NS, Stidd R, David C, Gogtay N. | No Clinical |
| 151. | Schizophr Bull. 2011 May;37 :504-13. doi: 10.1093/schbul/sbr030. Age of onset of schizophrenia: perspectives from structural neuroimaging studies. Gogtay N , Vyas NS, Testa R, Wood SJ, Pantelis C. | No Clinical |
| 152. | BMC Psychiatry. 2011 Apr 14;11:60. doi: 10.1186/1471-244X-11-60. Familial liability, obstetric complications and childhood development abnormalities in early onset schizophrenia: a case control study. Margari F , Petruzzelli MG, Lecce PA, Todarello O, De Giacomo A, Lucarelli E, Martinelli D, Margari L. | Case Report |
| 153. | Drugs. 2011 Jan 22;71 :179-208. doi: 10.2165/11585350-000000000-00000. Management of schizophrenia in children and adolescents: focus on pharmacotherapy. Masi G , Liboni F. | No Clinical |
| 154. | Am J Psychiatry. 2011 Apr;168 :427-35. doi: 10.1176/appi.ajp.2010.10050681. Epub 2011 Jan 18. Hippocampal volume development in healthy siblings of childhood-onset schizophrenia patients. Mattai A , Hosanagar A, Weisinger B, Greenstein D, Stidd R, Clasen L, Lalonde F, Rapoport J, Gogtay N | No Clinical |
| 155. | Case Rep Genet. 2011;2011:585893. doi: 10.1155/2011/585893. Epub 2011 Sep 12. A Novel Microduplication in the Neurodevelopmental Gene SRGAP3 That Segregates with Psychotic Illness in the Family of a COS Proband. Wilson NK , Lee Y, Long R, Hermetz K, Rudd MK, Miller R, Rapoport JL, Addington AM. | Case Report |
| 156. | Schizophr Res Treatment. 2011;2011:581686. doi: 10.1155/2011/581686. Epub 2011 Oct 16. Parietal lobes in schizophrenia: do they matter? Yildiz M , Borgwardt SJ, Berger GE. | No Clinical |
| 157. | Neuroimage. 2011 Mar 15;55 :491-9. doi: 10.1016/j.neuroimage.2010.12.041. Epub 2010 Dec 22.Neuroanatomy in adolescents and young adults with 22q11 deletion syndrome: comparison to an IQ-matched group. Baker K , Chaddock CA, Baldeweg T, Skuse D. | No VEOS |
| 158. | J Clin Psychiatry. 2010 Nov;71 :e29. doi: 10.4088/JCP.9101tx4c. Symptomatic presentation and initial treatment for schizophrenia in children and adolescents. Correll CU . | Review |
| 159. | Front Syst Neurosci. 2010 Oct 8;4:147. doi: 10.3389/fnsys.2010.00147. eCollection 2010. Disrupted modularity and local connectivity of brain functional networks in childhood-onset schizophrenia. Alexander-Bloch AF , Gogtay N, Meunier D, Birn R, Clasen L, Lalonde F, Lenroot R, Giedd J, Bullmore ET. | No Clinical |
| **160.** | **Early Interv Psychiatry. 2010 Nov;4 :305-13. doi: 10.1111/j.1751-7893.2010.00204.x. Verbal and visuospatial working memory development and deficits in children and adolescents with schizophrenia. White T , Schmidt M, Karatekin C.** | **Included** |
| 161. | Int J Dev Neurosci. 2011 May;29 :251-8. doi: 10.1016/j.ijdevneu.2010.10.003. Epub 2010 Oct 16. Childhood onset schizophrenia: support for a progressive neurodevelopmental disorder. Rapoport JL , Gogtay N. | No Clinical |
| 162. | J Am Acad Child Adolesc Psychiatry. 2010 Aug;49 :736-51; quiz 856-7. doi: 10.1016/j.jaac.2010.03.016. Epub 2010 May 26. Progress in cytogenetics: implications for child psychopathology. Hoffman EJ , State MW. | Review |
| 163. | Hum Brain Mapp. 2010 Jun;31 :917-25. doi: 10.1002/hbm.21028. Childhood psychiatric disorders as anomalies in neurodevelopmental trajectories. Shaw P , Gogtay N, Rapoport J. | No Clinical |
| 164. | Mol Psychiatry. 2011 Mar;16 :238-9. doi: 10.1038/mp.2010.59. Epub 2010 May 18. A novel frameshift mutation in UPF3B identified in brothers affected with childhood onset schizophrenia and autism spectrum disorders. Addington AM, Gauthier J, Piton A, Hamdan FF, Raymond A, Gogtay N, Miller R, Tossell J, Bakalar J, Inoff-Germain G, Gochman P, Long R, Rapoport JL, Rouleau GA. | Case Report |
| 165. | Schizophr Res. 2010 Jul;120(1-3):84-6. doi: 10.1016/j.schres.2009.12.032. Epub 2010 May 8. Asymmetry loss is local rather than global in adolescent onset schizophrenia. Clark GM , Crow TJ, Barrick TR, Collinson SL, James AC, Roberts N, Mackay CE. | No Clinical |
| 166. | J Neurolinguistics. 2010 May;23 :204-222. doi: 10.1016/j.jneuroling.2009.07.004. Semantic Processing and Thought Disorder in Childhood-Onset Schizophrenia: Insights from fMRI. Borofsky LA , McNealy K, Siddarth P, Wu KN, Dapretto M, Caplan R. | No Clinical |
| 167. | Prog Neuropsychopharmacol Biol Psychiatry. 2010 May 30;34 :728-9. doi: 10.1016/j.pnpbp.2010.03.028. Epub 2010 Mar 27. Amisulpride-induced tardive dyskinesia in childhood onset schizophrenia. Goyal N, Sinha VK. | No Clinical |
| 168. | J Psychosom Res. 2009 Dec;67 :515-23. doi: 10.1016/j.jpsychores.2009.08.002. Tourette syndrome and comorbid early-onset schizophrenia. Kerbeshian J , Peng CZ, Burd L. | Lumping |
| 169. | Schizophr Res. 2010 Jan;116 :44-8. doi: 10.1016/j.schres.2009.10.018. Effects of clozapine and olanzapine on cortical thickness in childhood-onset schizophrenia. Mattai A , Chavez A, Greenstein D, Clasen L, Bakalar J, Stidd R, Rapoport J, Gogtay N. | No Clinical |
| 170. | Fortschr Neurol Psychiatr. 2009 Oct;77 :558-67. doi: 10.1055/s-0028-1109737. Epub 2009 Oct 9. Early development of childhood-onset schizophrenia. Eggers C , Bunk D. | No English |
| 171. | Hum Psychopharmacol. 2009 Oct;24 :584-9. doi: 10.1002/hup.1056. Adjunctive use of lithium carbonate for the management of neutropenia in clozapine-treated children. Mattai A , Fung L, Bakalar J, Overman G, Tossell J, Miller R, Rapoport J, Gogtay N. | No Clinical |
| 172. | Schizophr Res. 2009 Nov;115 :12-6. doi: 10.1016/j.schres.2009.07.026. Epub 2009 Sep 5. General absence of abnormal cortical asymmetry in childhood-onset schizophrenia: a longitudinal study.Bakalar JL , Greenstein DK, Clasen L, Tossell JW, Miller R, Evans AC, Mattai AA, Rapoport JL, Gogtay N. | No Clinical |
| 173. | Afr J Psychiatry (Johannesbg). 2009 May;12 :144-8. doi: 10.4314/ajpsy.v12i2.43732. Clinical characteristics and premorbid variables in childhood-onset schizophrenia: a descriptive study of twelve cases from a schizophrenia founder population. Maydell RJ , van der Walt C, Roos JL, Scribante L, Ladikos A. | No Data |
| 174. | Encephale. 2009 Jan;35 Suppl 1:S6-9. doi: 10.1016/S0013-7006(09)75526-5. Schizophrenia in childhood Da Fonseca D . | Review |
| 175. | Curr Psychiatry Rep. 2009 Apr;11 :156-61. doi: 10.1007/s11920-009-0024-y. The genetics of childhood-onset schizophrenia: when madness strikes the prepubescent. Addington AM , Rapoport JL. | No Clinical |
| 176. | J Am Acad Child Adolesc Psychiatry. 2009 Jan;48 :10-8. doi: 10.1097/CHI.0b013e31818b1c63. Autism spectrum disorders and childhood-onset schizophrenia: clinical and biological contributions to a relation revisited. Rapoport J , Chavez A, Greenstein D, Addington A, Gogtay N. | Review |
| 177. | Ann N Y Acad Sci. 2009 Jan;1151:85-101. doi: 10.1111/j.1749-6632.2008.03453.x. Clinical characteristics of an Afrikaner founder population recruited for a schizophrenia genetic study. Roos JL , Pretorius HW, Karayiorgou M. | No English |
| 178. | Neuropsychiatr Dis Treat. 2008 Aug;4 :825-30. doi: 10.2147/ndt.s2484. Very early onset and greater vulnerability in schizophrenia: A clinical and neuroimaging study. Margari F , Presicci A, Petruzzelli MG, Ventura P, Di Cuonzo F, Palma M, Margari L. | Case Report |
| 179. | Proc Natl Acad Sci U S A. 2008 Oct 14;105(41):15979-84. doi: 10.1073/pnas.0806485105. Epub 2008 Oct 13. Three-dimensional brain growth abnormalities in childhood-onset schizophrenia visualized by using tensor-based morphometry. Gogtay N , Lu A, Leow AD, Klunder AD, Lee AD, Chavez A, Greenstein D, Giedd JN, Toga AW, Rapoport JL, Thompson PM. | No Clinical |
| 180. | Brain Cogn. 2008 Dec;68 :391-414. doi: 10.1016/j.bandc.2008.08.025. Epub 2008 Oct 2. A review on eye movement studies in childhood and adolescent psychiatry. Rommelse NN , Van der Stigchel S, Sergeant JA. | Review |
| 181. | J Am Acad Child Adolesc Psychiatry. 2008 Nov;47 :1233-51. doi: 10.1097/CHI.0b013e318185e703.Neuroimaging studies of normal brain development and their relevance for understanding childhood neuropsychiatric disorders. Marsh R , Gerber AJ, Peterson BS. | No Clinical |
| 182. | J Am Acad Child Adolesc Psychiatry. 2008 Oct;47 :1120-4. doi: 10.1097/CHI.0b013e31817eed7a. Epub 2009 Aug 21. Childhood-onset schizophrenia: insights from neuroimaging studies. Gogtay N , Rapoport JL. | No Clinical |
| 183. | Hum Psychopharmacol. 2008 Dec;23 :715-22. doi: 10.1002/hup.982. Long-term sustained benefits of clozapine treatment in refractory early onset schizophrenia: a retrospective study in Korean children and adolescents. Kim Y , Kim BN, Cho SC, Kim JW, Shin MS. | No Clinical |
| 184. | Mol Psychiatry. 2008 Oct;13 :910-1. doi: 10.1038/mp.2008.67. Sex chromosome anomalies in childhood onset schizophrenia: an update. Eckstrand K, Addington AM, Stromberg T, Merriman B, Miller R, Gochman P, Long R, Dutra A, Chen Z, Meltzer P, Nelson SF, Rapoport JL. | No Clinical |
| 185. | Neuropsychologia. 2009 Jan;47 :132-8. doi: 10.1016/j.neuropsychologia.2008.08.006. Epub 2008 Aug 13. Clinical practice of rTMS reveals a functional dissociation between agency and hallucinations in schizophrenia. Jardri R , Delevoye-Turrell Y, Lucas B, Pins D, Bulot V, Delmaire C, Thomas P, Delion P, Goeb JL. | Case Report |
| 186. | J Am Acad Child Adolesc Psychiatry. 2008 Oct;47 :1133-40. doi: 10.1097/CHI.0b013e3181825b0c. Remission status and cortical thickness in childhood-onset schizophrenia. Greenstein DK , Wolfe S, Gochman P, Rapoport JL, Gogtay N. | No Clinical |
| 187. | Expert Opin Pharmacother. 2008 Aug;9 :2053-68. doi: 10.1517/14656566.9.12.2053. Child and adolescent schizophrenia: pharmacological approaches. Madaan V , Dvir Y, Wilson DR. | Review |
| 188. | Novartis Found Symp. 2008;289:101-12; discussion 112-8, 193-5. doi: 10.1002/9780470751251.ch9. Trajectories of anatomic brain development as a phenotype. Giedd JN , Lenroot RK, Shaw P, Lalonde F, Celano M, White S, Tossell J, Addington A, Gogtay N. | No Clinical |
| 189. | Eur Psychiatry. 2008 Aug;23 :331-5. doi: 10.1016/j.eurpsy.2008.03.005. Epub 2008 May 2. Association between age at onset and clinical features of schizophrenia: the Northern Finland 1966 birth cohort study. Luoma S , Hakko H, Ollinen T, Järvelin MR, Lindeman S. | No VEOS |
| 190. | Clin Child Psychol Psychiatry. 2008 Jan;13 :81-94. doi: 10.1177/1359104507086343. Use of the ADOS and ADI-R in children with psychosis: importance of clinical judgment. Reaven JA , Hepburn SL, Ross RG. | No VEOS |
| 191. | Science. 2008 Apr 25;320(5875):539-43. doi: 10.1126/science.1155174. Epub 2008 Mar 27. Rare structural variants disrupt multiple genes in neurodevelopmental pathways in schizophrenia. Walsh T , McClellan JM, McCarthy SE, Addington AM, Pierce SB, Cooper GM, Nord AS, Kusenda M, Malhotra D, Bhandari A, Stray SM, Rippey CF, Roccanova P, Makarov V, Lakshmi B, Findling RL, Sikich L, Stromberg T, Merriman B, Gogtay N, Butler P, Eckstrand K, Noory L, Gochman P, Long R, Chen Z, Davis S, Baker C, Eichler EE, Meltzer PS, Nelson SF, Singleton AB, Lee MK, Rapoport JL, King MC, Sebat J. | No Clinical |
| 192. | Arq Neuropsiquiatr. 2007 Dec;65(4B):1216-9. doi: 10.1590/s0004-282x2007000700024. Total agenesis of the corpus callosum in a patient with childhood-onset schizophrenia. Hallak JE , Crippa JA, Pinto JP, Machado de Sousa JP, Trzesniak C, Dursun SM, McGuire P, Deakin JF, Zuardi AW. | No Clinical |
| 193. | Psychiatry Res. 2008 Apr 15;158 :356-62. doi: 10.1016/j.psychres.2006.09.006. Epub 2008 Feb 12. Does catatonia influence the phenomenology of childhood onset schizophrenia beyond motor symptoms? Bonnot O , Tanguy ML, Consoli A, Cornic F, Graindorge C, Laurent C, Tordjman S, Cohen D. | No Veos |
| 194. | Schizophr Bull. 2008 Mar;34 :341-53. doi: 10.1093/schbul/sbm157. Epub 2008 Jan 29. Longitudinal brain changes in early-onset psychosis. Arango C , Moreno C, Martínez S, Parellada M, Desco M, Moreno D, Fraguas D, Gogtay N, James A, Rapoport J | No Clinical |
| 195. | Expert Opin Pharmacother. 2008 Feb;9 :459-65. doi: 10.1517/14656566.9.3.459. Clozapine use in children and adolescents. Gogtay N , Rapoport J. | No Clinical |
| 196. | Clin Child Psychol Psychiatry. 2007 Oct;12 :537-48. doi: 10.1177/1359104507078476. 'All that glitters is not gold': misdiagnosis of psychosis in pervasive developmental disorders--a case series. Dossetor DR . | Case Report |
| 197. | Schizophr Bull. 2008 Jan;34 :30-6. doi: 10.1093/schbul/sbm103. Epub 2007 Sep 29. Cortical brain development in schizophrenia: insights from neuroimaging studies in childhood-onset schizophrenia. Gogtay N . | No Clinical |
| 198. | J Am Acad Child Adolesc Psychiatry. 2007 Oct;46 :1349-1356. doi: 10.1097/chi.0b013e31812eed10. Clozapine treatment of childhood-onset schizophrenia: evaluation of effectiveness, adverse effects, and long-term outcome. Sporn AL , Vermani A , Greenstein DK , Bobb AJ , Spencer EP , ClasenLS , Tossell JW , Stayer CC , Gochman PA , Lenane MC , Rapoport JL , Gogtay N . | No Clinical |
| 199. | J Autism Dev Disord. 2008 Apr;38 :668-77. doi: 10.1007/s10803-007-0435-8. Epub 2007 Sep 19. Sequencing and analyzing the t(1;7) reciprocal translocation breakpoints associated with a case of childhood-onset schizophrenia/autistic disorder. Idol JR , Addington AM, Long RT, Rapoport JL, Green ED. | No Clinical |
| 200. | Neuropsychopharmacology. 2008 Jan;33 :181-97. doi: 10.1038/sj.npp.1301553. Epub 2007 Sep 12.Brain neuroplasticity in healthy, hyperactive and psychotic children: insights from neuroimaging. Rapoport JL , Gogtay N. | No Clinical |
| 201 . | J Child Psychol Psychiatry. 2007 Sep;48 :852-62. doi: 10.1111/j.1469-7610.2007.01747.x. Dynamic mapping of cortical development before and after the onset of pediatric bipolar illness. Gogtay N , Ordonez A, Herman DH, Hayashi KM, Greenstein D, Vaituzis C, Lenane M, Clasen L, Sharp W, Giedd JN, Jung D, Nugent TF 3rd, Toga AW, Leibenluft E, Thompson PM, Rapoport JL. | No Clinical |
| 202. | Schizophr Bull. 2007 Sep;33 :1082-3. doi: 10.1093/schbul/sbm080. Epub 2007 Aug 1. Antipsychotic medication for childhood-onset schizophrenia. Kennedy E , Kumar A, Datta SS. | No Clinical |
| 203. | Cochrane Database Syst Rev. 2007 Jul 18;2007 :CD004027. doi: 10.1002/14651858.CD004027.pub2.Antipsychotic medication for childhood-onset schizophrenia. Kennedy E , Kumar A, Datta SS. | Review |
| 204. | Arch Gen Psychiatry. 2007 Jul;64 :772-80. doi: 10.1001/archpsyc.64.7.772. Cortical brain development in nonpsychotic siblings of patients with childhood-onset schizophrenia. Gogtay N , Greenstein D, Lenane M, Clasen L, Sharp W, Gochman P, Butler P, Evans A, Rapoport J. | No Clinical |
| 205. | J Child Psychol Psychiatry. 2007 May;48 :415-35. doi: 10.1111/j.1469-7610.2006.01681.x. Annotation: what electrical brain activity tells us about brain function that other techniques cannot tell us - a child psychiatric perspective. Banaschewski T , Brandeis D. | No Clinical |
| 206. | Schizophr Res. 2007 Feb;90(1-3):62-70. doi: 10.1016/j.schres.2006.10.014. Epub 2006 Dec 11. Dynamic mapping of hippocampal development in childhood onset schizophrenia. Nugent TF 3rd , Herman DH, Ordonez A, Greenstein D, Hayashi KM, Lenane M, Clasen L, Jung D, Toga AW, Giedd JN, Rapoport JL, Thompson PM, Gogtay N. | No Clinical |
| 207. | J Child Psychol Psychiatry. 2006 Oct;47 :1003-12. doi: 10.1111/j.1469-7610.2006.01658.x. Childhood onset schizophrenia: cortical brain abnormalities as young adults. Greenstein D , Lerch J, Shaw P, Clasen L, Giedd J, Gochman P, Rapoport J, Gogtay N. | No Clinical |
| 208. | Mol Psychiatry. 2007 Feb;12 :195-205. doi: 10.1038/sj.mp.4001906. Epub 2006 Oct 10. Neuregulin 1 (8p12) and childhood-onset schizophrenia: susceptibility haplotypes for diagnosis and brain developmental trajectories. Addington AM , Gornick MC, Shaw P, Seal J, Gogtay N, Greenstein D, Clasen L, Coffey M, Gochman P, Long R, Rapoport JL. | No Clinical |
| 209. | Indian J Psychiatry. 2006 Oct;48 :215-22. doi: 10.4103/0019-5545.31552. Study of childhood onset schizophrenia (COS) using SPECT and neuropsychological assessment. Malhotra S , Gupta N, Bhattacharya A, Kapoor M. | No VEOS |
| 210. | CNS Drugs. 2006;20 :841-66. doi: 10.2165/00023210-200620100-00005. Children with schizophrenia: clinical picture and pharmacological treatment. Masi G , Mucci M, Pari C. | Review |
| 211. | J Child Adolesc Psychopharmacol. 2006 Aug;16 :393-403. doi: 10.1089/cap.2006.16.393. An open-label randomized comparison of olanzapine versus risperidone in the treatment of childhood-onset schizophrenia. Mozes T , Ebert T, Michal SE, Spivak B, Weizman A. | No Clinical |
| 212. | Schizophr Res. 2006 Dec;88(1-3):90-5. doi: 10.1016/j.schres.2006.07.006. Epub 2006 Aug 17. High rates of comorbidity are found in childhood-onset schizophrenia. Ross RG , Heinlein S, Tregellas H. | No Data |
| 213. | J Neural Transm (Vienna). 2007;114 :505-12. doi: 10.1007/s00702-006-0553-z. Epub 2006 Aug 10. Forty-two-years later: the outcome of childhood-onset schizophrenia. Remschmidt H , Martin M, Fleischhaker C, Theisen FM, Hennighausen K, Gutenbrunner C, Schulz E. | No VEOS |
| 214. | Arch Gen Psychiatry. 2006 Jul;63 :721-30. doi: 10.1001/archpsyc.63.7.721. Childhood-onset schizophrenia: A double-blind, randomized clozapine-olanzapine comparison. Shaw P , Sporn A, Gogtay N, Overman GP, Greenstein D, Gochman P, Tossell JW, Lenane M, Rapoport JL. | No Clinical |
| 215. | J Med Genet. 2006 Nov;43 :887-92. doi: 10.1136/jmg.2006.043380. Epub 2006 Jun 8. Segmental uniparental isodisomy on 5q32-qter in a patient with childhood-onset schizophrenia. Seal JL, Gornick MC, Gogtay N, Shaw P, Greenstein DK, Coffey M, Gochman PA, Stromberg T, Chen Z, Merriman B, Nelson SF, Brooks J, Arepalli S, Wavrant-De Vrièze F, Hardy J, Rapoport JL, Addington AM. | No Clinical |
| **216.** | **Schizophr Res. 2006 Sep;86(1-3):123-9. doi: 10.1016/j.schres.2006.04.020. Epub 2006 May 30. Sleep disturbances in childhood-onset schizophrenia. Mattai AA , Tossell J, Greenstein DK, Addington A, Clasen LS, Gornick MC, Seal J, Inoff-Germain G, Gochman PA, Lenane M, Rapoport JL, Gogtay N.** | **Included** |
| **217.** | **Eur Child Adolesc Psychiatry. 2006 Sep;15 :360-6. doi: 10.1007/s00787-006-0542-7. Epub 2006 Apr 8. Comparative study of neuropsychological correlates in schizophrenia with onset in childhood, adolescence and adulthood. Biswas P , Malhotra S, Malhotra A, Gupta N.** | **Included** |
| 218. | Am J Med Genet B Neuropsychiatr Genet. 2006 Mar 5;141B :192. doi: 10.1002/ajmg.b.30263. Hyperprolinemia is not associated with childhood onset schizophrenia. Jacquet H , Rapoport JL, Hecketsweiler B, Bobb A, Thibaut F, Frébourg T, Campion D. | No Clinical |
| 219. | Arch Gen Psychiatry. 2006 Jan;63 :25-34. doi: 10.1001/archpsyc.63.1.25. Dynamically spreading frontal and cingulate deficits mapped in adolescents with schizophrenia. Vidal CN , Rapoport JL, Hayashi KM, Geaga JA, Sui Y, McLemore LE, Alaghband Y, Giedd JN, Gochman P, Blumenthal J, Gogtay N, Nicolson R, Toga AW, Thompson PM. | No Clinical |
| 220. | Child Adolesc Psychiatr Clin N Am. 2006 Jan;15 :109-33. doi: 10.1016/j.chc.2005.08.011. The schizophrenia prodrome: a developmentally informed review and update for psychopharmacologic treatment. Thomas LE , Woods SW. | Review |
| 221. | J Child Psychol Psychiatry. 2005 Dec;46 :1354-62. doi: 10.1111/j.1469-7610.2005.01437.x. Saccadic eye movement task identifies cognitive deficits in children with schizophrenia, but not in unaffected child relatives. Ross RG , Heinlein S, Zerbe GO, Radant A. | No Clinical |
| 222. | Eur Child Adolesc Psychiatry. 2005 Sep;14 :341-50. doi: 10.1007/s00787-005-0483-6. Early-onset schizophrenia: a 15-year follow-up. Röpcke B , Eggers C. | No VEOS |
| 223. | J Am Acad Child Adolesc Psychiatry. 2005 Sep;44 :925-33. doi: 10.1097/01.chi.0000170552.15798.dd. Hormonal correlates of clozapine-induced weight gain in psychotic children: an exploratory study. Sporn AL , Bobb AJ, Gogtay N, Stevens H, Greenstein DK, Clasen LS, Tossell JW, Nugent T, Gochman PA, Sharp WS, Mattai A, Lenane MC, Yanovski JA, Rapoport JL. | No Clinical |
| 224. | J Child Adolesc Psychopharmacol. 2005 Jun;15 :395-402. doi: 10.1089/cap.2005.15.395. Childhood-onset schizotypal disorder: a follow-up study and comparison with childhood-onset schizophrenia. Asarnow JR . | No English |
| 225. | Biol Psychiatry. 2005 Jul 1;58 :10-5. doi: 10.1016/j.biopsych.2005.02.009. Lack of evidence for elevated obstetric complications in childhood onset schizophrenia. Ordoñez AE , Bobb A, Greenstein D, Baker N, Sporn A, Lenane M, Malaspina D, Rapaport J, Gogtay N. | No Clinical |
| 226. | Schizophr Res. 2005 Sep 15;77(2-3):271-7. doi: 10.1016/j.schres.2005.04.002. IQ stabilization in childhood-onset schizophrenia. Gochman PA , Greenstein D, Sporn A, Gogtay N, Keller B, Shaw P, Rapoport JL. | No VEOS |
| 227. | Psychiatry Res. 2005 Apr 30;138 :221-33. doi: 10.1016/j.pscychresns.2005.01.001. Abnormal development of the anterior cingulate in childhood-onset schizophrenia: a preliminary quantitative MRI study. Marquardt RK , Levitt JG, Blanton RE, Caplan R, Asarnow R, Siddarth P, Fadale D, McCracken JT, Toga AW. | No Clinical |
| 228. | Expert Rev Neurother. 2004 Jan;4 :53-60. doi: 10.1586/14737175.4.1.53. Pharmacologic treatment of adolescent and child schizophrenia. Young CM , Findling RL. | No Clinical |
| 229. | Encephale. 2004 Nov-Dec;30 :540-7. doi: 10.1016/s0013-7006(04)95468-1. [A rare and not very studied disorder: childhood-onset schizophrenia. A case report]. Bailly D , de Chouly de Lenclave MB. | Case Report |
| 230. | Arch Pediatr. 2005 Feb;12 :176-9. doi: 10.1016/j.arcped.2004.11.019. Fragile X syndrome and very early onset schizophrenia: a female case study Vantalon V , Briard-Luginbuhl V, Mouren MC. | Case Report |
| 231. | Behav Genet. 2005 Mar;35 :159-75. doi: 10.1007/s10519-004-1016-7. MMPI vulnerability indicators for schizophrenia and attention deficit disorder: UCLA family study of biological parents of offspring with childhood-onset schizophrenia or ADHD. Subotnik KL , Asarnow RF, Nuechterlein KH, Fogelson DL, Thorpe TI, Payne DL, Giannini CA, Kuppinger HE, Torquato RD, Mintz J, Hwang SS, Gottesman II. | Unrelated |
| 232. | Schizophr Res. 2005 Mar 1;73(2-3):243-52. doi: 10.1016/j.schres.2004.07.020. Childhood-onset schizophrenia: smooth pursuit eye-tracking dysfunction in family members. Sporn A , Greenstein D, Gogtay N, Sailer F, Hommer DW, Rawlings R, Nicolson R, Egan MF, Lenane M, Gochman P, Weinberger DR, Rapoport JL. | No Clinical |
| 233. | Schizophr Res. 2005 Mar 1;73(2-3):235-41. doi: 10.1016/j.schres.2004.07.023. Superior temporal gyrus differences in childhood-onset schizophrenia. Taylor JL , Blanton RE, Levitt JG, Caplan R, Nobel D, Toga AW. | No Clinical |
| 234. | J Child Adolesc Psychopharmacol. 2004 Fall;14 :448-54. doi: 10.1089/cap.2004.14.448. Stimulant drug treatment in childhood-onset schizophrenia with comorbid ADHD: an open-label case series. Tossell JW , Greenstein DK, Davidson AL, Job SB, Gochman P, Lenane M, Nugent Iii TF, Gogtay N, Sporn AL, Rapoport JL. | No Clinical |
| 235. | Drug Saf. 2004;27(14):1135-56. doi: 10.2165/00002018-200427140-00005. Benefit-risk assessment of atypical antipsychotics in the treatment of schizophrenia and comorbid disorders in children and adolescents. Toren P , Ratner S, Laor N, Weizman A. | No Clinical |
| 236. | Mol Psychiatry. 2005 Jun;10 :581-8. doi: 10.1038/sj.mp.4001599. GAD1 (2q31.1), which encodes glutamic acid decarboxylase (GAD67), is associated with childhood-onset schizophrenia and cortical gray matter volume loss. Addington AM , Gornick M, Duckworth J, Sporn A, Gogtay N, Bobb A, Greenstein D, Lenane M, Gochman P, Baker N, Balkissoon R, Vakkalanka RK, Weinberger DR, Rapoport JL, Straub RE. | No Clinical |
| 237. | Schizophr Res. 2004 Nov 1;71 :43-7. doi: 10.1016/j.schres.2004.01.012. Childhood onset schizophrenia: familial neurocognitive measures. Gochman PA , Greenstein D, Sporn A, Gogtay N, Nicolson R, Keller A, Lenane M, Brookner F, Rapoport JL. | No VEOS |
| 238. | J Am Acad Child Adolesc Psychiatry. 2004 Aug;43 :1026-9. doi: 10.1097/01.chi.0000127573.34038.e4. Looking for childhood schizophrenia: case series of false positives. Stayer C , Sporn A, Gogtay N, Tossell J, Lenane M, Gochman P, Rapoport JL. | Case Report |
| 239. | Am J Med Genet B Neuropsychiatr Genet. 2004 Jul 1;128B :24-6. doi: 10.1002/ajmg.b.30009. Childhood-onset schizophrenia and tryptophan hydroxylase gene polymorphism. Sekizawa T , Iwata Y, Nakamura K, Matsumoto H, Suzuki A, Suzuki K, Sekine Y, Takei N, Minabe Y, Mori N. | No VEOS |
| 240. | J Pediatr (Rio J). 2004 Apr;80(2 Suppl):S3-10. doi: 10.2223/1163. Functional psychosis in childhood and adolescence Tengan SK , Maia AK. | Review |
| 241. | Proc Natl Acad Sci U S A. 2004 May 25;101(21):8174-9. doi: 10.1073/pnas.0402680101. Epub 2004 May 17. Dynamic mapping of human cortical development during childhood through early adulthood. Gogtay N , Giedd JN, Lusk L, Hayashi KM, Greenstein D, Vaituzis AC, Nugent TF 3rd, Herman DH, Clasen LS, Toga AW, Rapoport JL, Thompson PM. | No Clinical |
| 242. | Biol Psychiatry. 2004 May 15;55 :989-94. doi: 10.1016/j.biopsych.2004.01.019. Pervasive developmental disorder and childhood-onset schizophrenia: comorbid disorder or a phenotypic variant of a very early onset illness? Sporn AL , Addington AM, Gogtay N, Ordoñez AE, Gornick M, Clasen L, Greenstein D, Tossell JW, Gochman P, Lenane M, Sharp WS, Straub RE, Rapoport JL. | Lumping |
| 243. | Biol Psychiatry. 2004 May 15;55 :976-80. doi: 10.1016/j.biopsych.2004.01.024. Polymorphisms in the 13q33.2 gene G72/G30 are associated with childhood-onset schizophrenia and psychosis not otherwise specified. Addington AM , Gornick M, Sporn AL, Gogtay N, Greenstein D, Lenane M, Gochman P, Baker N, Balkissoon R, Vakkalanka RK, Weinberger DR, Straub RE, Rapoport JL. | No Clinical |
| 244. | Neuroimage. 2004 Apr;21 :1781-9. doi: 10.1016/j.neuroimage.2003.11.005. 1H MRSI evidence of metabolic abnormalities in childhood-onset schizophrenia. O'Neill J , Levitt J, Caplan R, Asarnow R, McCracken JT, Toga AW, Alger JR. | No Clinical |
| 245. | J Child Psychol Psychiatry. 2004 Feb;45 :180-94. doi: 10.1111/j.1469-7610.2004.00213.x. Annotation: childhood-onset schizophrenia: clinical and treatment issues. Asarnow JR , Tompson MC, McGrath EP. | Review |
| 247. | Psychiatry Res. 2004 Jan 15;130 :43-55. doi: 10.1016/j.pscychresns.2003.10.001. Thought disorder and nucleus accumbens in childhood: a structural MRI study. Ballmaier M , Toga AW, Siddarth P, Blanton RE, Levitt JG, Lee M, Caplan R. | No Clinical |
| 248. | Arch Gen Psychiatry. 2004 Jan;61 :17-22. doi: 10.1001/archpsyc.61.1.17. Comparison of progressive cortical gray matter loss in childhood-onset schizophrenia with that in childhood-onset atypical psychoses. Gogtay N , Sporn A, Clasen LS, Nugent TF 3rd, Greenstein D, Nicolson R, Giedd JN, Lenane M, Gochman P, Evans A, Rapoport JL. | No Clinical |
| 249. | Mol Psychiatry. 2004 Mar;9 :225-6. doi: 10.1038/sj.mp.4001477. 22q11 deletion syndrome in childhood onset schizophrenia: an update. Sporn A, Addington A, Reiss AL, Dean M, Gogtay N, Potocnik U, Greenstein D, Hallmayer J, Gochman P, Lenane M, Baker N, Tossell J, Rapoport JL. | No VEOS |
| 250. | J Child Adolesc Psychopharmacol. 2003 Fall;13 :401-4. doi: 10.1089/104454603322572697. Clozapine-induced neutropenia in children: management with lithium carbonate. Sporn A , Gogtay N, Ortiz-Aguayo R, Alfaro C, Tossell J, Lenane M, Gochman P, Rapoport JL. | No VEOS |
| 251. | J Child Adolesc Psychopharmacol. 2003 Fall;13 :311-7. doi: 10.1089/104454603322572642. Olanzapine treatment in chronic drug-resistant childhood-onset schizophrenia: an open-label study. Mozes T , Greenberg Y, Spivak B, Tyano S, Weizman A, Mester R. | No Clinical |
| 252. | J Child Adolesc Psychopharmacol. 2003 Fall;13 :301-9. doi: 10.1089/104454603322572633. A 1-year open-label trial of olanzapine in school-age children with schizophrenia. Ross RG , Novins D, Farley GK, Adler LE. | No Clinical |
| 253. | Am J Psychiatry. 2003 Dec;160 :2181-9. doi: 10.1176/appi.ajp.160.12.2181. Progressive brain volume loss during adolescence in childhood-onset schizophrenia. Sporn AL , Greenstein DK, Gogtay N, Jeffries NO, Lenane M, Gochman P, Clasen LS, Blumenthal J, Giedd JN, Rapoport JL. | No Clinical |
| 254. | Psychiatr Serv. 2003 Nov;54 :1519-25. doi: 10.1176/appi.ps.54.11.1519. Pervasive developmental disorders among children and adolescents attending psychiatric day treatment. Sverd J , Dubey DR, Schweitzer R, Ninan R. | No VEOS |
| 255. | Br J Psychiatry. 2003 Nov;183:409-13. doi: 10.1192/bjp.183.5.409. Chromosome 22q11 deletions, velo-cardio-facial syndrome and early-onset psychosis. Molecular genetic study. Ivanov D , Kirov G, Norton N, Williams HJ, Williams NM, Nikolov I, Tzwetkova R, Stambolova SM, Murphy KC, Toncheva D, Thapar A, O'Donovan MC, Owen MJ. | No VEOS |
| 256. | Acta Neuropsychiatr. 2003 Jun;15 :140-7. doi: 10.1034/j.1601-5215.2003.00021.x. Magnetic resonance imaging studies on autism and childhood-onset schizophrenia in children and adolescents - a review. Lahuis B , Kemner C , Van Engeland H . | No Clinical |
| 257. | Schizophr Res. 2003 Jul 1;62(1-2):105-14. doi: 10.1016/s0920-9964(02)00354-7. Corpus callosum development in childhood-onset schizophrenia. Keller A , Jeffries NO, Blumenthal J, Clasen LS, Liu H, Giedd JN, Rapoport JL. | No Clinical |
| 258. | J Child Adolesc Psychopharmacol. 2002 Winter;12 :347-9. doi: 10.1089/104454602762599899. Clozapine-induced akathisia in children with schizophrenia. Gogtay N , Sporn A, Alfaro CL, Mulqueen A, Rapoport JL. | No Clinical |
| 259. | Am J Psychiatry. 2003 Mar;160 :569-71. doi: 10.1176/appi.ajp.160.3.569. Structural brain MRI abnormalities in healthy siblings of patients with childhood-onset schizophrenia. Gogtay N , Sporn A, Clasen LS, Greenstein D, Giedd JN, Lenane M, Gochman PA, Zijdenbos A, Rapoport JL. | No Clinical |
| 260. | Am J Psychiatry. 2003 Mar;160 :490-5. doi: 10.1176/appi.ajp.160.3.490. Parental schizophrenia spectrum disorders in childhood-onset and adult-onset schizophrenia. Nicolson R , Brookner FB, Lenane M, Gochman P, Ingraham LJ, Egan MF, Kendler KS, Pickar D, Weinberger DR, Rapoport JL. | No VEOS |
| 261. | J Clin Psychopharmacol. 2003 Feb;23 :87-91. doi: 10.1097/00004714-200302000-00012. Clozapine pharmacokinetics in children and adolescents with childhood-onset schizophrenia. Frazier JA , Cohen LG, Jacobsen L, Grothe D, Flood J, Baldessarini RJ, Piscitelli S, Kim GS, Rapoport JL. | No Clinical |
| 262. | Am J Psychiatry. 2003 Jan;160 :128-33. doi: 10.1176/appi.ajp.160.1.128. Progressive loss of cerebellar volume in childhood-onset schizophrenia. Keller A , Castellanos FX, Vaituzis AC, Jeffries NO, Giedd JN, Rapoport JL. | No Clinical |
| 263. | Arch Gen Psychiatry. 2002 Nov;59 :1053-60. doi: 10.1001/archpsyc.59.11.1053. Neurocognitive impairments in nonpsychotic parents of children with schizophrenia and attention-deficit/hyperactivity disorder: the University of California, Los Angeles Family Study. Asarnow RF , Nuechterlein KH, Subotnik KL, Fogelson DL, Torquato RD, Payne DL, Asamen J, Mintz J, Guthrie D. | No VEOS |
| 264. | Rev Prat. 2002 Jun 1;52 :1183-90. Presentations and diagnostic criteria of schizophrenia Corcos M , Clervoy P, Jeammet P. | Review |
| 265. | J Neural Transm (Vienna). 2002 Feb;109 :219-34. doi: 10.1007/s007020200019. Structural brain abnormalities specific to childhood-onset schizophrenia identified by neuroimaging techniques. Mehler C , Warnke A. | Review |
| 266. | J Am Acad Child Adolesc Psychiatry. 2002 May;41 :538-45. doi: 10.1097/00004583-200205000-00011. Childhood-onset schizophrenia: premorbid and prodromal diagnostic and treatment histories. Schaeffer JL , Ross RG. | No Data |
| 267. | Zh Nevrol Psikhiatr Im S S Korsakova. 2002;102 :9-12. The state with the predominance of obsessive-compulsive disorders in the structure of childhood onset schizophrenia Masikhina SN. | Lumping |
| 268. | Proc Natl Acad Sci U S A. 2002 Mar 19;99 :3717-22. doi: 10.1073/pnas.042700699. Epub 2002 Mar 12. Genetic variation at the 22q11 PRODH2/DGCR6 locus presents an unusual pattern and increases susceptibility to schizophrenia. Liu H , Heath SC, Sobin C, Roos JL, Galke BL, Blundell ML, Lenane M, Robertson B, Wijsman EM, Rapoport JL, Gogos JA, Karayiorgou M. | No VEOS |
| 269. | Can J Psychiatry. 2001 Dec;46 :965-8. doi: 10.1177/070674370104601010. Clozapine impact on clinical outcomes and aggression in severely ill adolescents with childhood-onset schizophrenia. Chalasani L , Kant R, Chengappa KN. | No Clinical |
| 270. | Can J Psychiatry. 2001 Dec;46 :923-30. doi: 10.1177/070674370104601004. Childhood-onset schizophrenia: research update. Kumra S , Shaw M, Merka P, Nakayama E, Augustin R. | Review |
| 271. | J Neural Transm (Vienna). 2002;109 :101-17. doi: 10.1007/s702-002-8240-3. Early-onset schizophrenia as a progressive-deteriorating developmental disorder: evidence from child psychiatry. Remschmidt H . | Review |
| 272. | J Neural Transm (Vienna). 2001;108 :1335-44. doi: 10.1007/s007020100010. A study of cranial computer tomograms in very early and early onset schizophrenia. Badura F , Trott GE, Mehler-Wex C, Scheuerpflug P, Hofmann E, Warmuth-Metz M, Nadjmi M, Solymosi L, Warnke A. | No Clinical |
| 273. | Psychiatry Res. 2001 Nov 5;108 :17-27. doi: 10.1016/s0925-4927(01)00108-1. Medial temporal lobe in childhood-onset schizophrenia. Levitt JG , Blanton RE, Caplan R, Asarnow R, Guthrie D, Toga AW,Capetillo-Cunliffe L, McCracken JT. | No Clinical |
| 274. | J Am Acad Child Adolesc Psychiatry. 2001 Oct;40 :1190-6. doi: 10.1097/00004583-200110000-00013. Differentiating childhood-onset schizophrenia from psychotic mood disorders. Calderoni D , Wudarsky M, Bhangoo R, Dell ML, Nicolson R, Hamburger SD, Gochman P, Lenane M, Rapoport JL, Leibenluft E. | No Clinical |
| 275. | Proc Natl Acad Sci U S A. 2001 Sep 25;98(20):11650-5. doi: 10.1073/pnas.201243998. Mapping adolescent brain change reveals dynamic wave of accelerated gray matter loss in very early-onset schizophrenia. Thompson PM , Vidal C, Giedd JN, Gochman P, Blumenthal J, Nicolson R, Toga AW, Rapoport JL | No Clinical |
| 276. | Am J Psychiatry. 2001 Aug;158 :1291-8. doi: 10.1176/appi.ajp.158.8.1291. Smooth pursuit eye-tracking impairment in childhood-onset psychotic disorders. Kumra S , Sporn A, Hommer DW, Nicolson R, Thaker G, Israel E, Lenane M, Bedwell J, Jacobsen LK, Gochman P, Rapoport JL. | No Clinical |
| 277. | Psychol Med. 2001 Jul;31 :907-14. doi: 10.1017/s0033291701003944. The association between obstetric complications and childhood-onset schizophrenia: a replication study. Matsumoto H , Takei N, Saito F, Kachi K, Mori N. | No VEOS |
| 278. | Aust N Z J Psychiatry. 2001 Jun;35 :272-81. doi: 10.1046/j.1440-1614.2001.00900.x. Imaging normal and abnormal brain development: new perspectives for child psychiatry. Rapoport JL , Castellanos FX, Gogate N, Janson K, Kohler S, Nelson P. | Review |
| 279. | Arch Gen Psychiatry. 2001 Jun;58 :581-8. doi: 10.1001/archpsyc.58.6.581. Schizophrenia and schizophrenia-spectrum personality disorders in the first-degree relatives of children with schizophrenia: the UCLA family study. Asarnow RF , Nuechterlein KH, Fogelson D, Subotnik KL, Payne DA, Russell AT, Asamen J, Kuppinger H, Kendler KS. | No VEOS |
| 280. | Z Kinder Jugendpsychiatr Psychother. 2001 May;29 :137-43. doi: 10.1024//1422-4917.29.2.137. [Agranulocytosis in a child with schizophrenia treated with clozapine--clinical findings and therapy, a case report]. Reitzle K , Warnke A, Wewetzer C, Müller H. | Case Report |
| 281. | Dialogues Clin Neurosci. 2001 Jun;3 :79-92. doi: 10.31887/DCNS.2001.3.2/hcourvoisie. Psychosis in children: diagnosis and treatment. Courvoisie H , Labellarte MJ, Riddle MA. | Review |
| 282. | Encephale. 2001 Jan-Feb;27 :45-50. [Microdeletion 22q11: apropos of case of schizophrenia in an adolescent]. Pinquier C , Héron D, de Carvalho W, Lazar G, Mazet P, Cohen D. | Review |
| 283. | Curr Psychiatry Rep. 2000 Oct;2 :410-5. doi: 10.1007/s11920-000-0024-4. Update on childhood-onset schizophrenia. Rapoport JL , Inoff-Germain G. | Review |
| 284 | Am J Med Genet. 2000 Dec 4;96 :749-53. doi: 10.1002/1096-8628(20001204)96:6<749::aid-ajmg10>3.0.co;2-k. Childhood-onset schizophrenia/autistic disorder and t(1;7) reciprocal translocation: identification of a BAC contig spanning the translocation breakpoint at 7q21. Yan WL , Guan XY, Green ED, Nicolson R, Yap TK, Zhang J, Jacobsen LK, Krasnewich DM, Kumra S, Lenane MC, Gochman P, Damschroder-Williams PJ, Esterling LE, Long RT, Martin BM, Sidransky E, Rapoport JL, Ginns EI. | Case Report |
| 285. | J Child Psychol Psychiatry. 2000 Sep;41 :679-94. MRI neuroimaging of childhood psychiatric disorders: a selective review. Eliez S , Reiss AL. | Review |
| 286. | Ment Retard Dev Disabil Res Rev. 2000;6 :180-5. doi: 10.1002/1098-2779(2000)6:3<180::AID-MRDD5>3.0.CO;2-I. Brain abnormalities observed in childhood-onset schizophrenia: a review of the structural magnetic resonance imaging literature. Sowell ER , Toga AW, Asarnow R. | Review |
| 287. | Am J Psychiatry. 2000 Sep;157 :1475-84. doi: 10.1176/appi.ajp.157.9.1475. Brain abnormalities in early-onset schizophrenia spectrum disorder observed with statistical parametric mapping of structural magnetic resonance images. Sowell ER , Levitt J, Thompson PM, Holmes CJ, Blanton RE, Kornsand DS, Caplan R, McCracken J, Asarnow R, Toga AW. | No Clinical |
| 288. | Am J Psychiatry. 2000 Sep;157 :1467-74. doi: 10.1176/appi.ajp.157.9.1467. Childhood-onset psychotic disorders: magnetic resonance imaging of volumetric differences in brain structure. Kumra S, Giedd JN, Vaituzis AC, Jacobsen LK, McKenna K, Bedwell J, Hamburger S, Nelson JE, Lenane M, Rapoport JL. | No Clinical |
| 289. | J Child Adolesc Psychopharmacol. 2000 Summer;10 :69-78. doi: 10.1089/cap.2000.10.69. Treatment of childhood-onset schizophrenia with olanzapine. Sholevar EH , Baron DA, Hardie TL. | No Clinical |
| 290. | J Am Acad Child Adolesc Psychiatry. 2000 Jul;39 :815-28. doi: 10.1097/00004583-200007000-00010. Review of neuroimaging studies of child and adolescent psychiatric disorders from the past 10 years. Hendren RL , De Backer I, Pandina GJ. | Review |
| **291.** | **J Am Acad Child Adolesc Psychiatry. 2000 Jun;39 :779-86. doi: 10.1097/00004583-200006000-00017. Childhood schizophrenia: responsiveness to questions during conversation. Abu-Akel A , Caplan R, Guthrie D, Komo S.** | **Included** |
| 292. | J Am Acad Child Adolesc Psychiatry. 2000 Jun;39 :771-8. doi: 10.1097/00004583-200006000-00016. Thought disorder in childhood schizophrenia: replication and update of concept. Caplan R , Guthrie D, Tang B, Komo S, Asarnow RF. | Lumping |
| 293. | J Autism Dev Disord. 2000 Feb;30 :29-38. doi: 10.1023/a:1005408010797. Schizophrenia with onset before the age of eleven: clinical characteristics of onset and course. Eggers C , Bunk D, Krause D. | No Data |
| 294. | Am J Psychiatry. 2000 May;157 :794-800. doi: 10.1176/appi.ajp.157.5.794. Premorbid speech and language impairments in childhood-onset schizophrenia: association with risk factors. Nicolson R , Lenane M, Singaracharlu S, Malaspina D, Giedd JN, Hamburger SD, Gochman P, Bedwell J, Thaker GK, Fernandez T, Wudarsky M, Hommer DW, Rapoport JL. | No VEOS |
| 295. | J Clin Psychopharmacol. 2000 Apr;20 :220-5. doi: 10.1097/00004714-200004000-00015. Olanzapine pharmacokinetics in pediatric and adolescent inpatients with childhood-onset schizophrenia. Grothe DR , Calis KA, Jacobsen L, Kumra S, DeVane CL, Rapoport JL, Bergstrom RF, Kurtz DL. | No Clinical |
| 296. | Schizophr Res. 2000 Apr 7;42 :135-44. doi: 10.1016/s0920-9964(99)00118-8. Neuropsychological deficits in pediatric patients with childhood-onset schizophrenia and psychotic disorder not otherwise specified. Kumra S , Wiggs E, Bedwell J, Smith AK, Arling E, Albus K, Hamburger SD, McKenna K, Jacobsen LK, Rapoport JL, Asarnow RF. | Lumping |
| 297. | Brain Res Brain Res Rev. 2000 Mar;31(2-3):147-56. doi: 10.1016/s0165-0173(99)00032-6. Lessons from childhood-onset schizophrenia. Nicolson R , Lenane M, Hamburger SD, Fernandez T, Bedwell J, Rapoport JL. | Review |
| 298. | J Child Adolesc Psychopharmacol. 1999;9 :239-45. doi: 10.1089/cap.1999.9.239. Elevated prolactin in pediatric patients on typical and atypical antipsychotics. Wudarsky M , Nicolson R, Hamburger SD, Spechler L, Gochman P, Bedwell J, Lenane MC, Rapoport JL. | No Clinical |
| 299. | J Am Acad Child Adolesc Psychiatry. 1999 Dec;38 :1536-43. doi: 10.1097/00004583-199912000-00015. Velocardiofacial syndrome in childhood-onset schizophrenia. Usiskin SI , Nicolson R, Krasnewich DM, Yan W, Lenane M, Wudarsky M, Hamburger SD, Rapoport JL. | No Clinical |
| 300. | Am J Psychiatry. 1999 Dec;156 :1996-7. doi: 10.1176/ajp.156.12.1996. Why does postpsychotic IQ decline in childhood-onset schizophrenia? Bedwell JS , Keller B, Smith AK, Hamburger S, Kumra S, Rapoport JL. | Lumping |
| 301. | Biol Psychiatry. 1999 Nov 15;46 :1418-28. doi: 10.1016/s0006-3223(99)00231-0. Childhood-onset schizophrenia: rare but worth studying. Nicolson R , Rapoport JL. | Review |
| 302. | Eur Child Adolesc Psychiatry. 1999;8 Suppl 1:I29-35. doi: 10.1007/pl00010688. Symptom dimensions in the course of childhood-onset schizophrenia. Bunk D , Eggers C, Klapal M. | Lumping |
| 303. | Eur Child Adolesc Psychiatry. 1999;8 Suppl 1:I21-8. doi: 10.1007/pl00010687. The ESSEN study of childhood-onset schizophrenia: selected results. Eggers C , Bunk D, Volberg G, Röpcke B. | No VEOS |
| 304. | Eur Child Adolesc Psychiatry. 1999;8 Suppl 1:I9-12. doi: 10.1007/pl00010685. Childhood-onset schizophrenia: a follow-up study. Rosenbaum Asarnow J , Tompson MC. | Lumping |
| 305. | Am J Psychiatry. 1999 Oct;156 :1650-2. doi: 10.1176/ajp.156.10.1650. Obstetrical complications and childhood-onset schizophrenia. Nicolson R , Malaspina D, Giedd JN, Hamburger S, Lenane M, Bedwell J, Fernandez T, Berman A, Susser E, Rapoport JL. | No Clinical |
| 306. | Am J Psychiatry. 1999 Oct;156 :1575-9. doi: 10.1176/ajp.156.10.1575. Clinical and neurobiological correlates of cytogenetic abnormalities in childhood-onset schizophrenia. Nicolson R , Giedd JN, Lenane M, Hamburger S, Singaracharlu S, Bedwell J, Fernandez T, Thaker GK, Malaspina D, Rapoport JL. | No Clinical |
| 307. | Biol Psychiatry. 1999 Oct 1;46 :892-8. doi: 10.1016/s0006-3223(99)00072-4. Childhood-onset schizophrenia: progressive brain changes during adolescence. Giedd JN , Jeffries NO, Blumenthal J, Castellanos FX, Vaituzis AC, Fernandez T, Hamburger SD, Liu H, Nelson J, Bedwell J, Tran L, Lenane M, Nicolson R, Rapoport JL. | No Clinical |
| 308. | Schizophr Res. 1999 Aug 17;38(2-3):93-9. doi: 10.1016/s0920-9964(99)00010-9. Childhood-onset schizophrenia and obstetric complications: a case--control study. Matsumoto H , Takei N, Saito H, Kachi K, Mori N. | No Clinical |
| 309. | Arch Gen Psychiatry. 1999 Jul;56 :649-54. doi: 10.1001/archpsyc.56.7.649. Progressive cortical change during adolescence in childhood-onset schizophrenia. A longitudinal magnetic resonance imaging study. Rapoport JL , Giedd JN, Blumenthal J, Hamburger S, Jeffries N, Fernandez T, Nicolson R, Bedwell J, Lenane M, Zijdenbos A, Paus T, Evans A. | No Clinical |
| 310. | Am J Psychiatry. 1999 Jul;156 :1065-8. doi: 10.1176/ajp.156.7.1065. Including children and adolescents with schizophrenia in medication-free research. Kumra S , Briguglio C, Lenane M, Goldhar L, Bedwell J, Venuchekov J, Jacobsen LK, Rapoport JL. | No Clinical |
| 311. | Biol Psychiatry. 1999 Jul 1;46 :8-18. doi: 10.1016/s0006-3223(99)00085-2. Elementary phenotypes in the neurobiological and genetic study of schizophrenia. Adler LE , Freedman R, Ross RG, Olincy A, Waldo MC. | Review |
| 312. | Biol Psychiatry. 1999 May 15;45 :1356-69. doi: 10.1016/s0006-3223(98)00349-7. Continuous-processing related ERPS in adult schizophrenia: continuity with childhood onset schizophrenia. Strandburg RJ , Marsh JT, Brown WS, Asarnow RF, Guthrie D, Harper R, Nuechterlein KH. | No Clinical |
| 313. | Am J Med Genet. 1999 Apr 16;88 :211-3. Apolipoprotein E alleles in childhood-onset schizophrenia. Fernandez T, Yan WL, Hamburger S, Rapoport JL, Saunders AM, Schapiro M, Ginns EI, Sidransky E. | No Clinical |
| 314. | Am J Med Genet. 1999 Apr 16;88 :188-99. Evidence for bilineal inheritance of physiological indicators of risk in childhood-onset schizophrenia. Ross RG , Olincy A, Harris JG, Radant A, Hawkins M, Adler LE, Freedman R. | No Clinical |
| 315. | J Abnorm Child Psychol. 1999 Feb;27 :35-49. doi: 10.1023/a:1022662323823. Exploratory eye movements to pictures in childhood-onset schizophrenia and attention-deficit/hyperactivity disorder (ADHD). | No Clinical |
| 316. | Mol Psychiatry. 1999 Jan;4 :58-63. doi: 10.1038/sj.mp.4000448. Detection of polyglutamine expansion in a new acidic protein: a candidate for childhood onset schizophrenia? Moriniere S , Saada C, Holbert S, Sidransky E, Galat A, Ginns E, Rapoport JL, Neri C. | No Clinical |
| 317. | J Abnorm Child Psychol. 1998 Oct;26 :367-80. doi: 10.1023/a:1021903923120. Components of visual search in childhood-onset schizophrenia and attention-deficit/hyperactivity disorder. Karatekin C , Asarnow RF. | Lumping |
| 318. | Encephale. 1998 Jul-Aug;24 :378-85. [Atypical neuroleptics in the child and adolescent]. Naja WJ , Reneric JP, Bouvard MP. | No Clinical |
| 319. | Mayo Clin Proc. 1998 Oct;73 :956-9. doi: 10.4065/73.10.956. Childhood-onset schizophrenia associated with parkinsonism in a patient with a microdeletion of chromosome 22. Krahn LE , Maraganore DM, Michels VV. | Case Report |
| 320. | Am J Psychiatry. 1998 Oct;155 :1376-83. doi: 10.1176/ajp.155.10.1376. Common pattern of cortical pathology in childhood-onset and adult-onset schizophrenia as identified by proton magnetic resonance spectroscopic imaging. Bertolino A , Kumra S, Callicott JH, Mattay VS, Lestz RM, Jacobsen L, Barnett IS, Duyn JH, Frank JA, Rapoport JL, Weinberger DR. | No Clinical |
| 321. | Psychiatry Res. 1998 Aug 17;80 :165-76. doi: 10.1016/s0165-1781(98)00061-4. Working memory in childhood-onset schizophrenia and attention-deficit/hyperactivity disorder. Karatekin C , Asarnow RF. | Lumping |
| 322. | J Magn Reson Imaging. 1998 Jul-Aug;8 :841-6. doi: 10.1002/jmri.1880080413. Preliminary study of frontal lobe 1H MR spectroscopy in childhood-onset schizophrenia. Thomas MA , Ke Y, Levitt J, Caplan R, Curran J, Asarnow R, McCracken J. | No Clinical |
| 323. | Mol Psychiatry. 1998 Jul;3 :321-7. doi: 10.1038/sj.mp.4000405. Large CAG/CTG repeats are associated with childhood-onset schizophrenia. Burgess CE , Lindblad K, Sidransky E, Yuan QP, Long RT, Breschel T, Ross CA, McInnis M, Lee P, Ginns EI, Lenane M, Kumra S, Jacobsen L, Rapoport JL, Schalling M. | No Clinical |
| 324. | Am J Psychiatry. 1998 Aug;155 :1074-9. doi: 10.1176/ajp.155.8.1074. Frequency and severity of enlarged cavum septi pellucidi in childhood-onset schizophrenia. Nopoulos PC , Giedd JN, Andreasen NC, Rapoport JL. | No Clinical |
| 325. | Psychiatry Res. 1998 May 8;78 :123-32. doi: 10.1016/s0165-1781(98)00015-8. HLA antigens in childhood onset schizophrenia. Jacobsen LK , Mittleman BB, Kumra S, Lenane MC, Barracchini KC, Adams S, Simonis T, Lee PR, Long RT, Sharp W, Sidransky E, Ginns EI, Rapoport JL. | No Clinical |
| 326. | Am J Psychiatry. 1998 May;155 :678-85. doi: 10.1176/ajp.155.5.678. Progressive reduction of temporal lobe structures in childhood-onset schizophrenia. Jacobsen LK , Giedd JN, Castellanos FX, Vaituzis AC, Hamburger SD, Kumra S, Lenane MC, Rapoport JL. | No Clinical |
| 327. | Nervenarzt. 1998 Mar;69 :238-42. doi: 10.1007/s001150050265. [The 5 factor model of childhood schizophrenia]. Klapal M , Eggers C, Bunk D, Koriath H. | No English |
| 328. | J Am Acad Child Adolesc Psychiatry. 1998 Apr;37 :377-85. doi: 10.1097/00004583-199804000-00015. Childhood-onset schizophrenia: an open-label study of olanzapine in adolescents. Kumra S , Jacobsen LK, Lenane M, Karp BI, Frazier JA, Smith AK, Bedwell J, Lee P, Malanga CJ, Hamburger S, Rapoport JL. | No Clinical |
| 329. | J Child Psychol Psychiatry. 1998 Jan;39 :101-13. Research update: childhood-onset schizophrenia: implications of clinical and neurobiological research. Jacobsen LK , Rapoport JL. | Review |
| 330. | J Am Acad Child Adolesc Psychiatry. 1998 Mar;37 :292-6. doi: 10.1097/00004583-199803000-00014. Brief report: association of sex chromosome anomalies with childhood-onset psychotic disorders. Kumra S , Wiggs E, Krasnewich D, Meck J, Smith AC, Bedwell J, Fernandez T, Jacobsen LK, Lenane M, Rapoport JL. | No Clinical |
| 331. | Am J Med Genet. 1998 Feb 7;81 :41-3. Chromosome 22q11.2 interstitial deletions among childhood-onset schizophrenics and "multidimensionally impaired". Yan W , Jacobsen LK, Krasnewich DM, Guan XY, Lenane MC, Paul SP, Dalwadi HN, Zhang H, Long RT, Kumra S, Martin BM, Scambler PJ, Trent JM, Sidransky E, Ginns EI, Rapoport JL. | No Clinical |
| 332. | J Abnorm Psychol. 1998 Feb;107 :97-108. doi: 10.1037//0021-843x.107.1.97. Attention deficits in childhood-onset schizophrenia: reaction time studies. Zahn TP , Jacobsen LK, Gordon CT, McKenna K, Frazier JA, Rapoport JL. | No Clinical |
| 333. | J Am Acad Child Adolesc Psychiatry. 1998 Feb;37 :221-7. doi: 10.1097/00004583-199802000-00016. Case series: spectrum of neuroleptic-induced movement disorders and extrapyramidal side effects in childhood-onset schizophrenia. Kumra S , Jacobsen LK, Lenane M, Smith A, Lee P, Malanga CJ, Karp BI, Hamburger S, Rapoport JL. | No Clinical |
| 334. | J Am Acad Child Adolesc Psychiatry. 1998 Jan;37 :91-9. doi: 10.1097/00004583-199801000-00022. "Multidimensionally impaired disorder": is it a variant of very early-onset schizophrenia? Kumra S , Jacobsen LK, Lenane M, Zahn TP, Wiggs E, Alaghband-Rad J, Castellanos FX, Frazier JA, McKenna K, Gordon CT, Smith A, Hamburger S, Rapoport JL. | Lumping |
| 335. | Psychiatry Res. 1997 Oct 31;75 :131-44. doi: 10.1016/s0925-4927(97)00050-4. Cerebral glucose metabolism in childhood onset schizophrenia. Jacobsen LK , Hamburger SD, Van Horn JD, Vaituzis AC, McKenna K, Frazier JA, Gordon CT, Lenane MC, Rapoport JL, Zametkin AJ. | No Clinical |
| 336. | Arch Gen Psychiatry. 1998 Jan;55 :90-2. doi: 10.1001/archpsyc.55.1.90. Childhood-onset schizophrenia. Bhatara VS, Gupta S, Flugsrud-Breckenridge M. | Case Report |
| 337. | Am J Psychiatry. 1997 Dec;154 :1663-9. doi: 10.1176/ajp.154.12.1663. Quantitative morphology of the cerebellum and fourth ventricle in childhood-onset schizophrenia. Jacobsen LK , Giedd JN, Berquin PC, Krain AL, Hamburger SD, Kumra S, Rapoport JL. | No Clinical |
| 338. | Arch Gen Psychiatry. 1997 Oct;54 :904-12. doi: 10.1001/archpsyc.1997.01830220020003. Autonomic nervous system markers of psychopathology in childhood-onset schizophrenia. Zahn TP , Jacobsen LK, Gordon CT, McKenna K, Frazier JA, Rapoport JL. | No Clinical |
| 339. | Arch Gen Psychiatry. 1997 Oct;54 :897-903. doi: 10.1001/archpsyc.1997.01830220013002. Childhood-onset schizophrenia. Progressive ventricular change during adolescence. Rapoport JL , Giedd J, Kumra S, Jacobsen L, Smith A, Lee P, Nelson J, Hamburger S. | No Clinical |
| 340. | J Immunol. 1997 Sep 15;159 :2994-9. Cerebrospinal fluid cytokines in pediatric neuropsychiatric disease. Mittleman BB , Castellanos FX, Jacobsen LK, Rapoport JL, Swedo SE, Shearer GM. | No Clinical |
| 341. | Indian J Psychiatry. 1997 Jul;39 :262-4. Use of clozapine in childhood schizophrenia. Srinivasan TN , Latha S. | Case Report |
| 342. | Br J Psychiatry. 1997 Jun;170:507-10. doi: 10.1192/bjp.170.6.507. An open trial of clozapine in neuroleptic-resistant childhood-onset schizophrenia. Turetz M , Mozes T, Toren P, Chernauzan N, Yoran-Hegesh R, Mester R, Wittenberg N, Tyano S, Weizman A. | No Clinical |
| 343. | J Child Psychol Psychiatry. 1997 May;38 :421-9. doi: 10.1111/j.1469-7610.1997.tb01527.x. Children with schizophrenia-spectrum disorders: thought disorder and communication problems in a family interactional context. Tompson MC , Asarnow JR, Hamilton EB, Newell LE, Goldstein MJ. | Lumping |
| 344. | Am J Psychiatry. 1997 May;154 :685-7. doi: 10.1176/ajp.154.5.685. Three-dimensional cortical morphometry of the planum temporale in childhood-onset schizophrenia. Jacobsen LK , Giedd JN, Tanrikut C, Brady DR, Donohue BC, Hamburger SD, Kumra S, Alaghband-Rad J, Rumsey JM, Rapoport JL. | No Clinical |
| **345.** | **Psychiatry Res. 1997 Apr 18;70 :1-7. doi: 10.1016/s0165-1781(97)03062-x. Pubertal development and onset of psychosis in childhood onset schizophrenia. Frazier JA , Alaghband-Rad J, Jacobsen L, Lenane MC, Hamburger S, Albus K, Smith A, McKenna K, Rapoport JL.** | **Included** |
| 346. | Fortschr Neurol Psychiatr. 1997 Apr;65 :154-70. doi: 10.1055/s-2007-996319. [Diagnostic and follow-up typological characteristics of early schizophrenia]. Eggers C , Klapal M. | No English |
| 347. | Psychiatry Res. 1997 Feb 7;68(2-3):77-86. doi: 10.1016/s0925-4927(96)03019-3. Quantitative magnetic resonance imaging of the corpus callosum in childhood onset schizophrenia. Jacobsen LK , Giedd JN, Rajapakse JC, Hamburger SD, Vaituzis AC, Frazier JA, Lenane MC, Rapoport JL. | No Clinical |
| 348. | Schizophr Bull. 1997;23 :105-17. doi: 10.1093/schbul/23.1.105. The long-term course of childhood-onset schizophrenia: a 42-year followup. Eggers C , Bunk D. | No Data |
| 349. | Am J Psychiatry. 1997 Jan;154 :69-74. doi: 10.1176/ajp.154.1.69. Cerebrospinal fluid monoamine metabolites in childhood-onset schizophrenia. Jacobsen LK , Frazier JA, Malhotra AK, Karoum F, McKenna K, Gordon CT, Hamburger SD, Lenane MC, Pickar D, Potter WZ, Rapoport JL. | No Clinical |
| 350. | Am J Psychiatry. 1997 Jan;154 :64-8. doi: 10.1176/ajp.154.1.64. Childhood-onset schizophrenia: biological markers in relation to clinical characteristics. Alaghband-Rad J , Hamburger SD, Giedd JN, Frazier JA, Rapoport JL. | No Clinical |
| 351. | Biol Psychiatry. 1996 Dec 15;40 :1222-9. doi: 10.1016/0006-3223(95)00625-7. Blink rate in childhood-onset schizophrenia: comparison with normal and attention-deficit hyperactivity disorder controls. Jacobsen LK , Hommer DW, Hong WL, Castellanos FX, Frazier JA, Giedd JN, Rapoport JL. | No Clinical |
| 352. | Arch Gen Psychiatry. 1996 Dec;53 :1090-7. doi: 10.1001/archpsyc.1996.01830120020005. Childhood-onset schizophrenia. A double-blind clozapine-haloperidol comparison. Kumra S , Frazier JA, Jacobsen LK, McKenna K, Gordon CT, Lenane MC, Hamburger SD, Smith AK, Albus KE, Alaghband-Rad J, Rapoport JL. | No Clinical |
| 353. | Biol Psychiatry. 1996 Dec 1;40 :1144-54. doi: 10.1016/S0006-3223(95)00630-3. Smooth pursuit eye movements in childhood-onset schizophrenia: comparison with attention-deficit hyperactivity disorder and normal controls. Jacobsen LK , Hong WL, Hommer DW, Hamburger SD, Castellanos FX, Frazier JA, Giedd JN, Gordon CT, Karp BI, McKenna K, Rapoport JL. | No Clinical |
| **354.** | **J Am Acad Child Adolesc Psychiatry. 1996 Jul;35 :950-8. doi: 10.1097/00004583-199607000-00023.Conversational repair in schizophrenic and normal children. Caplan R , Guthrie D, Komo S.** | **Included** |
| 355. | Arch Gen Psychiatry. 1996 Jul;53 :617-24. doi: 10.1001/archpsyc.1996.01830070065010. Brain anatomic magnetic resonance imaging in childhood-onset schizophrenia. Frazier JA , Giedd JN, Hamburger SD, Albus KE, Kaysen D, Vaituzis AC, Rajapakse JC, Lenane MC, McKenna K, Jacobsen LK, Gordon CT, Breier A, Rapoport JL. | No Clinical |
| 356. | Am J Psychiatry. 1996 Apr;153 :564-6. doi: 10.1176/ajp.153.4.564. Childhood-onset schizophrenia: brain MRI rescan after 2 years of clozapine maintenance treatment. Frazier JA , Giedd JN, Kaysen D, Albus K, Hamburger S, Alaghband-Rad J, Lenane MC, McKenna K, Breier A, Rapoport JL. | No Clinical |
| 357. | Am J Psychiatry. 1996 Mar;153 :355-61. doi: 10.1176/ajp.153.3.355. Temporal lobe morphology in childhood-onset schizophrenia. Jacobsen LK , Giedd JN, Vaituzis AC, Hamburger SD, Rajapakse JC, Frazier JA, Kaysen D, Lenane MC, McKenna K, Gordon CT, Rapoport JL. | No Clinical |
| **358.** | **J Am Acad Child Adolesc Psychiatry. 1995 Oct;34 :1273-83. doi: 10.1097/00004583-199510000-00012. Childhood-onset schizophrenia: the severity of premorbid course. Alaghband-Rad J , McKenna K, Gordon CT, Albus KE, Hamburger SD, Rumsey JM, Frazier JA, Lenane MC, Rapoport JL.** | **Included** |
| 359. | Psychiatry Clin Neurosci. 1995 Aug;49 :201-7. doi: 10.1111/j.1440-1819.1995.tb01885.x. Clinical features of childhood-onset schizophrenia with obsessive-compulsive symptoms during the prodromal phase. | No VEOS |
| **360.** | **Br J Psychiatry. 1995 Apr;166 :489-95. doi: 10.1192/bjp.166.4.489. Child and adolescent (juvenile onset) schizophrenia. A case control study of premorbid developmental impairments. Hollis C .** | **Included** |
| 361. | J Child Psychol Psychiatry. 1994 Nov;35 :1345-71. doi: 10.1111/j.1469-7610.1994.tb01280.x. Annotation: childhood-onset schizophrenia. Asarnow JR . | Review |
| 362. | J Clin Psychiatry. 1994 Sep;55 Suppl B:94-7. Plasma clozapine and haloperidol concentrations in adolescents with childhood-onset schizophrenia: association with response. Piscitelli SC , Frazier JA, McKenna K, Albus KE, Grothe DR, Gordon CT, Rapoport JL. | No Clinical |
| 363. | J Autism Dev Disord. 1994 Aug;24 :537-45. doi: 10.1007/BF02172134. Gordon CT , Krasnewich D, White B, Lenane M, Rapoport JL. Brief report: translocation involving chromosomes 1 and 7 in a boy with childhood-onset schizophrenia. | Case Report |
| 364. | J Am Acad Child Adolesc Psychiatry. 1994 Jul-Aug;33 :771-81. doi: 10.1097/00004583-199407000-00001. Childhood-onset schizophrenia: timely neurobiological research. McKenna K , Gordon CT, Rapoport JL. | Review |
| 365. | J Am Acad Child Adolesc Psychiatry. 1994 Jun;33 :658-63. doi: 10.1097/00004583-199406000-00006. An open trial of clozapine in 11 adolescents with childhood-onset schizophrenia. Frazier JA , Gordon CT, McKenna K, Lenane MC, Jih D, Rapoport JL. | No Clinical |
| 366. | J Am Acad Child Adolesc Psychiatry. 1994 Jun;33 :636-44. doi: 10.1097/00004583-199406000-00003. Looking for childhood-onset schizophrenia: the first 71 cases screened. McKenna K , Gordon CT, Lenane M, Kaysen D, Fahey K, Rapoport JL. | No Data |
| 367. | J Am Acad Child Adolesc Psychiatry. 1994 Jun;33 :605-15. doi: 10.1097/00004583-199406000-00001. Thought disorder in childhood. Caplan R . | Review |
| 368. | J Am Acad Child Adolesc Psychiatry. 1994 Jun;33 :651-7. doi: 10.1097/00004583-199406000-00005. Clozapine for early developmental delays with childhood-onset schizophrenia: protocol and 15-month outcome. Towbin KE , Dykens EM, Pugliese RG. | No Clinical |
| 369. | Psychophysiology. 1994 May;31 :272-81. doi: 10.1111/j.1469-8986.1994.tb02216.x. Reduced attention-related negative potentials in schizophrenic adults. Strandburg RJ , Marsh JT, Brown WS, Asarnow RF, Guthrie D, Higa J, Yee-Bradbury CM, Nuechterlein KH. | No Clinical |
| 370. | J Abnorm Child Psychol. 1994 Apr;22 :129-46. doi: 10.1007/BF02167896. Family-expressed emotion, childhood-onset depression, and childhood-onset schizophrenia spectrum disorders: is expressed emotion a nonspecific correlate of child psychopathology or a specific risk factor for depression? Asarnow JR , Tompson M, Hamilton EB, Goldstein MJ, Guthrie D. | No Clinical |
| 371. | Biol Psychiatry. 1994 Feb 15;35 :228-34. doi: 10.1016/0006-3223(94)91253-x. Blink rate in childhood schizophrenia spectrum disorder. Caplan R , Guthrie D. | No Clinical |
| 372. | J Am Acad Child Adolesc Psychiatry. 1994 Jan;33 :65-70. doi: 10.1097/00004583-199401000-00010. Clozapine treatment in very early onset schizophrenia. Mozes T , Toren P, Chernauzan N, Mester R, Yoran-Hegesh R, Blumensohn R, Weizman A. | No Clinical |
| 373. | Schizophr Bull. 1994;20 :727-45. doi: 10.1093/schbul/20.4.727. Childhood-onset schizophrenia: history of the concept and recent studies. Remschmidt HE , Schulz E, Martin M, Warnke A, Trott GE. | Review |
| 374. | Schizophr Bull. 1994;20 :697-712. doi: 10.1093/schbul/20.4.697. Childhood-onset schizophrenia: an NIMH study in progress. Gordon CT , Frazier JA, McKenna K, Giedd J, Zametkin A, Zahn T, Hommer D, Hong W, Kaysen D, Albus KE, et al. | No Clinical |
| 375. | Schizophr Bull. 1994;20 :631-46. doi: 10.1093/schbul/20.4.631. The clinical presentation of childhood-onset schizophrenia. Russell AT . | Review |
| 376. | Schizophr Bull. 1994;20 :599-617. doi: 10.1093/schbul/20.4.599. Childhood-onset schizophrenia: a followup study. Asarnow JR , Tompson MC, Goldstein MJ. | Review |
| 377. | Schizophr Bull. 1994;20 :591-7. doi: 10.1093/schbul/20.4.591. Childhood-onset schizophrenia: editors' introduction. Asarnow RF , Asarnow JR. | Review |
| 378. | New Dir Ment Health Serv. 1992 Summer;(54):71-5. doi: 10.1002/yd.23319925414. Childhood-onset schizophrenia; . Gordon CT . | Review |
| **379.** | **J Child Psychol Psychiatry. 1990 Nov;31 :1103-14. doi: 10.1111/j.1469-7610.1990.tb00849.x. Formal thought disorder in childhood onset schizophrenia and schizotypal personality disorder. Caplan R , Perdue S, Tanguay PE, Fish B.** | **Included** |
| **380.** | **Psychiatry Res. 1990 Feb;31 :169-77. doi: 10.1016/0165-1781(90)90119-p. Information processing deficits of schizophrenic children with formal thought disorder. Caplan R , Foy JG, Asarnow RF, Sherman T.** | **Included** |
| **381.** | **J Child Psychol Psychiatry. 1988 Nov;29 :865-78. doi: 10.1111/j.1469-7610.1988.tb00759.x. Symptom development in childhood onset schizophrenia. Watkins JM , Asarnow RF, Tanguay PE.** | **Included** |
| 382. | J Child Psychol Psychiatry. 1988 Nov;29 :825-38. doi: 10.1111/j.1469-7610.1988.tb00756.x. Parental communication deviance in childhood onset schizophrenia spectrum and depressive disorders. Asarnow JR , Goldstein MJ, Ben-Meir S. | No Clinical |
| 383. | J Am Acad Child Psychiatry. 1986 Sep;25 :601-14. doi: 10.1016/s0002-7138(09)60284-3. The search for the psychobiological substrate of childhood onset schizophrenia. Asarnow R, Sherman T, Strandburg R. | No Clinical |
| 384. | Child Dev. 1984 Feb;55 :249-61. Studies of visual information processing in schizophrenic children. Asarnow RF, Sherman T. | No Clinical |
|  |  |  |

Included: 18

Excluded: 366

- No Clinical: 189
- Review: 63
- No VEOS: 37
- Case Report: 33
- Lumping: 16
- Unrelated: 10
- No Data: 8
- No English: 7
- Letter: 3

**Supplementary Table 2**. Risk of bias assessment

|  | **Study** | **Risk of bias** | **Level** |
| --- | --- | --- | --- |
|  | *Cheng et al., 2021* | Selection | Low |
|  |  |  | Medium |
|  |  |  | High |
|  |  | Performance | Low |
|  |  |  | Medium |
|  |  |  | High |
|  |  | Detection | Low |
|  |  |  | Medium |
|  |  |  | High |
|  |  | Attrition | Low |
|  |  |  | Medium |
|  |  |  | High |
|  |  | Reporting | Low |
|  |  |  | Medium |
|  |  |  | High |
|  | *Galitzer et al., 2021* | Selection | Low |
|  |  |  | Medium |
|  |  |  | High |
|  |  | Performance | Low |
|  |  |  | Medium |
|  |  |  | High |
|  |  | Detection | Low |
|  |  |  | Medium |
|  |  |  | High |
|  |  | Attrition | Low |
|  |  |  | Medium |
|  |  |  | High |
|  |  | Reporting | Low |
|  |  |  | Medium |
|  |  |  | High |
|  | *Coulon et al., 2020* | Selection | Low |
|  |  |  | Medium |
|  |  |  | High |
|  |  | Performance | Low |
|  |  |  | Medium |
|  |  |  | High |
|  |  | Detection | Low |
|  |  |  | Medium |
|  |  |  | High |
|  |  | Attrition | Low |
|  |  |  | Medium |
|  |  |  | High |
|  |  | Reporting | Low |
|  |  |  | Medium |
|  |  |  | High |
|  | *Craddock et al., 2019* | Selection | Low |
|  |  |  | Medium |
|  |  |  | High |
|  |  | Performance | Low |
|  |  |  | Medium |
|  |  |  | High |
|  |  | Detection | Low |
|  |  |  | Medium |
|  |  |  | High |
|  |  | Attrition | Low |
|  |  |  | Medium |
|  |  |  | High |
|  |  | Reporting | Low |
|  |  |  | Medium |
|  |  |  | High |
|  | *Ordonez et al, 2016* | Selection | Low |
|  |  |  | Medium |
|  |  |  | High |
|  |  | Performance | Low |
|  |  |  | Medium |
|  |  |  | High |
|  |  | Detection | Low |
|  |  |  | Medium |
|  |  |  | High |
|  |  | Attrition | Low |
|  |  |  | Medium |
|  |  |  | High |
|  |  | Reporting | Low |
|  |  |  | Medium |
|  |  |  | High |
|  | *Greenstein et al., 2014* | Selection | Low |
|  |  |  | Medium |
|  |  |  | High |
|  |  | Performance | Low |
|  |  |  | Medium |
|  |  |  | High |
|  |  | Detection | Low |
|  |  |  | Medium |
|  |  |  | High |
|  |  | Attrition | Low |
|  |  |  | Medium |
|  |  |  | High |
|  |  | Reporting | Low |
|  |  |  | Medium |
|  |  |  | High |
|  | *David et al., 2011* | Selection | Low |
|  |  |  | Medium |
|  |  |  | High |
|  |  | Performance | Low |
|  |  |  | Medium |
|  |  |  | High |
|  |  | Detection | Low |
|  |  |  | Medium |
|  |  |  | High |
|  |  | Attrition | Low |
|  |  |  | Medium |
|  |  |  | High |
|  |  | Reporting | Low |
|  |  |  | Medium |
|  |  |  | High |
|  | *White T et al., 2004* | Selection | Low |
|  |  |  | Medium |
|  |  |  | High |
|  |  | Performance | Low |
|  |  |  | Medium |
|  |  |  | High |
|  |  | Detection | Low |
|  |  |  | Medium |
|  |  |  | High |
|  |  | Attrition | Low |
|  |  |  | Medium |
|  |  |  | High |
|  |  | Reporting | Low |
|  |  |  | Medium |
|  |  |  | High |
|  | *Anand A. Mattai et al., 2006* | Selection | Low |
|  |  |  | Medium |
|  |  |  | High |
|  |  | Performance | Low |
|  |  |  | Medium |
|  |  |  | High |
|  |  | Detection | Low |
|  |  |  | Medium |
|  |  |  | High |
|  |  | Attrition | Low |
|  |  |  | Medium |
|  |  |  | High |
|  |  | Reporting | Low |
|  |  |  | Medium |
|  |  |  | High |
|  | *Parthasarathy Biswas et al., 2006* | Selection | Low |
|  |  |  | Medium |
|  |  |  | High |
|  |  | Performance | Low |
|  |  |  | Medium |
|  |  |  | High |
|  |  | Detection | Low |
|  |  |  | Medium |
|  |  |  | High |
|  |  | Attrition | Low |
|  |  |  | Medium |
|  |  |  | High |
|  |  | Reporting | Low |
|  |  |  | Medium |
|  |  |  | High |
|  | *Ahmad Abu-Akel M.A et al, (2000)* | Selection | Low |
|  |  |  | Medium |
|  |  |  | High |
|  |  | Performance | Low |
|  |  |  | Medium |
|  |  |  | High |
|  |  | Detection | Low |
|  |  |  | Medium |
|  |  |  | High |
|  |  | Attrition | Low |
|  |  |  | Medium |
|  |  |  | High |
|  |  | Reporting | Low |
|  |  |  | Medium |
|  |  |  | High |
|  | *Frazier et al. (1997)* | Selection | Low |
|  |  |  | Medium |
|  |  |  | High |
|  |  | Performance | Low |
|  |  |  | Medium |
|  |  |  | High |
|  |  | Detection | Low |
|  |  |  | Medium |
|  |  |  | High |
|  |  | Attrition | Low |
|  |  |  | Medium |
|  |  |  | High |
|  |  | Reporting | Low |
|  |  |  | Medium |
|  |  |  | High |
|  | *Caplan et al. (1996)* | Selection | Low |
|  |  |  | Medium |
|  |  |  | High |
|  |  | Performance | Low |
|  |  |  | Medium |
|  |  |  | High |
|  |  | Detection | Low |
|  |  |  | Medium |
|  |  |  | High |
|  |  | Attrition | Low |
|  |  |  | Medium |
|  |  |  | High |
|  |  | Reporting | Low |
|  |  |  | Medium |
|  |  |  | High |
|  | *Alaghband-Rad et al. 1997* | Selection | Low |
|  |  |  | Medium |
|  |  |  | High |
|  |  | Performance | Low |
|  |  |  | Medium |
|  |  |  | High |
|  |  | Detection | Low |
|  |  |  | Medium |
|  |  |  | High |
|  |  | Attrition | Low |
|  |  |  | Medium |
|  |  |  | High |
|  |  | Reporting | Low |
|  |  |  | Medium |
|  |  |  | High |
|  | *Hollis et al. 1995* | Selection | Low |
|  |  |  | Medium |
|  |  |  | High |
|  |  | Performance | Low |
|  |  |  | Medium |
|  |  |  | High |
|  |  | Detection | Low |
|  |  |  | Medium |
|  |  |  | High |
|  |  | Attrition | Low |
|  |  |  | Medium |
|  |  |  | High |
|  |  | Reporting | Low |
|  |  |  | Medium |
|  |  |  | High |
|  | *Caplan et al. 1990* | Selection | Low |
|  |  |  | Medium |
|  |  |  | High |
|  |  | Performance | Low |
|  |  |  | Medium |
|  |  |  | High |
|  |  | Detection | Low |
|  |  |  | Medium |
|  |  |  | High |
|  |  | Attrition | Low |
|  |  |  | Medium |
|  |  |  | High |
|  |  | Reporting | Low |
|  |  |  | Medium |
|  |  |  | High |
|  | *Caplan et al 1989* | Selection | Low |
|  |  |  | Medium |
|  |  |  | High |
|  |  | Performance | Low |
|  |  |  | Medium |
|  |  |  | High |
|  |  | Detection | Low |
|  |  |  | Medium |
|  |  |  | High |
|  |  | Attrition | Low |
|  |  |  | Medium |
|  |  |  | High |
|  |  | Reporting | Low |
|  |  |  | Medium |
|  |  |  | High |
|  | *Watkins et al. 1988* | Selection | Low |
|  |  |  | Medium |
|  |  |  | High |
|  |  | Performance | Low |
|  |  |  | Medium |
|  |  |  | High |
|  |  | Detection | Low |
|  |  |  | Medium |
|  |  |  | High |
|  |  | Attrition | Low |
|  |  |  | Medium |
|  |  |  | High |
|  |  | Reporting | Low |
|  |  |  | Medium |
|  |  |  | High |
